# The Impact of Intra-Burst Energy Distribution on Volume Removal and Surface Quality in Laser Ablation of Stainless Steels Using Ultrashort Pulsed Lasers

**DOI:** 10.3390/ma19204267

**Published:** 2026-10-09

**Authors:** Dirk Obergfell, Flavio Soldera, Bahman Azarhoushang, Andrés Fabián Lasagni

**Affiliations:** 1 Institute for Advanced Manufacturing (KSF), Furtwangen University, Katharinenstrasse 2, 78532 Tuttlingen, Germany; 2 Institute of Manufacturing, Technische Universität Dresden, George-Bähr-Str. 3c, 01069 Dresden, Germany; 3 Department for Materials Science, EUSMAT, Saarland University, Campus D3.3, 66123 Saarbruecken, Germany; 4 Fraunhofer-Institut für Werkstoff-und Strahltechnik IWS, Winterbergstr. 28, 01277 Dresden, Germany

**Keywords:** ultra-short pulsed laser, stainless steel, burst mode, burst ablation, volume ablation, burst energy distribution

## Abstract

Stainless-steel alloys are widely used in applications requiring high corrosion resistance and mechanical durability. For embossing tool fabrication, high-volume material removal with high surface quality is essential. Laser-based ablation offers an alternative to micromilling by enabling material removal without mechanical contact, thereby eliminating cutting tool wear and facilitating the fabrication of complex geometries. However, productivity must be increased without degrading surface quality, which plays a critical role in embossing tool wear behavior and lifetime. This study investigates the ablation efficiency and surface quality of AISI 304, AISI 420, and AISI 316Ti stainless steels using a 250 fs pulsed laser. Different fluence levels and burst configurations, including MHz, GHz, and Bi-burst, are examined, focusing on the influence of intra-burst energy distribution on material removal rate and surface quality. Surface morphology and roughness are characterized by optical, confocal, and scanning electron microscopy. The results show that removal rate, ablation efficiency, and surface roughness are strongly affected by the intra-burst energy distribution (positive or negative slopes). The highest removal rate of 5.74 mm^3^ min^−1^ was obtained using MHz bursts, whereas GHz bursts enabled surface roughness values Sa below 1 µm. Negative energy slopes generally reduced surface roughness in isolated MHz and GHz burst modes, while Bi-burst processing provided smooth surfaces over a wider range of slope conditions. All alloys followed similar trends, with AISI 316Ti exhibiting greater susceptibility to microhole formation under certain burst conditions.

## 1. Introduction

Stainless-steel alloys play a crucial role across a wide range of industries, including medical, food, and tooling sectors [1,2,3]. Conventional micromilling is commonly employed for the fabrication of precision steel tools and molds due to its high dimensional accuracy and geometric flexibility. However, when processing hard and corrosion-resistant stainless steels, such as AISI 304, AISI 420, or AISI 316Ti, tool wear and limited achievable feature sizes restrict efficiency and surface quality [4]. In this frame, ultrashort-pulse (USP) microprocessing offers a competitive alternative to conventional micromilling for the production of embossing tools, especially for hard, difficult-to-machine stainless steels [5].

Embossing tools must meet high quality requirements, since their precision directly affects the accuracy and surface quality of the embossed parts [6,7]. In micromilling, material removal rates are relatively low, typically below 1 mm^3^ min^−1^, because the processing speed depends strongly on feature size and geometric complexity [4]. Smaller features require smaller tools, which are more susceptible to wear and breakage, thereby limiting the achievable machining speed [4,8]. In addition, processing speed and surface quality are affected by tool geometry, runout, and cutting forces [4,9]. These mechanical limitations can be avoided by USP laser processing, since material is removed without contact and without tool wear [10]. Moreover, USP laser processing enables high-resolution material removal with a limited heat-affected zone [11]. In addition, higher material removal rates can also be achieved. For example, Schille et al. reported a maximum removal rate of ~6 mm^3^ min^−1^ for stainless steel using a fs-pulsed laser at 32 W of laser power [12].

The high removal rates achievable with USP laser processing are mainly related to rapid energy deposition and limited heat diffusion. In USP laser ablation, energy transfer occurs through several ultrafast processes on different time scales. Initially, the laser energy is absorbed predominantly by the electron system of the material within tens to hundreds of femtoseconds, causing the electron temperature to rise significantly above that of the lattice and creating a state of strong electron–phonon nonequilibrium [13]. Subsequently, energy is transferred from the electrons to the lattice on the picosecond timescale via electron–phonon coupling, typically over 1 to several tens of picoseconds [13]. Thermal transport from the laser-heated surface into the bulk, primarily via electron conduction in metals, also contributes to energy dynamics and becomes especially relevant for high-repetition-rate bursts. In this context, a burst refers to a group of closely spaced laser pulses emitted as one pulse packet, where the total pulse energy is divided among several sub-pulses.

The temporal spacing between pulses within a burst determines how heat is accumulated. In the GHz regime (inter-pulse delays < 1 ns), subsequent pulses interact with the preheated or molten material, promoting cumulative effects. In the MHz regime (delays > 1 ns), partial cooling occurs between pulses, but cumulative heating can still enhance ablation efficiency and influences surface morphology [13]. Simulation studies have further shown that pulse delays in the picosecond range significantly affect energy absorption and crater formation, with plasma shielding observed for delays exceeding ~20 ps [14]. In addition, time-resolved experiments on GHz burst ablation of metals indicate that subsequent pulses can induce nanoparticle ejection and streaks via recoil pressure on molten material, in contrast to standard pulse ablation where particle ejection occurs at distinct timescales and velocities [15,16].

Besides the temporal pulse spacing, the applied fluence also determines the dominant material removal mechanism. USP ablation at near-threshold fluences is driven by direct vaporization and spallation, where rapid electron heating generates compressive stresses that relax into tensile stresses causing fracture and layer ejection. At higher fluences (>1 J·cm^−2^), material removal transitions to phase explosion, where superheating induces vapor bubble nucleation and the ejection of a liquid-vapor mixture. At sufficiently high fluences, both mechanisms can occur simultaneously, resulting in thermodynamic instability [17]. Therefore, optimizing removal rates while maintaining surface quality requires careful control of fluence and pulse distribution.

For optimizing laser ablation using burst mode, the most important parameters controlling the ablation rate, ablation efficiency, and resulting surface quality are the number of sub-pulses per burst, the intra-burst pulse separation time, and the temporal energy distribution among the individual sub-pulses. These parameters control how the total laser energy is temporally distributed, how successive pulses interact with the evolving material state, and how heat accumulation, plasma shielding, plume interaction, and surface formation develop during ablation [18,19,20,21,22,23,24,25,26]. Previous studies have shown that burst-mode irradiation can enhance both ablation efficiency and surface finish [19,27]. MHz bursts can exploit heat accumulation to increase removal efficiency, whereas GHz bursts may encounter plasma shielding but can improve surface quality [28]. Combining MHz and GHz bursts into Bi-bursts can balance ablation efficiency and surface quality by leveraging the advantages of both regimes [25,29]. Recent studies highlight that also the intra-burst energy distribution, i.e., the relative energy assigned to each sub-pulse within a burst, has a significant influence on the resulting ablation performance [30]. A decreasing pulse-energy distribution characterized by higher energy levels in the initial sub-pulses, followed by progressively lower energy in subsequent sub-pulses, has been shown to enhance ablation efficiency and stability [31]. Balage et al. reported that such a decreasing energy distribution increased ablation rates during USP drilling of glass, as early high-energy pulses efficiently generate an absorption-enhancing plasma while later, lower-energy pulses contribute to cleaning and gentle material removal without excessive heating [31].

Additionally, the material’s thermophysical properties such as reflectivity, electron heat capacity, and thermal conductivity can have an impact on how energy is absorbed and dissipated. Furthermore, the alloy composition and microstructure influence heat accumulation and plasma formation, stressing the need for material-specific optimization of burst parameters [32].

While different burst modes have been widely studied, the effect of intra-burst energy distribution on ablation performance and surface morphology on different stainless steels remains insufficiently explored. This work systematically investigates how different intra-burst energy distributions affect ablation rate, ablation efficiency, and surface roughness in AISI 304, AISI 420 and AISI 316Ti.

## 2. Materials and Methods

### 2.1. Materials

For the ablation experiments, 1 mm thick stainless-steel samples (HSM Stahl- und Metallhandel, Georgensgmuend, Germany) of AISI 304 (X5CrNi18-10), AISI 420 (X46Cr13), and AISI 316Ti (X6CrNiMoTi17-12-2) alloys were used as employed in previous studies [28,29,33]. These alloys are of special interest due to their importance within the medical and tooling industry. In addition, differences in thermal diffusivity may affect the ablation behavior of the alloys [34]. The relevant material properties, including the calculated thermal diffusivities, are listed in Table 1. AISI 420 shows the highest thermal diffusivity, which is approximately 2.4 times higher than those of AISI 304 and AISI 316Ti. This difference is primarily attributed to its higher thermal conductivity (30 W·m^−1^·K^−1^ compared with 15 W·m^−1^·K^−1^ for both austenitic grades), while its slightly lower density and specific heat capacity further increase the resulting thermal diffusivity. Furthermore, differences in microstructure and crystal structure may also affect the laser processing behavior. AISI 420 is a martensitic steel, whereas AISI 304 and AISI 316Ti are austenitic steels.

After the laser process, the samples were stored under atmospheric conditions.

### 2.2. Laser Ablation Experiments

Laser ablation experiments were carried out using a five-axis laser workstation (GFMS LP400u, Geneva, Switzerland) employed in previous studies [28,29,33]. The system was equipped with a solid-state ultra-short-pulsed laser source (LightConversion Carbide CB-3, Vilnius, Lithuania), emitting laser radiation at a wavelength of 1030 nm. The bursts of pulses were generated by splitting the main pulse into sub-pulses in the MHz and GHz range. Furthermore, the slope of the energy distribution within one burst was adjusted.

The laser has a maximum average power of 40 W with a maximal pulse energy *E_p_* of 200 µJ. The laser beam was deflected on the surface using a galvanometer scanner system (Scanlab Excelliscan, Puchheim, Germany) equipped with an f-theta lens having a focal distance of 160 mm. This optical setup leads to a beam diameter *ω*_0_ of approximately 38 µm at the material surface.

The process parameters were selected based on prior optimization studies aiming to achieve high ablation rates while maintaining good surface quality [28,29,34,35]. The previous study showed that relatively high pulse overlaps (~90%) in combination with high scanning speeds (3–5 m·s^−1^) resulted in better surface finish. In addition, short pulse durations have been shown to enhance ablation efficiency [29]. Consequently, a pulse duration of 250 fs was chosen for this study.

The different burst configurations were compared with reference experiments carried out without burst mode, referred to as standard single-pulse processing. For each parameter set, square areas of 2 mm × 2 mm were irradiated repeatedly to produce cavities, as established in previous investigations [29,34]. Each area was scanned 150 times and each scan of the complete area is referred to as one layer. The scanning speed was 3 m·s^−1^ and the repetition rate was set to 200 kHz. The used hatch distance was 5.0 µm.

The removal rate per applied energy (*Q*′ [µm^3^·µJ^−1^]) and per time (*Q*) [mm^3^·min^−1^] was calculated using Equations (1) and (2) as follows [26].

(1) $$Q^{\prime }=\frac{hP_{x}P_{y}f_{L}}{N_{l}P_{av}}$$

(2) $$Q=\frac{c_{V}}{v_{scan}N_{p}N_{sl}}$$

where *h* is the height of the ablated volume (step height), *P_x_* and *P_y_* describe the spacing between laser pulses in x and y direction, *f_L_* repetition rate, *N_l_* the number of layers, *N_sl_* the number of lines per layer, *N_p_* is the number of pulses per scan line, c_V_ trench volume (calculated from confocal images), and *P_av_* the average power. The scan direction was rotated 79° after each layer.

For the burst parameters, MHz, GHz and Bi-burst modes were used. The Bi-burst mode is defined as a combination of both MHz and GHz bursts. For the GHz burst, the intra-burst pulse separation time was 450 ps, corresponding to 2.22 GHz. In case of MHz burst mode, the intra-burst pulse separation time was 17 ns, leading to a frequency of 65 MHz. The pulse energy of the individual sub-pulses in a burst is defined as a fraction of the total energy that would be delivered by a single pulse. For example, in a three-pulse GHz burst with a total energy equivalent to a 100 µJ single pulse, each sub-pulse carries approximately one-third of the energy (~33 µJ) if the sub-pulses are identical.

**Figure 1:**
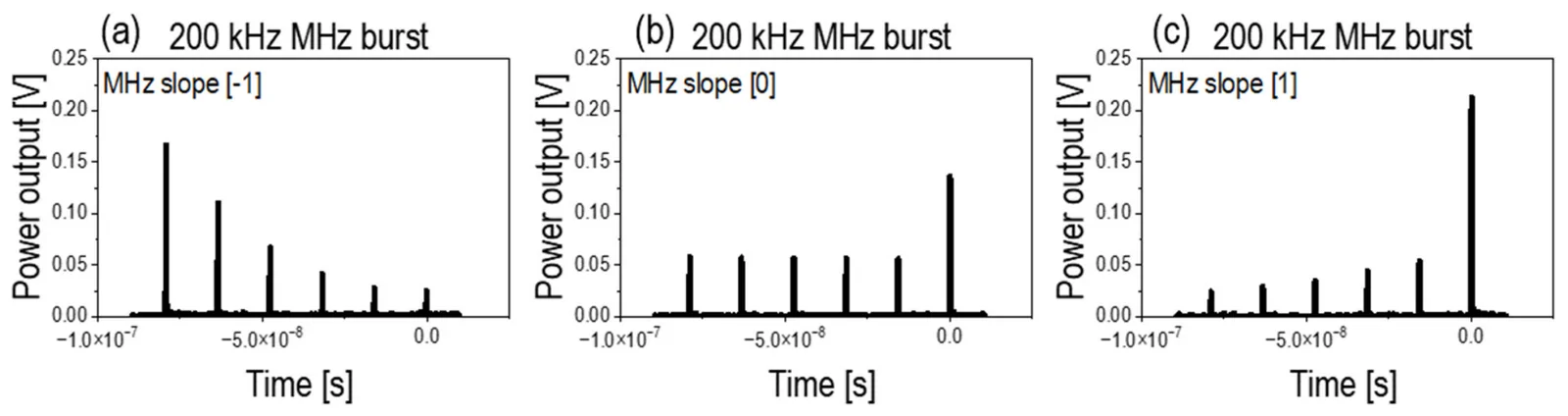
Oscilloscope measurements of 6 sub-pulse MHz bursts with negative slope (−1) (**a**), six sub-pulse MHz bursts with 0 slope (**b**) and six sub-pulse MHz bursts with positive slope (+1) (**c**).

In the case of Bi-burst operation, where both MHz and GHz bursts are combined, the total pulse energy is distributed between the two burst regimes. For instance, with a total energy of 100 µJ, a three-pulse MHz burst delivers ~33 µJ per pulse, and each of the subsequent GHz sub-pulses would carry a fraction of that (e.g., ~11 µJ per pulse in a three-pulse GHz sequence nested within each MHz pulse). When the burst slope is varied, the total energy remains constant, but its distribution among the sub-pulses changes. For MHz and GHz bursts, the number of sub-pulses per burst was varied from 3 to 10. In Bi-burst mode, MHz and GHz pulse sequences were combined, with the investigated configurations defined by the respective numbers of MHz and GHz sub-pulses. For all burst modes, three intra-burst energy distributions with slopes of −1, 0, and +1 were investigated. Figure 1 shows representative oscilloscope measurements illustrating the energy distribution for burst mode with different burst distributions. A fast photodiode (InGaAs, EOT-3500, Coherent, Traverse City, MI, USA) with a rise/fall time of <25 ps was used for signal detection, and measurements were recorded using a high-bandwidth oscilloscope (Keysight MSO-X 6004A, Santa Rosa, CA, USA, 6 GHz bandwidth). These measurements capture pulses with the same numbers of sub-pulses per burst and distinct intra-burst energy distribution in the nanosecond (ns) range, corresponding to MHz burst modes, as described before. In addition, the measurements show how the temporal energy distributions change when the burst slope is adjusted. The decrease in pulse energy for the negative slope (Figure 1a) as well as the increase in pulse energy for the positive slope (Figure 1c) are not strictly linear. Similarly, the nominal zero-slope condition (Figure 1b) does not result in a uniform energy distribution, but the final sub-pulse exhibits a significantly higher energy than the preceding sub-pulses. This deviation from the nominal distributions is characteristic of the laser source used in this study.

To identify the most suitable processing parameters for embossing tool fabrication, both a high ablation rate and high surface quality must be achieved. Therefore, a cost–function *c_f_* was defined to identify parameter sets that maximize material removal (efficiency and ablation rate) while minimizing surface roughness. This function combines all three criteria into one dimensionless value using weighting factors to reflect their relative importance:

(3) $$c_{f}={(1-w}_{1}\cdot \frac{A_{i}-A_{min}}{A_{max}-A_{min}})+{(1-w}_{2}\cdot \frac{B_{i}-B_{min}}{B_{max}-B_{min}})+w_{3}\cdot \frac{C_{i}-C_{min}}{C_{max}-C_{min}}$$

where *A* is the ablation efficiency [µm^3^·µJ^−1^], *B* the ablation rate [mm^3^·min^−1^], *C* the surface roughness [µm], *w*_1_, *w*_2_ and *w*_3_ the weighting factors for the ablation efficiency, the ablation rate and the surface quality, respectively. The parameters are normalized to the observed minimum and maximum values, allowing a direct comparison between process parameters despite different physical units. This concept has been recently used in other studies regarding laser-based surface treatments [36].

### 2.3. Surface Characterization

For analyzing the ablated volume of the produced cavities, a confocal microscope (Keyence VK-3000, Osaka, Japan) was utilized with a 20× microscope objective with NA 0.46, resulting in a lateral resolution of ~0.7 µm. The vertical resolution was 4 nm. The open-source Python software package version 0.15.0 Surfalize was employed to analyze the depth, volume, and surface roughness of the cavities, enabling standardized analysis for a large set of confocal images [25].

Surface quality was also examined with an optical microscope with 100× magnification (Keyence VHX-7000, Osaka, Japan). Furthermore, scanning electron microscope (SEM) images were taken on selected samples (Crossbeam 550 L Zeiss, Oberkochen, Germany) to better evaluate the produced surface topology. In addition, electron backscatter diffraction (EBSD) tests were performed with the software EDAX OIM Analysis (EDAX, Mahwah, NJ, USA) using a Helios G4 PFIB CXe (Thermo-Fisher Scientific, Eindhoven, The Netherlands) detector.

## 3. Results and Discussion

### 3.1. Impact of Burst Slope on Ablation Efficiency and Rate, and Surface Roughness

**Figure 2:**
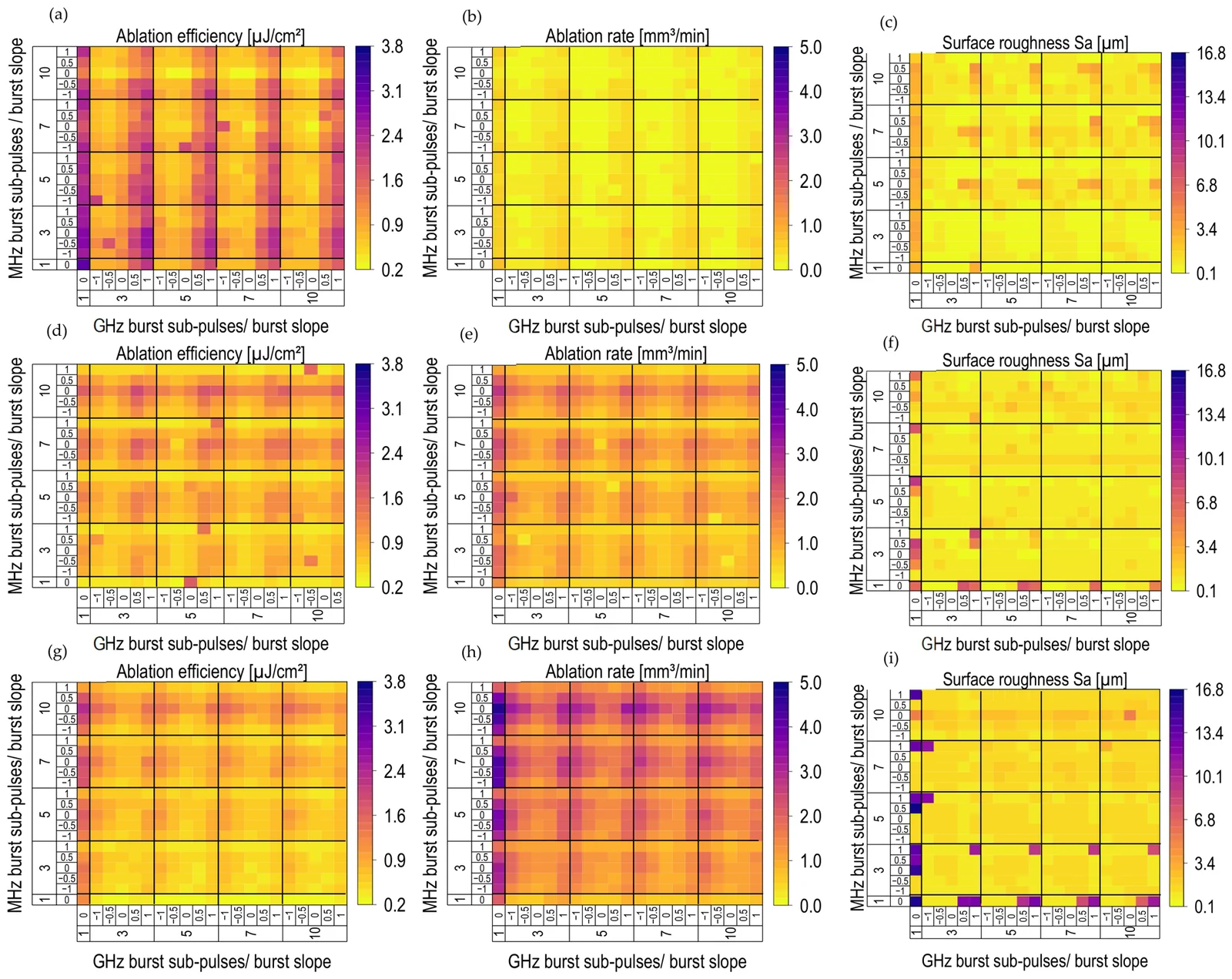
Heat maps of ablation efficiency (**a**,**d**,**g**), ablation rate (**b**,**e**,**h**), and surface roughness Sa (**c**,**f**,**i**) for stainless steel AISI 304 under various burst parameters at three fluence levels: top row, 1.7 J·cm^−2^; middle row, 8.5 J·cm^−2^; bottom row, 17.0 J·cm^−2^. Corresponding maps for AISI 420 and AISI 316Ti are provided in Supplementary Material Section (Figures S1 and S2). All other used processing parameters are described in the Materials and Methods Section. The reported values represent the mean of measurements performed at 3 different positions within each processed area.

**Figure 3:**
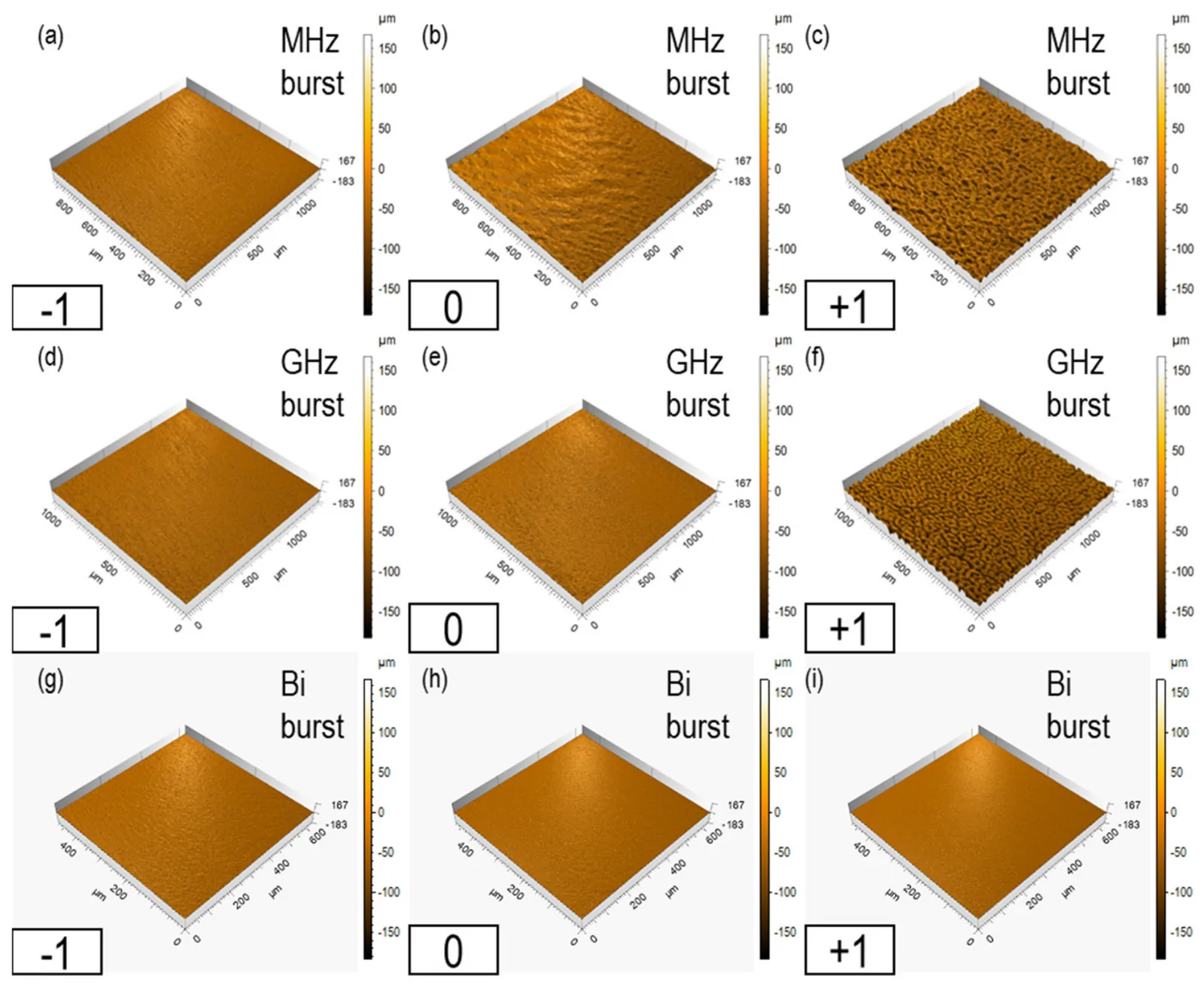
Confocal images of laser-processed AISI 304 surfaces obtained using MHz (**a**–**c**), GHz (**d**–**f**), and Bi-burst (**g**–**i**) modes with burst slopes of −1, 0, and +1 at a fluence of 1.7 J·cm^−2^. All other processing parameters are given in the Materials and Methods Section.

**Figure 4:**
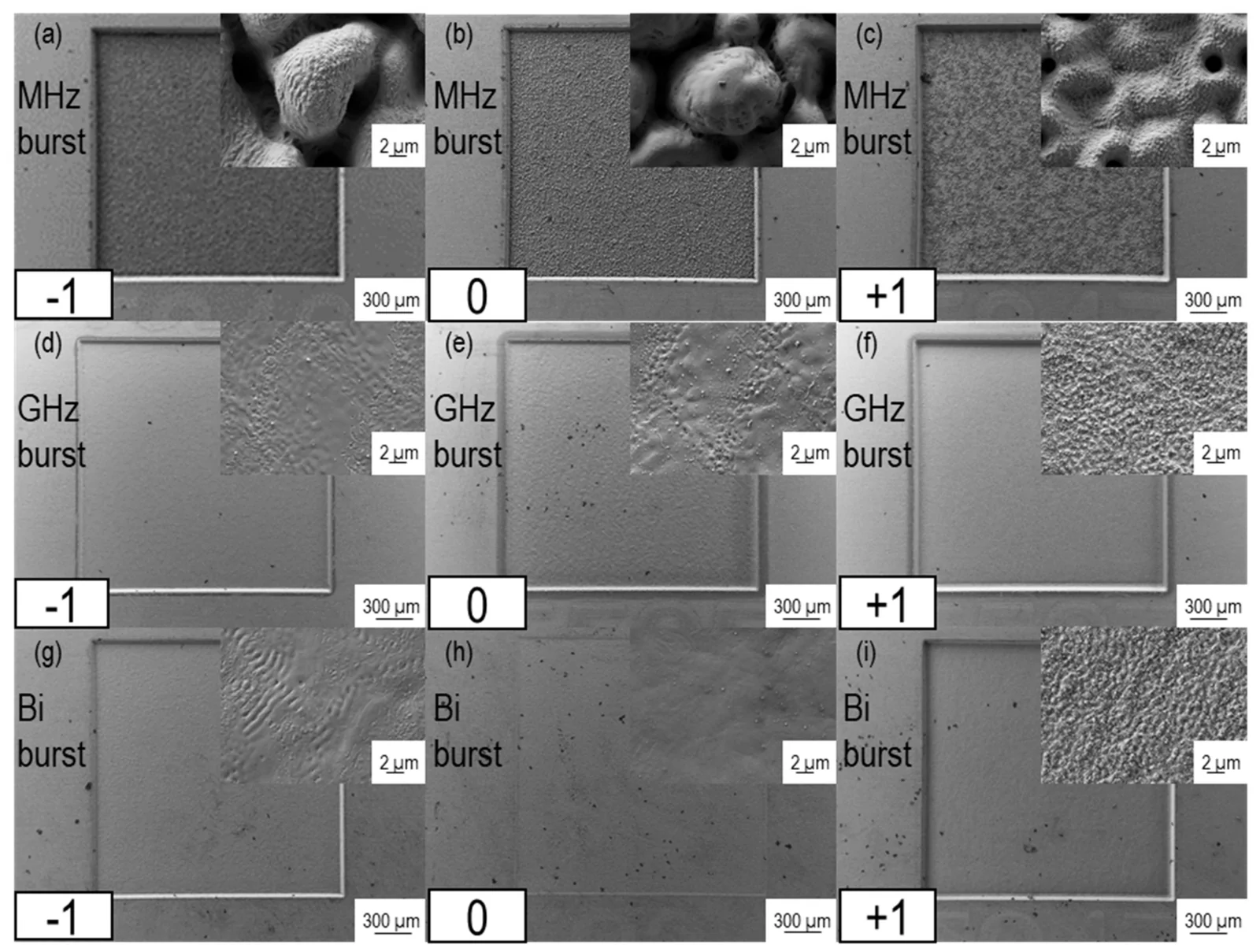
SEM images of cavities produced on AISI 304 steel using MHz (**a**–**c**), GHz (**d**–**f**), and Bi-burst (**g**–**i**) modes. The burst slope, corresponding to the temporal energy distribution within the burst, is arranged in columns and varied between −1, 0, and +1, as indicated at the top of the figure. The experiments were performed using 10 sub-pulse MHz and 10 sub-pulse GHz bursts at a constant fluence of approximately 1.7 J·cm^−2^. Parameters other than the applied fluence are described in materials and methods. SEM images with the same parameters for AISI 420 and AISI 316Ti are depicted in the Supplementary Material Section (Figures S5 and S6).

**Figure 5:**
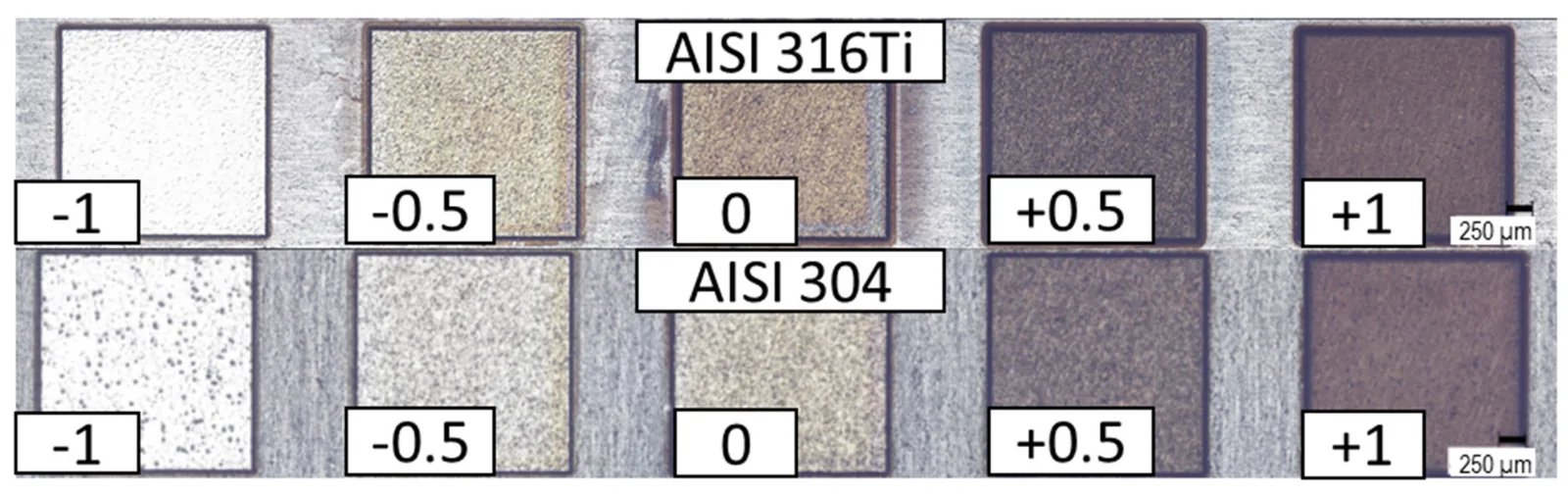
Optical microscope images of laser-processed AISI 304 (**top**) and AISI 316Ti (**bottom**) surfaces obtained at fluence 1.7 J cm^−2^ for intra-burst energy slopes ranging from –1 to +1. Parameters other than fluence are described in materials and methods.

**Figure 6:**
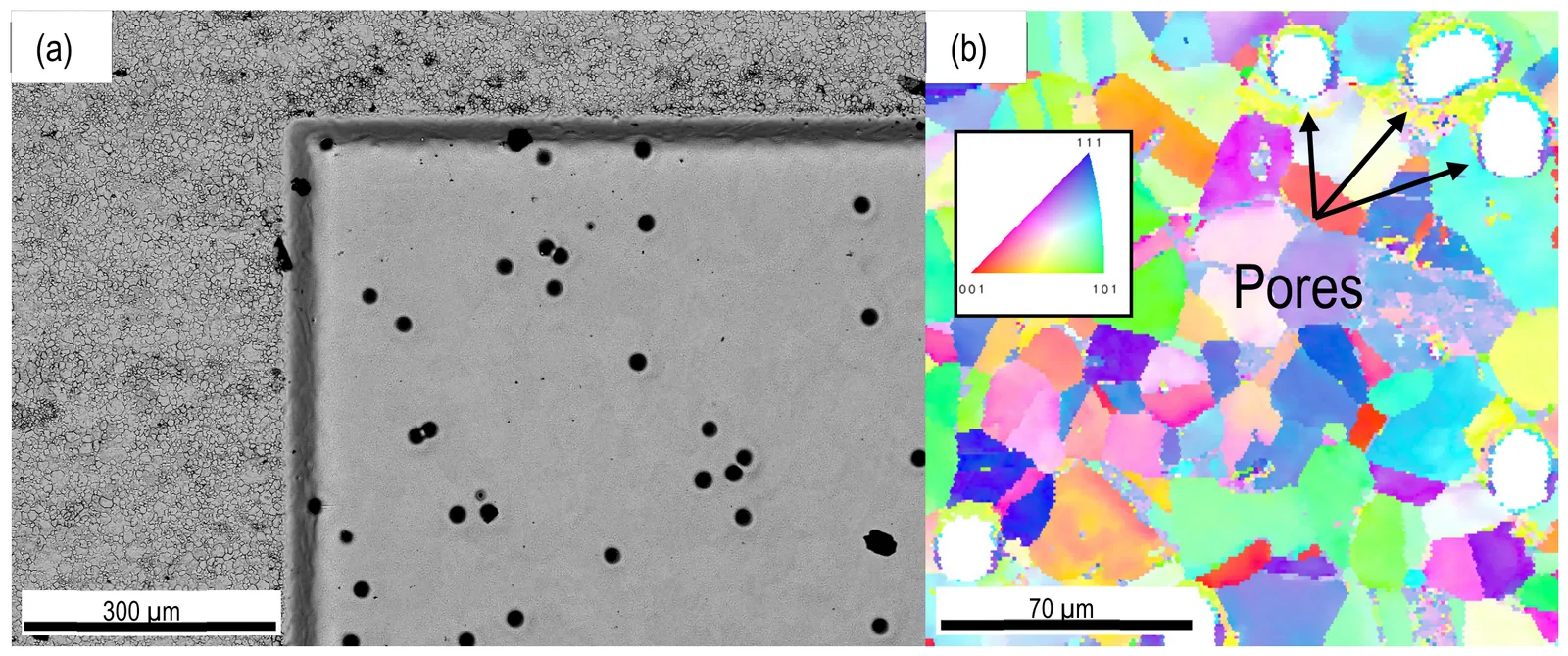
Microstructural characterization of AISI 316Ti using SEM and EBSD. The SEM image (**a**) shows a processed area (see Figure 5, for −1 slope) with distributed pores. The EBSD inverse pole figure (IPF) map with higher magnification (**b**) illustrates the crystallographic orientation of austenitic grains. Pores without diffraction signal appear as non-indexed areas.

In the first experimental series, 2 mm × 2 mm areas were irradiated to study the effect of intra-burst energy distribution on the ablation behavior of AISI 304, AISI 420, and AISI 316Ti. Figure 2 presents heat maps of ablation efficiency (Figure 2a,d,g), ablation rate (Figure 2b,e,h) and surface roughness (Figure 2c,f,i) as a function of the laser fluence on AISI 304. Corresponding maps for AISI 420 and AISI 316Ti are provided in Supplementary Material Section, in Figures S1 and S2. The vertical-axis shows MHz burst sub-pulses of 1, 3, 5, 7, and 10, each combined with variations in burst slope from −1 to +1 (see Experimental Section for nomenclature). Three different fluence levels were investigated (1.7 J·cm^−2^ (Figure 2a–c), 8.5 J·cm^−2^ (Figure 2d–f) and 17.0 J·cm^−2^ (Figure 2g–i)). To provide visual context for the parametric trends discussed below, representative confocal, optical, and SEM images of the corresponding ablated surfaces are later presented in Section 3.2 (Figure 3, Figure 4, Figure 5 and Figure 6).

The obtained results demonstrate that the burst slope has a significant influence on ablation efficiency, ablation rate and surface quality, in addition to the general effect of the burst type (MHz, GHz or Bi-burst) which was reported in previous studies [28,33]. In all investigated cases employing isolated MHz or GHz bursts, a positive burst slope resulted in higher surface roughness at the bottom of the produced cavities compared to either a zero or negative slopes. For instance, in Figure 2f, at a fluence of 8.5 J·cm^−2^ using a 7-sub-pulse MHz burst, a positive +1-slope lead to a surface roughness of 7.68 µm (Sa), whereas for a negative −1 slope it was 0.85 µm. Similarly, for a 7-sub-pulse GHz burst under the same fluence, the measured surface roughness was 6.24 µm (Sa) with a positive +1 slope and 0.56 µm Sa with a negative −1 slope. Although the slope-dependent trend was observed for both burst regimes, GHz bursts generally resulted in lower surface roughness than MHz bursts, consistent with previous studies. In general, it can be seen that GHz bursts resulted in lower surface roughness than MHz bursts which is in agreement was already discussed in with previous research [28,29,33]. The minimum value was obtained with a slope of −1 at 8.5 J·cm^−2^ using a 7-sub-pulse GHz burst. This trend became increasingly pronounced at higher fluence levels. A possible explanation for this behavior is that the higher energy of the initial pulses compensates for plasma and particle shielding losses while simultaneously preheating the material. The subsequent pulses, having lower energy, then promote the formation of a smoother very thin melt layer on the surface [37]. The application of Bi-burst configurations moderated this effect, leading to reduced surface roughness even for positive slopes. The lowest surface roughness (Sa) observed across all parameters at mid-fluence regime (8.5 J·cm^−2^) was 0.43 µm, achieved with a Bi-burst configuration of 10 sub-pulse MHz burst slope +1/3 sub-pulse GHz burst slope +1. A plausible explanation is that the MHz component carries out the primary material removal and preheating of the surface, while the GHz component induces a shallow melt redistribution by enhanced heat accumulation that smooths the residual asperities [38,39].

Regarding ablation efficiency, the heatmap in Figure 2a reveals a strong dependence on both the MHz and GHz burst sub-pulse numbers as well as the burst slope. The efficiency reaches its highest value of ~ 3 µm^3^·µJ^−1^ in the dark purple regions, that are mainly with only MHz burst or a low amount of GHz burst. In contrast, the lowest efficiency, around 1.2 µm^3^·µJ^−1^, is observed in the bright yellow areas, that occur at high number of MHz and GHz burst. A significant influence of the burst slope is observed for both MHz and GHz bursts. For example, at 5 GHz sub-pulses, a positive slope (+1) yields an ablation efficiency of 2 µm^3^·µJ^−1^, whereas a negative slope (−1) results in only 0.76 µm^3^·µJ^−1^. At low fluence as well as by using single-pulses (no burst), the ablation process is more efficient (e.g., 3.2 µm^3^ µJ^−1^) than using burst mode.

The comparison of the heatmaps at fluences of 1.7 J·cm^−2^ (Figure 2a–c), 8.5 J·cm^−2^ (Figure 2d–f), and 17.0 J·cm^−2^ (Figure 2g–i) highlights how the MHz and GHz burst parameters influence ablation efficiency under different energy conditions. At the lowest fluence of 1.7 J·cm^−2^, the highest ablation efficiency was obtained without burst mode (single-pulse) or with a low number of MHz sub-pulses. At higher fluences, the highest efficiencies were obtained with larger MHz or GHz burst sub-pulses, as shown for instance in Figure 2g for 10 sub-pulses MHz bursts with zero slope (~2 µm^3^·µJ^−1^). As fluence increases to 8.5 J·cm^−2^ and 17.0 J·cm^−2^, however, these variations become less pronounced and the overall efficiency decreases. This suggests that at these higher fluences, the influence of MHz and GHz burst operation modes diminishes, as thermal and plasma effects dominate the ablation process [40,41].

Specifically, the GHz burst number and slope primarily control the sub-pulse spacing on very short timescales, affecting how efficiently energy is deposited before significant plasma formation occurs. At lower fluences, optimal GHz slopes allow constructive energy accumulation and efficient ablation. At higher fluences, excessive energy leads to plasma shielding, reducing the effectiveness of GHz control. Conversely, the MHz burst number and slope, which act on longer temporal scales, influence the overall heat accumulation and material response between burst trains. At high fluence, increasing the MHz burst number or slope tends to promote thermal saturation and melt formation, further lowering ablation efficiency.

With respect to the ablation rate, the highest values were obtained using 7 to 10 sub-pulses in MHz bursts with 0 to −0.5 slopes, particularly in the medium (Figure 2e) and high-fluence (Figure 2i) regimes. The maximum ablation rate of 5.74 mm^3^·min^−1^ was achieved at a zero slope. Similarly to the ablation efficiency, the higher removal rate compared to the non-burst condition can be attributed to operation near the optimal fluence and heat accumulation within the MHz burst. Consecutive pulses increase the local surface temperature, reduce the effective ablation threshold, and thereby improve material removal. In addition, the final high-energy pulses provide extra energy that can partly compensate for losses caused by plasma shielding or particle ejection.

In contrast, GHz bursts generally resulted in lower ablation rates, with only minor variations observed as a function of the applied burst slope. Interestingly, in this regime, the lowest ablation rate occurred at a slope of 0. For example, in Figure 2i, at a fluence of 17.0 J·cm^−2^, the ablation rate was 0.80 mm^3^·min^−1^ for a positive +1 slope, 0.58 mm^3^·min^−1^ for the zero slope, and 0.69 mm^3^·min^−1^ for a negative −1 slope. In the GHz-burst regime, the ordering of pulse energies significantly influences ablation efficiency. When all pulses have equal energy (slope = 0), a constant plasma forms and persists throughout the burst, leading to maximum cumulative shielding and the lowest ablation rate [15,42]. A negative slope mitigates this effect by concentrating energy in the first pulses, which remove material before a dense plasma fully develops. However, subsequent weaker pulses mainly interact with the plasma or molten layer, contributing little additional removal [37,43]. Conversely, a positive slope delivers higher energy after the plasma has partially expanded, allowing improved energy coupling and slightly higher ablation rates [37].

However, when GHz bursts were employed in Bi-burst configurations, the ablation rate increased, particularly for positive GHz slopes. Moreover, increasing the number of MHz bursts within the Bi-burst mode consistently enhanced the ablation performance. For example, a combination of 10 sub-pulse MHz bursts and 7-sub-pulse GHz bursts with slopes of 0 and −1 achieved an ablation rate of 3.22 mm^3^ min^−1^.

The contradictory observation, that positive slopes enhance ablation in isolated GHz bursts, whereas negative GHz slopes maximize ablation in Bi-burst mode, can be explained by the different temporal contexts of the GHz sub-packets. In isolated GHz bursts, the low repetition rate (e.g., 200 kHz) allows significant cooling and plume dissipation between bursts. As a result, each burst interacts with a largely reset surface. In this case, concentrating energy in the later pulses of the GHz burst (positive slope) is advantageous, as these pulses couple more efficiently to a surface that has already been modified by the preceding sub-pulses. In Bi-burst mode, however, the higher MHz repetition rate leads to cumulative heating and incomplete plume dissipation between bursts. This results in a pre-heated surface and increased plasma presence at the arrival of each GHz packet. Under these conditions, concentrating energy in the early pulses of the GHz burst (negative slope) is more effective, as material removal occurs before plasma shielding becomes significant. The subsequent lower-energy pulses then contribute to melt relaxation rather than further plasma generation.

Therefore, the difference arises from the level of inter-burst thermal accumulation: while isolated bursts benefit from delayed energy delivery, Bi-burst operation favors early energy deposition within each GHz packet. (e.g., the 10 sub-pulse MHz bursts/7-sub-pulse GHz bursts, with 0 slope/−1 slope case yielding 3.22 mm^3^·min^−1^). Similar trends in ablation rate, ablation efficiency, and surface quality were observed for all investigated materials (AISI 304, AISI 316Ti, and AISI 420) and is shown in the Supplementary Material Section (Figures S1 and S2).

### 3.2. Impact of Burst Slope on Surface Morphology

To assess the effect of laser parameters on surface morphology, confocal microscopy, digital optical imaging, and scanning electron microscopy (SEM) were used to compare the surface features produced under different intra-burst energy distributions within each burst configuration. Confocal measurements, shown in Figure 3, reveal clear differences in surface topography depending on the applied intra-burst energy distribution for AISI 304 (corresponding images for AISI 420 and AISI 316Ti are depicted in the Supplementary Material Section, Figures S3 and S4). This was briefly presented in the previous section, when discussing the effect of process parameters on the observed surface roughness of the produced cavities.

A consistent trend was observed for both MHz and GHz burst modes (Figure 3a–f). Negative slopes produce relatively smooth surfaces (Figure 3a,d,g), whereas positive slopes lead to clearly developed periodic structures and increased roughness (Figure 3c,f,i). Surfaces generated with a positive slope exhibit increased roughness, which is more pronounced in the GHz burst regime. Similar correlations between burst energy distribution in microprocessed surface roughness have been documented in ultrafast laser processing studies [44]. The different effects of the intra-burst slopes can be attributed to the temporal sequence of energy deposition. With a negative slope, the highest-energy sub-pulses interact with a comparatively unmodified surface, whereas with a positive slope, the highest-energy sub-pulses interact with an already heated and potentially molten surface. The latter can promote heat accumulation and melt displacement, contributing to increased surface roughness, while plasma and vapor generated by preceding sub-pulses may additionally shield subsequent pulses and reduce effective energy coupling [15]. The zero slope represents an intermediate condition with a more balanced energy deposition over the burst.

In contrast, for Bi-burst processing (Figure 3g–i), the overall surface morphology appears more uniform across slope conditions, and variations in roughness are less distinct compared to isolated burst modes. This indicates that the combined MHz-GHz excitation moderates the slope-dependent roughening effect [45].

SEM images in Figure 4 further shows the micro- and nanostructure features induced by different burst modes and intra-burst energy slopes. For GHz bursts (Figure 4d–f), a negative slope (Figure 4d) predominantly leads to the formation of a resolidified surface layer, indicating enhanced melt layer formation generated by heat accumulation. In contrast, a positive slope results in a ripple-like nanostructure (Figure 4f).

For MHz bursts (Figure 4a–c), ripple structures are observed under both negative (Figure 4a) and positive slope conditions (Figure 4c), indicating that the longer intra-burst separation allows sufficient time for surface cooling and re-excitation, thereby supporting LIPSS (laser induced periodic surface structures) formation across different slope configurations. The smooth surface observed at 0 slope in the GHz regime (Figure 4e) is attributed to heat accumulation and melt formation, which suppress the preservation of fine periodic nanostructures such as LIPSS. Similar effects have already been reported in previous studies [46].

In the case of Bi-burst processing (Figure 4g–i), the same general trend observed for isolated bursts can be observed. Negative slopes lead to pronounced melt formation with only minor LIPSS development, whereas positive slopes promote LIPSS formation. This indicates that, even under combined MHz-GHz excitation, the relative intra-burst energy distribution remains a governing factor for the balance between melt-driven smoothing and interference-driven nanostructuring.

Despite similar ablation rates and surface roughness across all investigated alloys, pronounced parameter-dependent microhole formation was only observed for AISI 316Ti material. Optical microscopy images (Figure 5) reveal clear differences in surface morphology as a function of the intra-burst energy slope for AISI 304 and AISI 316Ti. For both materials, surfaces generated with negative slopes (Figure 5, see −1, −0.5) exhibit a bright, reflective appearance, whereas positive slopes (Figure 5, see +0.5, +1) lead to progressively darker surfaces. However, a strong microhole formation is observed exclusively in AISI 316Ti, particularly at negative slopes. With increasing slope, a gradual transition from high to reduced reflectivity occurs, accompanied by a decrease in microhole density.

The observed changes in reflectivity are directly linked to differences in surface microstructure, as confirmed by the SEM images in Figure 4 and described in the previous section. This distinction is highly relevant for applications requiring superior surface quality and is consistent with previous studies that correlate laser parameter variations with surface reflectivity and visual contrast [47,48].

As mentioned before, only AISI 316Ti exhibits pronounced parameter-dependent microhole formation. To further investigate the origin of the microholes, EBSD analysis was performed. Figure 6 shows the processed region analyzed by SEM (Figure 6a) and the EBSD-IPF mapping (Figure 6b).

The SEM image reveals a heterogeneous distribution of pores across the laser treated surface. Within the analyzed region, the corresponding IPF map indicates that these microholes occur predominantly within grains corresponding to crystallographic orientations near ⟨101⟩ (see colored yellow-green grains). In these regions, locally reduced image quality (IQ) values are observed due to the absence of diffraction patterns inside the microholes. In contrast, grains with ⟨111⟩ and ⟨001⟩ orientations remain largely unaffected. These observations indicate a possible orientation dependence of microhole formation. However, analysis over larger areas and a larger number of grains would be required to establish a statistically significant crystallographic correlation. Similar orientation-dependent effects during ultrashort pulse laser processing have been reported in the literature by Ackerl et al., where anisotropic ablation behavior led to preferential formation of cone-like protrusions [49]. Such behavior can be attributed to crystallographic differences in the interaction between the laser radiation and individual grains. Different crystal planes exhibit different planar atomic densities, surface energies, electronic structures, and slip systems. These factors can influence local optical absorption, electron–phonon energy transfer, melting behavior, and defect formation [50,51,52,53] In addition, orientation-dependent thermal transport may influence how efficiently the deposited energy is dissipated from the irradiated surface layer.

Under GHz burst irradiation, several pulses deposit energy within a very short time. As a result, even small crystallographic differences in optical absorption and heat dissipation can produce different local temperature rises in neighboring grains. The observed selectivity therefore suggests that microhole formation is governed by the combined effect of crystallographic orientation and the temporal energy distribution within the burst.

For a negative burst slope, the highest pulse energies occur at the beginning of the burst. This may promote rapid local heating and vaporization before plasma shielding, melt formation, or surface modification significantly reduce the effective energy coupling. Grains oriented close to ⟨101⟩ may therefore reach the conditions for microhole initiation more readily than grains oriented close to ⟨111⟩ or ⟨001⟩. Once initiated, subsequent lower-energy pulses can further interact with the modified surface geometry and contribute to hole deepening. This interpretation is consistent with grain-dependent effects reported during femtosecond laser processing of polycrystalline nickel by Sedao et al. [54].

### 3.3. Fabrication of an Embossing Stamp Tool

Based on the results obtained above, selected laser parameters were applied to fabricate an embossing stamp demonstrator. The design of this tool requires a combination of high ablation rate, ensuring competitiveness with micromilling, with a low surface roughness, with Sa below ~1 µm, to ensure functional performance and long tool lifetime. However, these two requirements are partly conflicting, since process parameters that enable high material removal typically lead to increased surface roughness (see Section 3.1 and Section 3.2), and parameters that produce smoother surfaces usually result in lower productivity. Therefore, a two-step process strategy was developed, consisting of a high-removal step for efficient bulk material ablation followed by a finishing step to reduce the surface roughness. The needed process parameters were chosen using a cost-function analysis, similar to recent studies on laser-based surface treatment of metallic alloys (see experimental section) [36,55].

In the first step, the cost–function was weighted primarily toward maximizing the ablation rate, since the main objective was rapid bulk material removal. Surface roughness was included as a secondary criterion to avoid excessively rough topographies, while ablation efficiency was considered with a lower but still relevant weight to account for process economy. Accordingly, the weighting factors for Step 1 were set to w_1_ = 2 (ablation efficiency), w_2_ = 5 (ablation rate), and w_3_ = 3 (surface roughness). The weights therefore represent an application-specific prioritization of the three performance criteria for the intended two-step roughing and finishing strategy. The cost–function analysis shown in Table 2 indicates that the best compromise between the above discussed parameters can be achieved using a 7-sub-pulse MHz burst with a zero slope using a fluence of 17.0 J·cm^−2^. This parameter set resulted in a material removal rate of 5.09 mm^3^·min^−1^ and an ablation efficiency of 2.12 µm^3^·µJ^−1^, while producing a surface roughness Sa of 1.48 µm.

In Step 2, since surface roughness became the main selection criterion, the parameter with the lowest surface roughness was selected. Accordingly, a 10-sub-pulse GHz burst with a slope of −1 at a fluence of 1.7 J·cm^−2^ was selected as the finishing parameter set, as indicated in Table 3.

**Figure 7:**
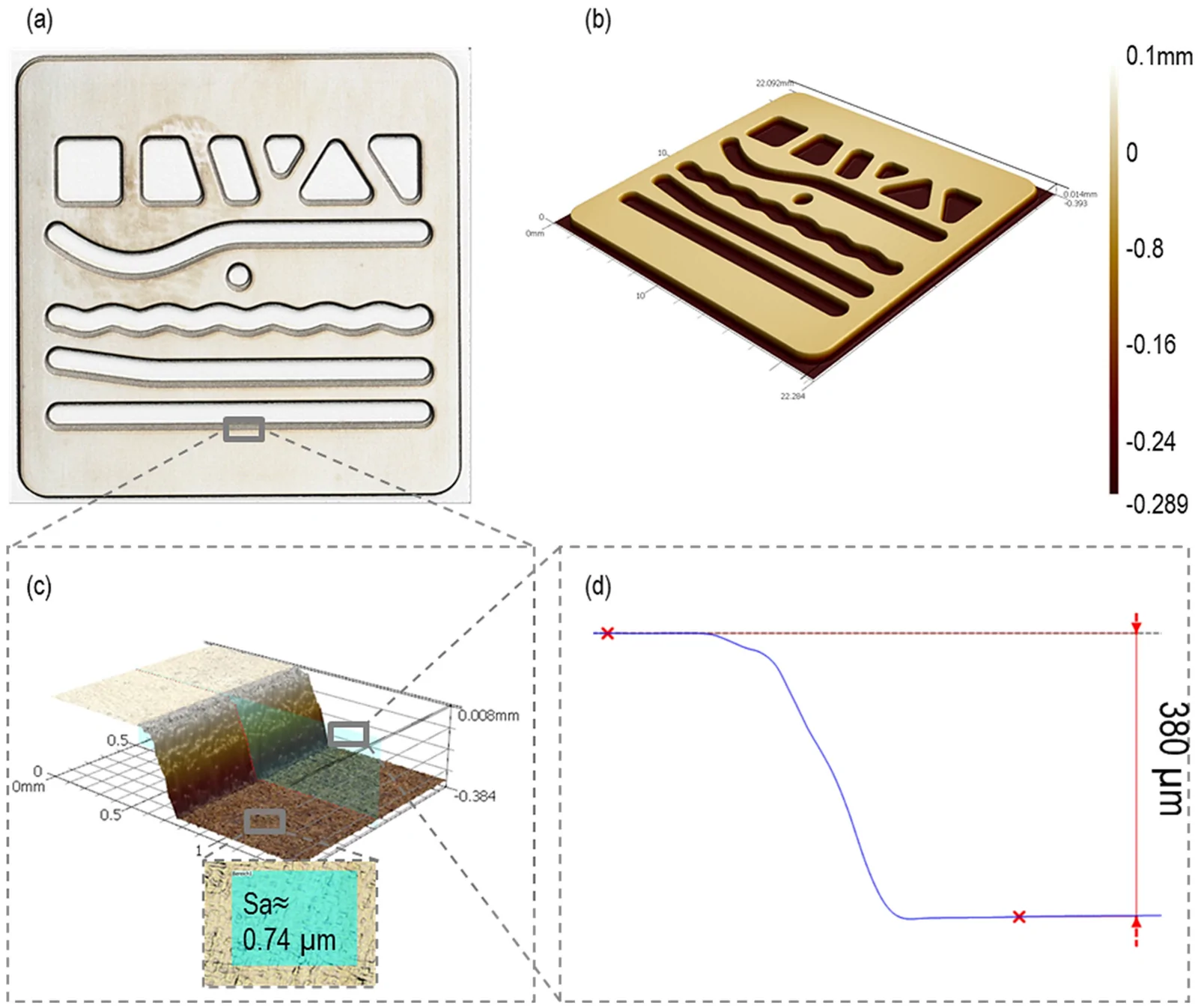
Laser-fabricated embossing stamp in AISI 420 stainless steel. (**a**) Optical image of the engraved demonstrator tool. (**b**) Three-dimensional confocal height map showing the overall geometry and depth distribution of the structured area. (**c**) Magnified 3D surface topography of the highlighted region, illustrating the surface morphology within the engraved channel (Sa ≈ 0.74 µm). (**d**) Representative cross-sectional height profile used to determine the engraving depth (≈350 µm).

Using the processing steps described above and the parameters selected through the cost-function analysis, the embossing stamp was fabricated. This demonstrator includes different laser-ablated microfeatures, as rectangular pockets, inclined slots, triangular and circular features, curved channels, wavy channels, and straight grooves. Figure 7a shows an optical image of the fabricated stamp. The confocal 3D topography in Figure 7b visualizes the overall geometry, and depth distribution of the ablated structures. A selected groove was further analyzed in detail as indicated in Figure 7c, where the evaluated surface area and cross-sectional measurement position are marked for assessing the local ablation depth and surface quality. The extracted line profile in Figure 7d was used to quantify the groove depth.

In the isolated parameter study, this finishing condition produced a surface roughness of Sa = 0.31 µm after 150 layers. In the two-step demonstrator process, however, the same finishing parameter set was applied to the roughened surface generated during Step 1, which had an initial roughness of Sa = 1.48 µm. Under these conditions, the finishing step reduced the final surface roughness to Sa = 0.71 µm. Although this value is higher than the roughness obtained in the isolated parameter study (starting from a surface having a surface roughness Sa of ~0.8 µm), it remains below 1 µm and therefore fulfills the surface-quality requirement for the embossing application. The difference in final roughness is therefore attributed to the different initial surface conditions prior to finishing. The achieved removal rate of 5.09 mm^3^·min^−1^ during roughing is approximately five times higher than the typical removal rates of ~1 mm^3^·min^−1^ reported for micromilling of comparable precision features [4].

## 4. Conclusions

In this work, the intra-burst energy distribution within MHz, GHz, and Bi-bursts was investigated as an additional degree of freedom for controlling material removal efficiency, removal rate, and surface quality during laser ablation of stainless-steel alloys. A detailed analysis of the ablated surfaces revealed pronounced differences in removal behavior and surface characteristics depending on the applied burst mode and intra-burst energy distribution.

The energy distribution within the burst had a pronounced effect on the ablation rate and surface roughness. For instance, at a fluence of 8.5 J·cm^−2^ using isolated MHz bursts containing 10 sub-pulses, the removal rate increased from 1.13 mm^3^·min^−1^ at a slope of −1 to a maximum of 2.72 mm^3^·min^−1^ at zero slope, before decreasing to 1.80 mm^3^·min^−1^ at a slope of +1. By contrast, for the corresponding isolated GHz-bursts configuration, the influence of the slope was comparatively small. Nevertheless, a negative slope led to the highest removal rate of 0.6 mm^3^·min^−1^. Furthermore, for Bi-burst processing with 10 sub-pulses MHz and 10 sub-pulses GHz, the energy-distribution slopes within the burst again had a relevant influence. The ablation rate varied from 0.45 mm^3^·min^−1^ using positive MHz burst and zero GHz burst, to 1.91 mm^3^·min^−1^ using zero MHz burst and positive GHz burst slope.

The performed analysis demonstrated that ablation efficiency is significantly influenced by burst mode and intra-burst energy distribution. Maximum efficiency (~2.5 µm^3^·µJ^−1^) occurred only for specific burst configurations, indicating a nonlinear relationship between intra- and inter-burst pulse structuring. At low fluence (1.7 J·cm^−2^), the ablation efficiency was highly sensitive to both MHz and GHz parameters, whereas at higher fluences (8.5–17 J·cm^−2^), this dependence became less pronounced as thermal and plasma effects increasingly dominated, thereby reducing the influence of fine temporal burst shaping.

Regarding surface roughness, the burst slope also showed a significant influence. At a fluence of 8.5 J·cm^−2^ and a 7-sub-pulse MHz burst, a positive slope produced a surface roughness of 7.68 µm (Sa), whereas a negative slope produced only 0.85 µm. Furthermore, when using isolated GHz bursts, a negative slope, e.g., at 7-sub-pulse GHz permitted to obtain surfaces with a reduced roughness up to 0.56 µm. This indicates that GHz bursts provided smoother surfaces compared to MHz bursts under similar conditions. Additionally, Bi-burst processing reduced surface roughness even at positive slopes, with a minimum (Sa) of 0.43 µm achieved at 8.5 J·cm^−2^ (10 MHz +1/3 GHz +1). This effect resulted from primary ablation by the MHz pulses and concurrent surface smoothing by GHz-driven melt redistribution.

For AISI 316Ti, negative intra-burst slopes led to pronounced pitting features that deteriorated surface quality at the microscale. EBSD analysis revealed possible crystallographic selectivity, with microholes forming predominantly in ⟨101⟩-oriented grains. This suggests that microhole formation is promoted by orientation-dependent energy coupling and heat accumulation during ablation.

## Figures and Tables

**Table 1:** Material properties of the used stainless-steel alloys in this study (extracted from the manufacturer data sheets).

Thermal Diffusivity α [m^2^·s^−1^]	Density ρ [kg·m^−3^]	Specific Heat Capacity c [J·kg^−1^·K^−1^]	Thermal Conductivity k [W·m^−1^·K^−1^]	Chemical Composition	Material Type
8.47 × 10^−6^	7700	460	30	C 0.50%, Si ≤ 1.00%, Mn ≤ 1.00%, P ≤ 0.040%, S ≤ 0.015%, Cr 14.50%, Mo ≤ 0.50%.	AISI 420
3.80 × 10^−6^	7900	500	15	C ≤ 0.07%, Si ≤ 1.00%, Mn ≤ 2.00%, P ≤ 0.045%, S ≤ 0.03%, Cr 19.50%, Ni 10.50%, N ≤ 0.11%.	AISI 304
3.75 × 10^−6^	8000	500	15	C ≤ 0.08%, Si ≤ 1.00%, Mn ≤ 2.00%, P ≤ 0.04%, S ≤ 0.03%, Cr 18.50%, Ni 13.50%, Mo 2.50%, Ti 0.70%.	AISI 316Ti

**Table 2:** Cost–function analysis identifying the process-parameter combinations with the lowest scores, corresponding to high ablation efficiency and ablation rate and low surface roughness. For clarity, only three best-performing parameter sets are shown. The complete analysis is provided in the Supplementary Material Section in Table S1.

Cost Function	Sa [µm]	Ablation Rate [mm^3^·min^−1^]	Ablation Efficiency [µm^3^·µJ^−1^]	Speed [mm/s]	GHz Burst Slope	MHz Burst Slope	GHz Burst	MHz Burst	Pulse Width [fs]	Frequency [kHz]	Fluence [J·cm^−2^]
0.87	1.487	5.09	2.12	3000	0	0	1	7	258	200	17
1.01	2.866	5.74	2.39	3000	0	0	1	10	258	200	17
1.34	1.596	4.68	1.95	3000	0	−0.5	1	10	258	200	17

**Table 3:** Cost–function analysis identifying the process-parameter combinations with the lowest scores, corresponding to minimum surface roughness. For clarity, only the three best-performing parameter sets are shown. The roughness parameters for all other parameter combinations can be found in Supplementary Material Section in Table S1.

Sa [µm]	Ablation Rate [mm^3^·min^−1^]	Ablation Effic. [µm^3^·µJ^−1^]	Speed	GHz Burst Slope	MHz Burst Slope	GHz Burst	MHz Burst	Pulse Width [fs]	Frequency [kHz]	Fluence [J·cm^−2^]
0.31	0.15	0.63	3000	−1	0	10	1	258	200	1.7
0.35	0.16	0.65	3000	−1	0	7	1	258	200	1.7
0.51	0.19	0.8	3000	−1	0	5	1	258	200	1.7

## Data Availability

The data presented in this study are available in the Supplementary Material of this article.

## Supplementary Materials

The following supporting information can be downloaded at: https://www.mdpi.com/article/10.3390/ma19204267/s1.
