# Supplementary material for: The Impact of Intra-Burst Energy Distribution on Volume Removal and Surface Quality in Laser Ablation of Stainless Steels Using Ultrashort Pulsed Lasers
Source: Materials (Basel). 2026 Oct 9;19(20):4267. doi: 10.3390/ma19204267 (PMC13706513; doi:10.3390/ma19204267)
Supplement: Supplementary file 1 [file materials-19-04267-s001.zip › materials-4561147-supplementary.pdf]

Article

Supplementary material

The Impact of Intra-Burst Energy Distribution on Volume Removal and Surface Quality in Laser Ablation of Stainless Steels Using Ultrashort Pulsed Lasers

Dirk Obergfell[1,2,*], Flavio Soldera[4], Bahman Azarhoushang[1], Andrés Fabián Lasagni[2,3]

[1] Institute for Advanced Manufacturing (KSF), Furtwangen University, Katharinenstrasse 2, 78532 Tuttlingen, Germany

[2] Institute of Manufacturing, Technische Universität Dresden, George-Bähr-Str. 3c, 01069 Dresden, Germany

[3] Department for Materials Science, Functional Materials, Saarland University, Campus D3.3, Saarbrücken 66123, Germany

[4] Fraunhofer-Institut für Werkstoff- und Strahltechnik IWS, Winterbergstr. 28, 01277 Dresden, Germany

* Correspondence: dirk.obergfell@mailbox.tu-dresden.de

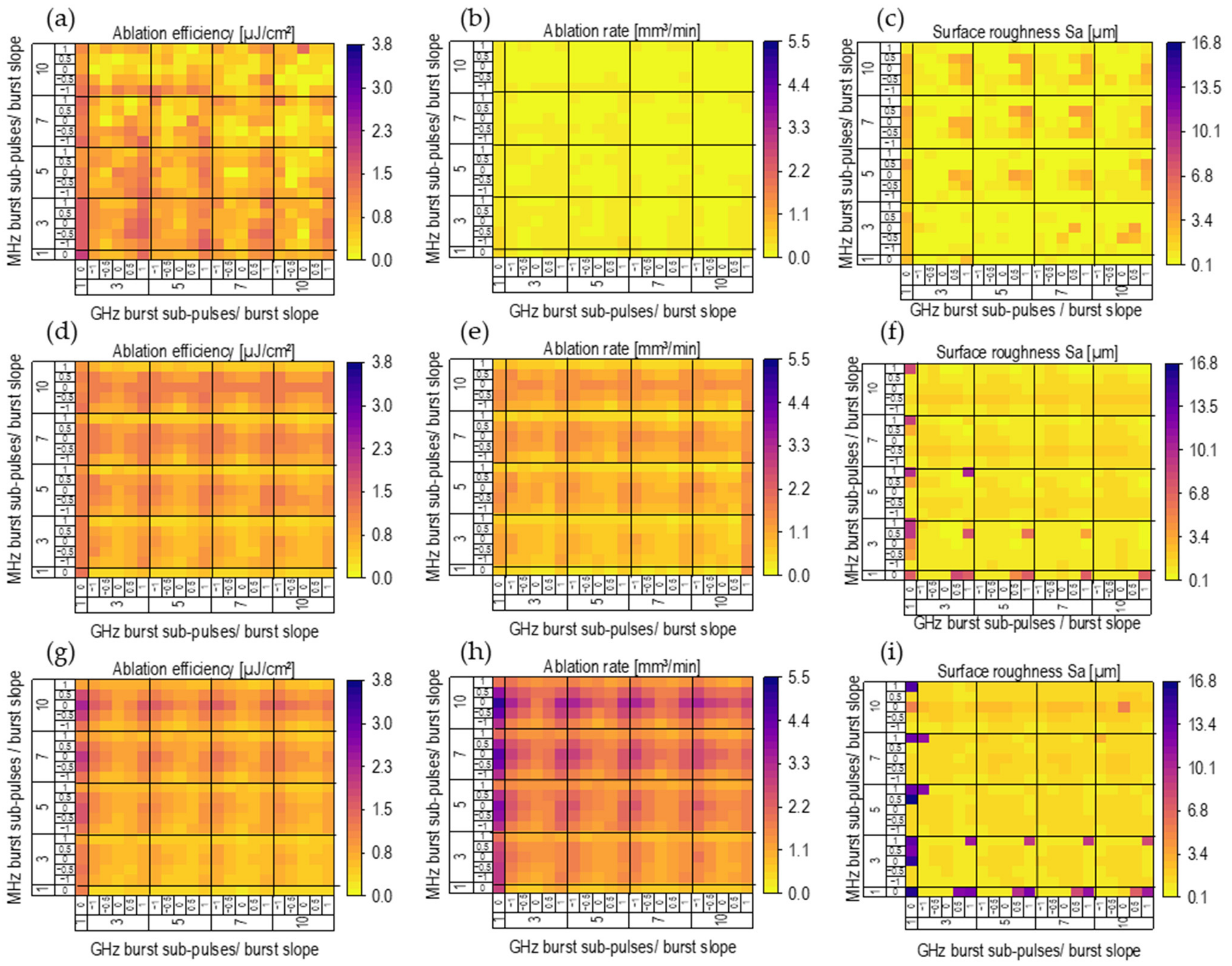

Figure S1. Heat maps of ablation efficiency (**a**, **d**, **g**), ablation rate (**b**, **e**, **h**), and surface roughness Sa (c, f, i) for stainless steel AISI 420 under various burst parameters at three fluence levels: top row, 1.7 J·cm^{-2}; middle row, 8.5 J·cm^{-2}; bottom row, 17.0 J·cm^{-2}.

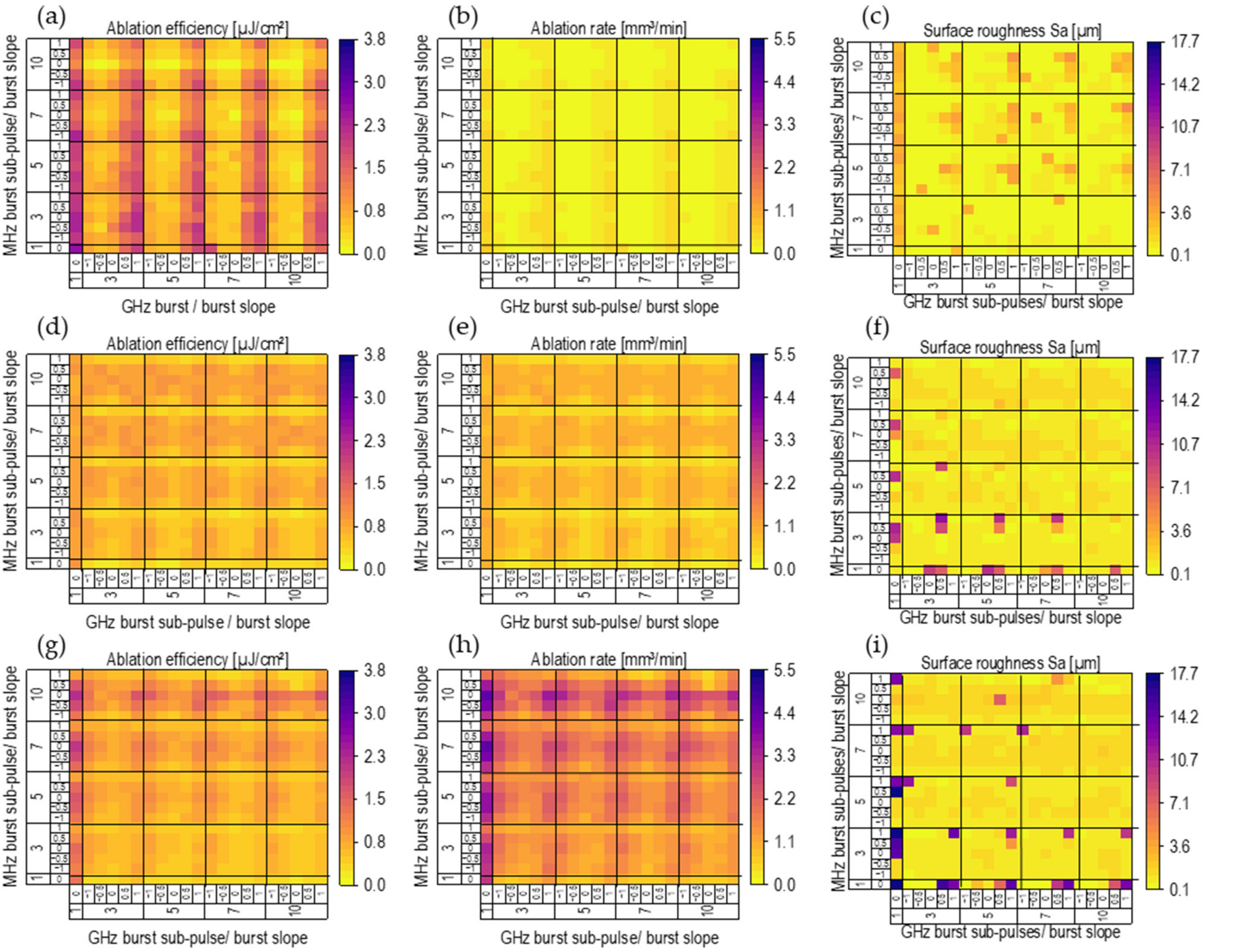

Figure S2. Heat maps of ablation efficiency (**a**, **d**, **g**), ablation rate (**b**, **e**, **h**), and surface roughness Sa (c, f, i) for stainless steel AISI 316Ti under various burst parameters at three fluence levels: top row, 1.7 J·cm^{-2}; middle row, 8.5 J·cm^{-2}; bottom row, 17.0 J·cm^{-2}.

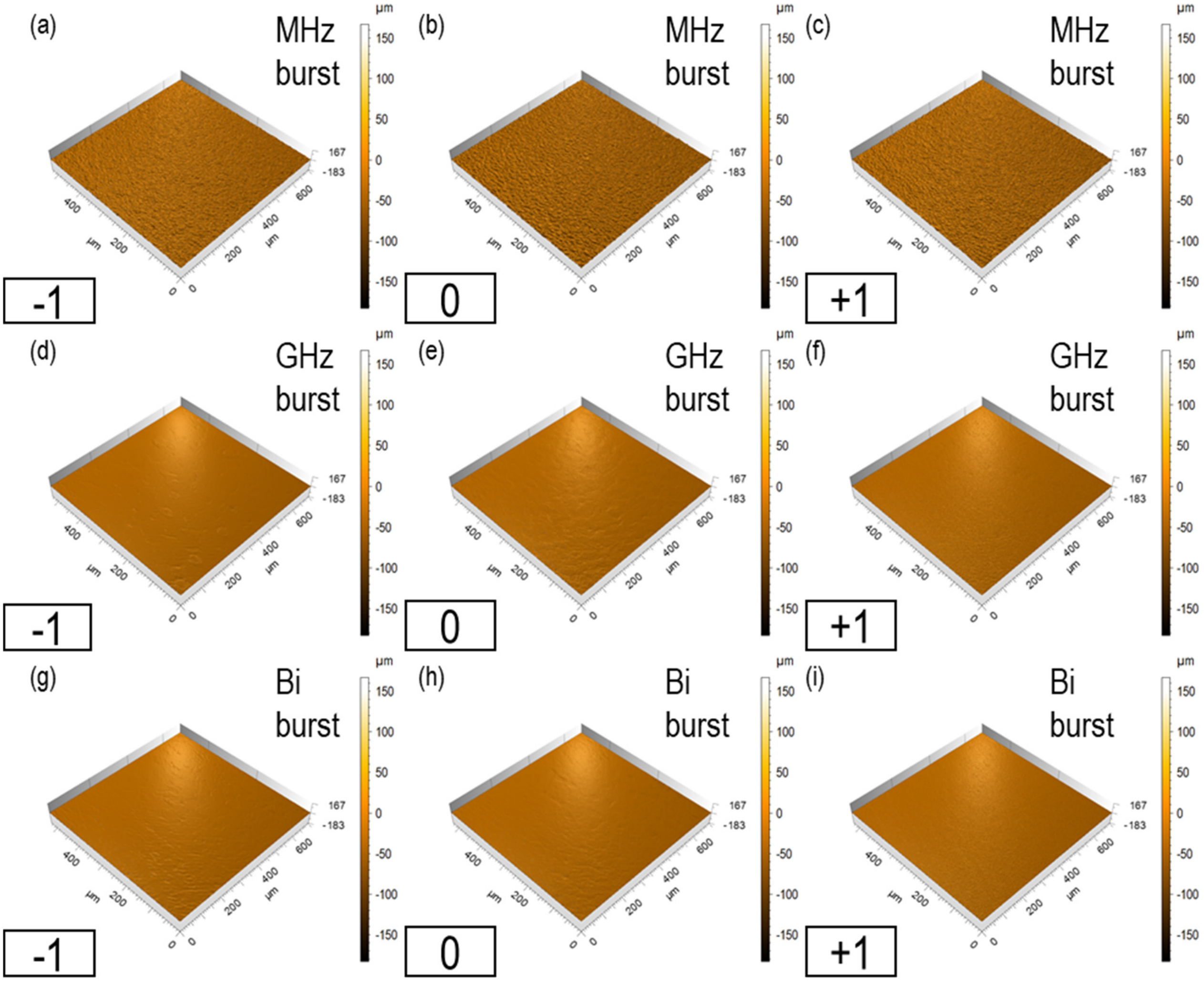

Figure S3. Confocal images of laser-processed AISI 420 surfaces obtained using MHz (**a**–**c**), GHz (**d**–**f**), and Bi-burst (**g**–**i**) modes with burst slopes of −1, 0, and +1 at a fluence of 1.7 J·cm^{-2}.

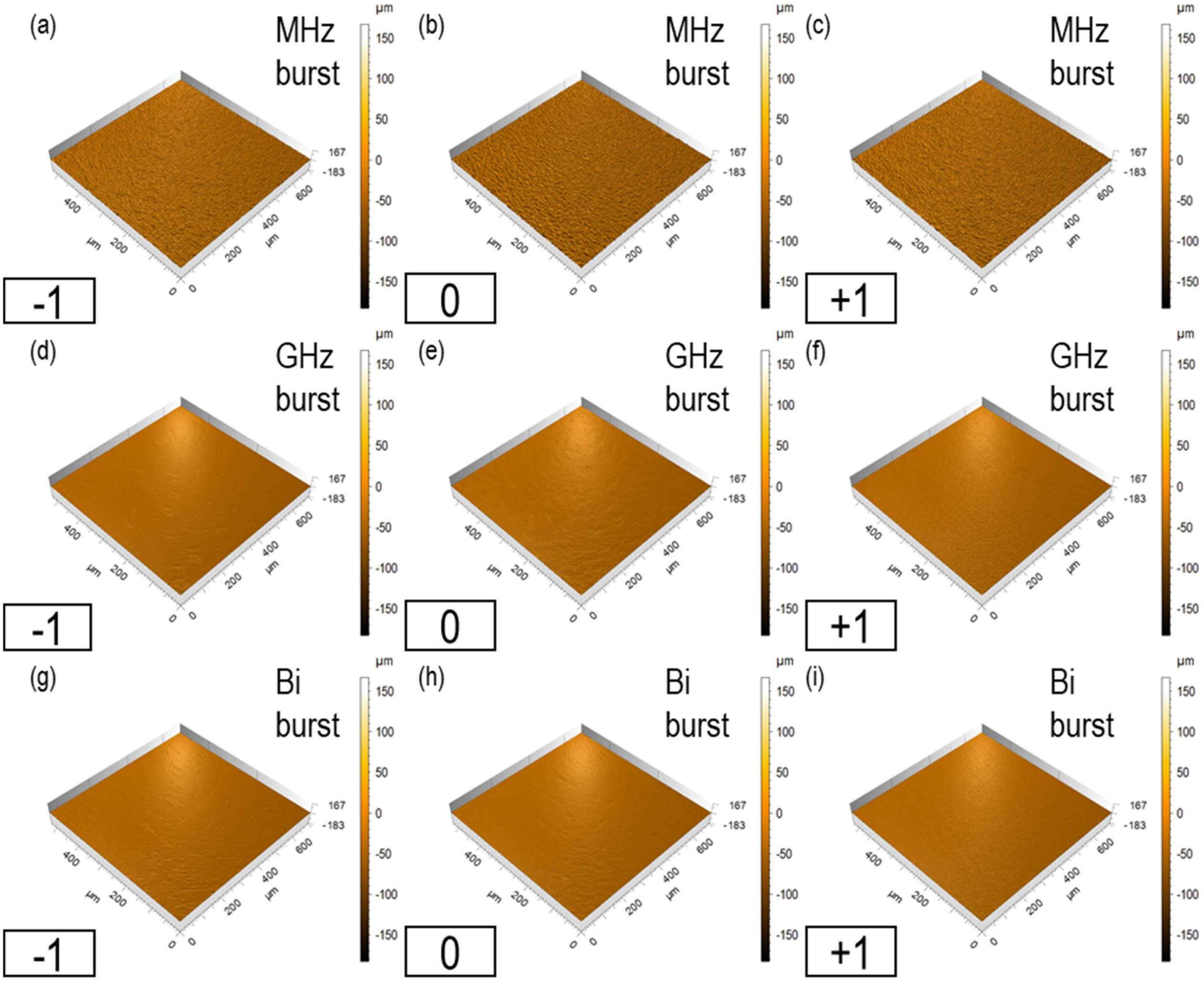

Figure S4. Confocal images of laser-processed AISI 316Ti surfaces obtained using MHz (**a**–**c**), GHz (**d**–**f**), and Bi-burst (**g**–**i**) modes with burst slopes of −1, 0, and +1 at a fluence of 1.7 J·cm⁻².

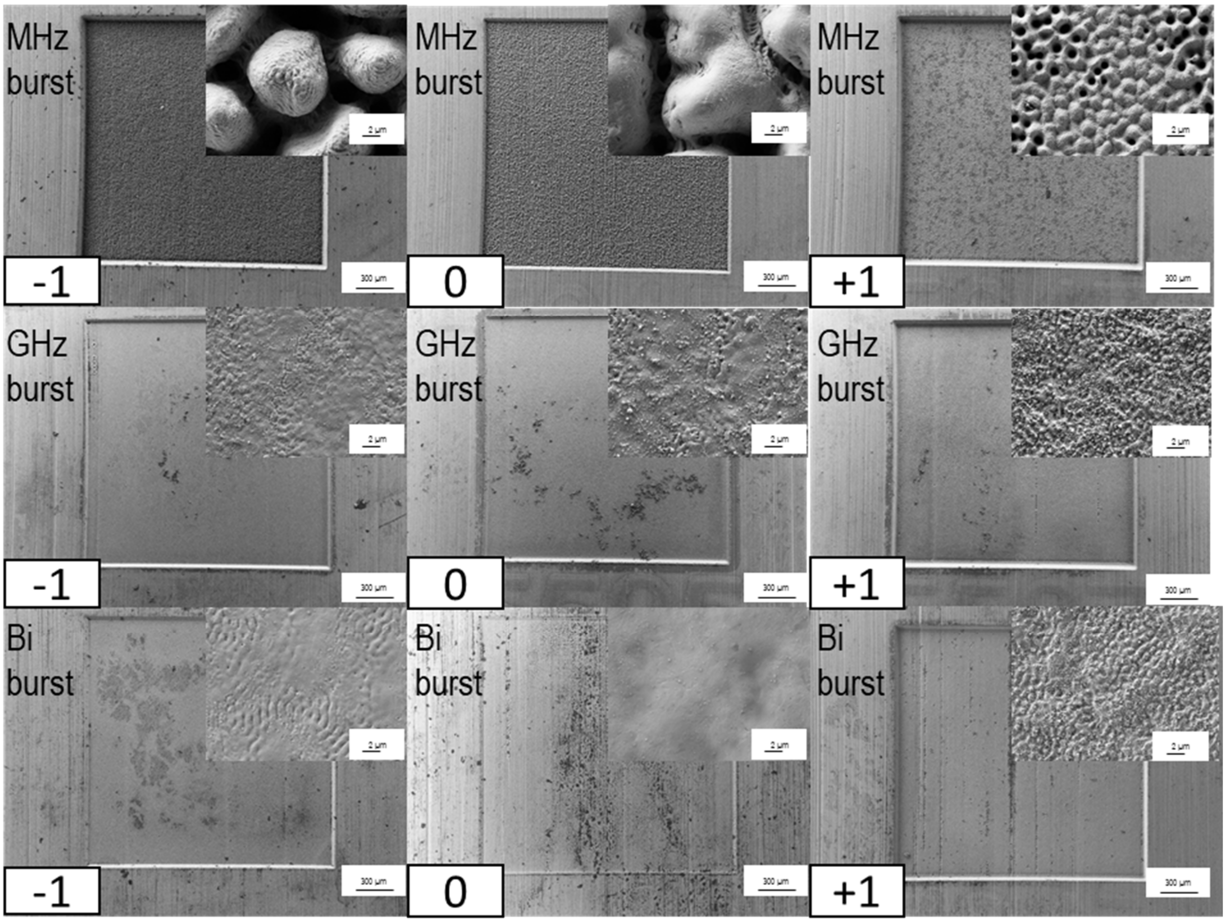

Figure S5. SEM images of cavities produced on AISI 420 steel using MHz, GHz, and Bi-burst modes. The burst slope, corresponding to the temporal energy distribution within the burst, is arranged in columns and varied between −1, 0, and +1, as indicated at the top of the figure. The experiments were performed using 10 sub-pulse MHz and 10 sub-pulse GHz bursts at a constant fluence of approximately 1.7 J·cm^{-2}.

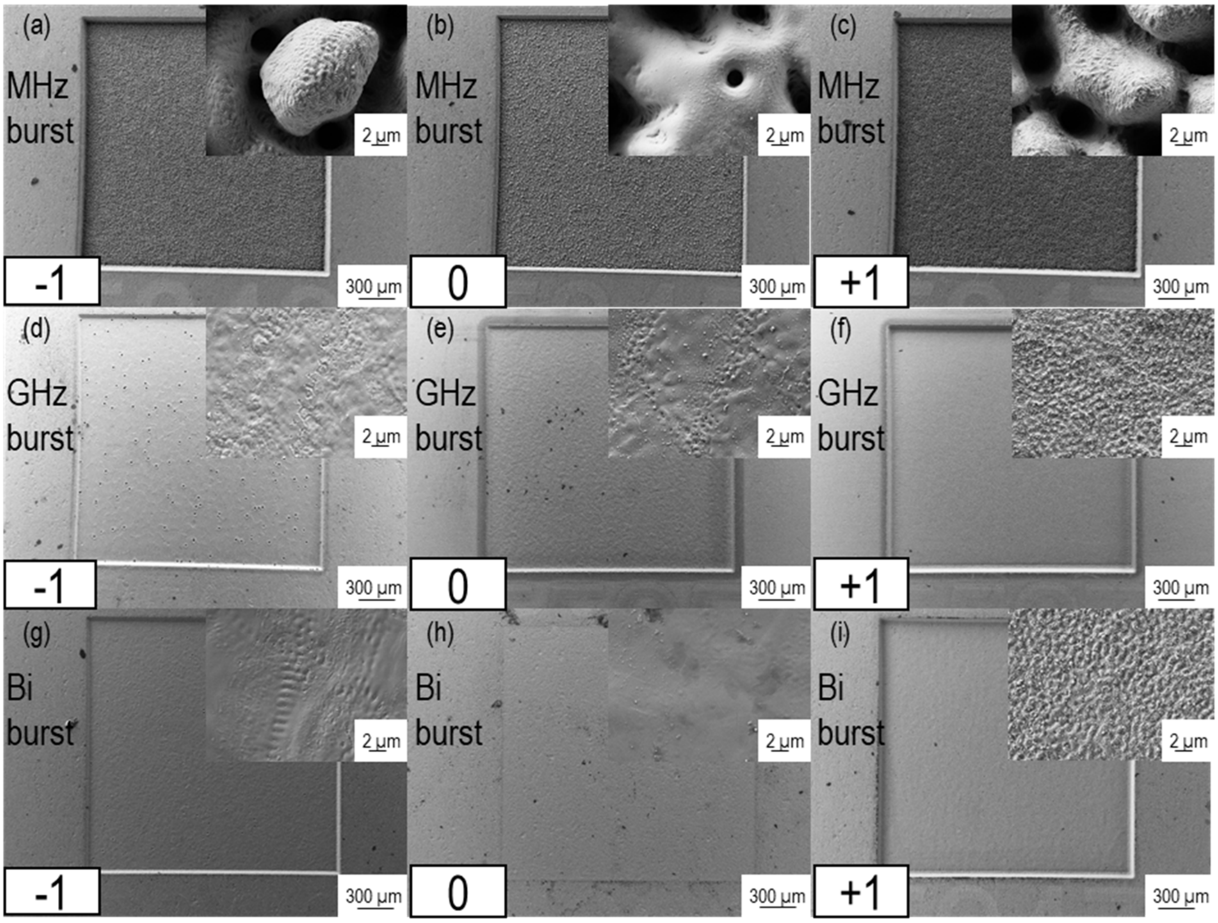

Figure S6. SEM images of cavities produced on AISI 316Ti steel using MHz, GHz, and Bi-burst modes. The burst slope, corresponding to the temporal energy distribution within the burst, is arranged in columns and varied between −1, 0, and +1, as indicated at the top of the figure. The experiments were performed using 10 sub-pulse MHz and 10 sub-pulse GHz bursts at a constant fluence of approximately 1.7 $J{\cdot}cm^{-2}$.

Table S1. Full parameter matrix and resulting ablation efficiency, ablation rate, surface roughness (Sa), and cost function values for all investigated fluence, burst, and slope combinations.

Cost function	Sa [µm]	Ablation rate [$mm^3{\cdot}min^{-1}$]	Ablation eff. [$\mu m^3{\cdot}\mu J^{-1}$]	Speed [mm/s]	MHz burst	GHz burst	MHz burst Slope	GHz burst Slope	Pulse Width [fs]	Frequency [kHz]	Fluence [$J{\cdot}cm^{-2}$]
1.83	1.61	4.53	1.89	3000.00	7.00	1.00	-0.50	0.00	258.00	200.00	17.0
2.06	1.55	4.33	1.80	3000.00	10.00	1.00	0.50	0.00	258.00	200.00	17.0
2.16	1.62	4.25	1.77	3000.00	5.00	1.00	0.00	0.00	258.00	200.00	17.0
2.38	1.69	4.07	1.70	3000.00	5.00	1.00	-0.50	0.00	258.00	200.00	17.0
2.57	1.43	3.89	1.62	3000.00	10.00	10.00	0.00	-1.00	258.00	200.00	17.0
2.57	1.54	3.89	1.62	3000.00	7.00	1.00	0.50	0.00	258.00	200.00	17.0
2.61	1.65	3.87	1.61	3000.00	10.00	5.00	0.00	-1.00	258.00	200.00	17.0
2.61	2.02	3.91	1.63	3000.00	10.00	3.00	0.00	-1.00	258.00	200.00	17.0

Cost function	Sa [μm]	Ablation rate $[mm^3 \cdot min^{-1}]$	Ablation eff. $[μm^3 \cdot μJ^{-1}]$	Speed [mm/s]	MHz burst	GHz burst	MHz burst Slope	GHz burst Slope	Pulse Width [fs]	Fre-quency [kHz]	Fluence $[J \cdot cm^{-2}]$
2.70	2.18	3.85	1.60	3000.00	10.00	7.00	0.00	-1.00	258.00	200.00	17.0
2.72	1.39	3.76	1.56	3000.00	10.00	3.00	0.00	1.00	258.00	200.00	17.0
3.06	2.04	3.53	1.47	3000.00	10.00	5.00	0.00	1.00	258.00	200.00	17.0
3.15	1.30	3.38	1.41	3000.00	10.00	7.00	0.00	1.00	258.00	200.00	17.0
3.30	1.26	3.25	1.35	3000.00	5.00	1.00	-1.00	0.00	258.00	200.00	17.0
3.31	1.66	3.28	1.36	3000.00	10.00	5.00	0.00	-0.50	258.00	200.00	17.0
3.34	1.22	3.21	1.34	3000.00	7.00	1.00	-1.00	0.00	258.00	200.00	17.0
3.36	1.27	3.20	1.33	3000.00	10.00	1.00	-1.00	0.00	258.00	200.00	17.0
3.36	1.56	3.22	1.34	3000.00	3.00	1.00	-0.50	0.00	258.00	200.00	17.0
3.37	1.45	3.21	1.34	3000.00	10.00	10.00	0.00	-0.50	258.00	200.00	17.0
3.39	1.67	3.21	1.34	3000.00	7.00	3.00	0.00	-1.00	258.00	200.00	17.0
3.42	1.59	3.18	1.32	3000.00	7.00	5.00	0.00	-1.00	258.00	200.00	17.0
3.45	1.29	3.13	1.30	3000.00	10.00	10.00	0.00	1.00	258.00	200.00	17.0
3.46	1.71	3.16	1.32	3000.00	7.00	7.00	0.00	-1.00	258.00	200.00	17.0
3.47	1.48	3.13	1.30	3000.00	10.00	3.00	-0.50	1.00	258.00	200.00	17.0
3.48	2.00	3.17	1.32	3000.00	10.00	3.00	0.00	-0.50	258.00	200.00	17.0
3.53	2.34	3.16	1.32	3000.00	10.00	7.00	0.00	-0.50	258.00	200.00	17.0
3.54	1.36	3.06	1.27	3000.00	10.00	3.00	0.50	-1.00	258.00	200.00	17.0
3.61	1.38	3.00	1.25	3000.00	3.00	1.00	-1.00	0.00	258.00	200.00	17.0
3.61	1.41	3.00	1.25	3000.00	7.00	3.00	0.00	1.00	258.00	200.00	17.0
3.64	1.49	2.98	1.24	3000.00	10.00	10.00	0.00	0.00	258.00	200.00	17.0
3.68	0.93	2.89	1.20	3000.00	10.00	10.00	0.50	-1.00	258.00	200.00	17.0
3.74	1.64	2.91	1.21	3000.00	7.00	3.00	-0.50	1.00	258.00	200.00	17.0
3.75	1.55	2.90	1.21	3000.00	10.00	5.00	-0.50	-1.00	258.00	200.00	17.0
3.75	1.45	2.88	1.20	3000.00	10.00	5.00	0.50	-1.00	258.00	200.00	17.0
3.77	1.32	2.86	1.19	3000.00	10.00	7.00	0.50	-1.00	258.00	200.00	17.0
3.77	1.28	2.85	1.19	3000.00	7.00	10.00	0.00	-1.00	258.00	200.00	17.0
3.80	1.59	2.86	1.19	3000.00	10.00	3.00	-0.50	-1.00	258.00	200.00	17.0
3.86	1.37	2.78	1.16	3000.00	7.00	7.00	-0.50	-1.00	258.00	200.00	17.0
3.87	1.63	2.80	1.17	3000.00	7.00	10.00	-0.50	-1.00	258.00	200.00	17.0
3.87	1.39	2.77	1.16	3000.00	10.00	5.00	-0.50	1.00	258.00	200.00	17.0
3.89	1.23	2.74	1.14	3000.00	7.00	3.00	0.50	-1.00	258.00	200.00	17.0

Cost function	Sa [μm]	Ablation rate [$mm^3 \cdot min^{-1}$]	Ablation eff. [$\mu m^3 \cdot \mu J^{-1}$]	Speed [mm/s]	MHz burst	GHz burst	MHz burst Slope	GHz burst Slope	Pulse Width [fs]	Fre-quency [kHz]	Fluence [$J \cdot cm^{-2}$]
3.89	1.53	2.77	1.15	3000.00	7.00	3.00	-0.50	-1.00	258.00	200.00	17.0
3.92	1.34	2.73	1.14	3000.00	5.00	3.00	0.00	-1.00	258.00	200.00	17.0
3.93	1.38	2.72	1.13	3000.00	5.00	10.00	0.00	-1.00	258.00	200.00	17.0
3.94	1.55	2.74	1.14	3000.00	7.00	5.00	0.00	1.00	258.00	200.00	17.0
3.94	1.25	2.70	1.13	3000.00	5.00	7.00	0.00	-1.00	258.00	200.00	17.0
3.97	1.25	2.68	1.11	3000.00	7.00	5.00	0.50	-1.00	258.00	200.00	17.0
3.97	1.43	2.69	1.12	3000.00	7.00	5.00	-0.50	-1.00	258.00	200.00	17.0
3.97	1.34	2.68	1.12	3000.00	5.00	5.00	0.00	-1.00	258.00	200.00	17.0
3.98	1.50	2.69	1.12	3000.00	7.00	5.00	-0.50	1.00	258.00	200.00	17.0
3.99	1.41	2.67	1.11	3000.00	10.00	7.00	-0.50	-1.00	258.00	200.00	17.0
3.99	1.31	2.66	1.11	3000.00	7.00	10.00	0.50	-1.00	258.00	200.00	17.0
4.03	1.54	2.65	1.11	3000.00	10.00	7.00	-0.50	1.00	258.00	200.00	17.0
4.04	1.23	2.62	1.09	3000.00	7.00	7.00	0.50	-1.00	258.00	200.00	17.0
4.05	1.29	2.61	1.09	3000.00	10.00	3.00	0.50	1.00	258.00	200.00	17.0
4.07	1.69	2.64	1.10	3000.00	10.00	10.00	-0.50	-1.00	258.00	200.00	17.0
4.13	1.47	2.56	1.07	3000.00	10.00	10.00	-0.50	1.00	258.00	200.00	17.0
4.13	0.81	2.50	1.04	3000.00	10.00	10.00	0.50	1.00	258.00	200.00	17.0
4.13	1.54	2.57	1.07	3000.00	7.00	7.00	0.00	1.00	258.00	200.00	17.0
4.15	1.18	2.51	1.05	3000.00	10.00	7.00	0.50	1.00	258.00	200.00	17.0
4.21	1.56	2.51	1.04	3000.00	7.00	7.00	-0.50	1.00	258.00	200.00	17.0
4.23	1.41	2.48	1.03	3000.00	10.00	3.00	0.00	0.50	258.00	200.00	17.0
4.23	1.16	2.45	1.02	3000.00	5.00	3.00	0.50	-1.00	258.00	200.00	17.0
4.23	1.59	2.49	1.04	3000.00	7.00	3.00	0.00	-0.50	258.00	200.00	17.0
4.23	1.26	2.46	1.02	3000.00	5.00	3.00	0.00	1.00	258.00	200.00	17.0
4.23	1.32	2.46	1.02	3000.00	10.00	5.00	0.50	1.00	258.00	200.00	17.0
4.24	1.44	2.47	1.03	3000.00	10.00	3.00	0.50	-0.50	258.00	200.00	17.0
4.25	1.72	2.49	1.04	3000.00	7.00	5.00	0.00	-0.50	258.00	200.00	17.0
4.25	1.41	2.45	1.02	3000.00	7.00	10.00	0.00	1.00	258.00	200.00	17.0
4.26	0.82	2.39	1.00	3000.00	10.00	10.00	0.50	-0.50	258.00	200.00	17.0
4.27	1.88	2.49	1.04	3000.00	10.00	5.00	0.00	0.00	258.00	200.00	17.0
4.27	1.50	2.44	1.02	3000.00	10.00	10.00	0.00	0.50	258.00	200.00	17.0
4.28	1.43	2.43	1.01	3000.00	5.00	3.00	-0.50	1.00	258.00	200.00	17.0

Cost function	Sa [μm]	Ablation rate [mm³·min⁻¹]	Ablation eff. [μm³·μJ⁻¹]	Speed [mm/s]	MHz burst	GHz burst	MHz burst Slope	GHz burst Slope	Pulse Width [fs]	Fre-quency [kHz]	Fluence [J·cm⁻²]
4.31	1.76	2.44	1.02	3000.00	7.00	7.00	0.00	-0.50	258.00	200.00	17.0
4.31	1.22	2.38	0.99	3000.00	5.00	5.00	0.50	-1.00	258.00	200.00	17.0
4.33	1.14	2.36	0.98	3000.00	5.00	5.00	-0.50	-1.00	258.00	200.00	17.0
4.33	1.79	2.43	1.01	3000.00	10.00	10.00	-0.50	-0.50	258.00	200.00	17.0
4.33	1.21	2.37	0.99	3000.00	5.00	3.00	-0.50	-1.00	258.00	200.00	17.0
4.34	1.81	2.42	1.01	3000.00	7.00	10.00	0.00	-0.50	258.00	200.00	17.0
4.34	1.17	2.35	0.98	3000.00	5.00	7.00	0.50	-1.00	258.00	200.00	17.0
4.35	1.49	2.37	0.99	3000.00	10.00	7.00	0.50	-0.50	258.00	200.00	17.0
4.38	1.45	2.35	0.98	3000.00	10.00	7.00	-0.50	-0.50	258.00	200.00	17.0
4.38	1.55	2.36	0.98	3000.00	10.00	5.00	0.50	-0.50	258.00	200.00	17.0
4.39	1.06	2.30	0.96	3000.00	5.00	3.00	-1.00	1.00	258.00	200.00	17.0
4.41	1.77	2.36	0.98	3000.00	7.00	10.00	-0.50	1.00	258.00	200.00	17.0
4.41	1.43	2.32	0.97	3000.00	10.00	7.00	0.00	0.50	258.00	200.00	17.0
4.42	1.03	2.27	0.95	3000.00	7.00	3.00	-1.00	1.00	258.00	200.00	17.0
4.46	0.96	2.23	0.93	3000.00	7.00	3.00	0.50	1.00	258.00	200.00	17.0
4.48	1.65	2.28	0.95	3000.00	10.00	3.00	-0.50	-0.50	258.00	200.00	17.0
4.48	1.10	2.22	0.93	3000.00	5.00	5.00	0.00	1.00	258.00	200.00	17.0
4.49	1.31	2.24	0.93	3000.00	10.00	3.00	-1.00	1.00	258.00	200.00	17.0
4.50	1.60	2.26	0.94	3000.00	10.00	5.00	-0.50	-0.50	258.00	200.00	17.0
4.50	0.62	2.16	0.90	3000.00	10.00	10.00	0.50	0.50	258.00	200.00	17.0
4.51	1.21	2.21	0.92	3000.00	5.00	7.00	-0.50	-1.00	258.00	200.00	17.0
4.51	1.81	2.27	0.95	3000.00	7.00	10.00	-0.50	-0.50	258.00	200.00	17.0
4.53	1.28	2.20	0.92	3000.00	10.00	3.00	-0.50	0.50	258.00	200.00	17.0
4.54	1.06	2.17	0.90	3000.00	5.00	10.00	0.50	-1.00	258.00	200.00	17.0
4.54	1.43	2.21	0.92	3000.00	7.00	3.00	0.50	-0.50	258.00	200.00	17.0
4.55	1.57	2.21	0.92	3000.00	7.00	7.00	-0.50	-0.50	258.00	200.00	17.0
4.58	1.42	2.17	0.91	3000.00	5.00	10.00	-0.50	-1.00	258.00	200.00	17.0
4.59	0.58	2.09	0.87	3000.00	10.00	10.00	0.50	0.00	258.00	200.00	17.0
4.59	1.98	2.22	0.93	3000.00	7.00	10.00	0.00	0.00	258.00	200.00	17.0
4.60	1.36	2.15	0.90	3000.00	5.00	5.00	-0.50	1.00	258.00	200.00	17.0
4.61	1.65	2.17	0.91	3000.00	7.00	3.00	-0.50	-0.50	258.00	200.00	17.0
4.61	1.12	2.12	0.88	3000.00	5.00	7.00	0.00	1.00	258.00	200.00	17.0

Cost function	Sa [μm]	Ablation rate [mm³·min⁻¹]	Ablation eff. [μm³·μJ⁻¹]	Speed [mm/s]	MHz burst	GHz burst	MHz burst Slope	GHz burst Slope	Pulse Width [fs]	Fre-quency [kHz]	Fluence [J·cm⁻²]
4.62	1.21	2.12	0.88	3000.00	7.00	5.00	0.50	1.00	258.00	200.00	17.0
4.62	1.36	2.13	0.89	3000.00	7.00	7.00	0.50	1.00	258.00	200.00	17.0
4.63	1.35	2.12	0.88	3000.00	7.00	10.00	0.50	1.00	258.00	200.00	17.0
4.64	1.04	2.09	0.87	3000.00	5.00	5.00	-1.00	1.00	258.00	200.00	17.0
4.64	1.58	2.14	0.89	3000.00	7.00	5.00	-0.50	-0.50	258.00	200.00	17.0
4.66	1.25	2.09	0.87	3000.00	10.00	3.00	0.50	0.50	258.00	200.00	17.0
4.66	1.36	2.10	0.88	3000.00	7.00	5.00	0.50	-0.50	258.00	200.00	17.0
4.67	1.16	2.07	0.86	3000.00	10.00	3.00	1.00	-1.00	258.00	200.00	17.0
4.68	1.38	2.09	0.87	3000.00	5.00	3.00	0.00	-0.50	258.00	200.00	17.0
4.68	1.06	2.05	0.86	3000.00	3.00	3.00	0.00	-1.00	258.00	200.00	17.0
4.69	1.13	2.05	0.85	3000.00	7.00	5.00	-1.00	1.00	258.00	200.00	17.0
4.70	0.64	2.00	0.83	3000.00	10.00	7.00	1.00	-1.00	258.00	200.00	17.0
4.70	1.51	2.08	0.87	3000.00	7.00	10.00	0.50	-0.50	258.00	200.00	17.0
4.71	1.58	2.08	0.87	3000.00	10.00	5.00	0.50	0.00	258.00	200.00	17.0
4.71	1.94	2.11	0.88	3000.00	10.00	7.00	0.00	0.00	258.00	200.00	17.0
4.72	1.36	2.05	0.85	3000.00	5.00	7.00	-0.50	1.00	258.00	200.00	17.0
4.73	1.55	2.06	0.86	3000.00	10.00	3.00	0.50	0.00	258.00	200.00	17.0
4.74	1.38	2.04	0.85	3000.00	10.00	5.00	-0.50	0.50	258.00	200.00	17.0
4.74	1.88	2.08	0.87	3000.00	7.00	7.00	0.50	-0.50	258.00	200.00	17.0
4.75	1.12	2.00	0.83	3000.00	3.00	10.00	0.00	-1.00	258.00	200.00	17.0
4.75	0.92	1.98	0.83	3000.00	5.00	3.00	0.50	1.00	258.00	200.00	17.0
4.75	1.21	2.01	0.84	3000.00	10.00	5.00	-1.00	1.00	258.00	200.00	17.0
4.75	0.98	1.98	0.83	3000.00	3.00	3.00	0.50	-1.00	258.00	200.00	17.0
4.75	1.43	2.03	0.84	3000.00	10.00	10.00	-0.50	0.00	258.00	200.00	17.0
4.77	1.25	2.00	0.83	3000.00	10.00	5.00	1.00	-1.00	258.00	200.00	17.0
4.77	1.42	2.01	0.84	3000.00	5.00	10.00	0.00	-0.50	258.00	200.00	17.0
4.77	1.16	1.99	0.83	3000.00	7.00	3.00	0.00	0.50	258.00	200.00	17.0
4.79	1.29	1.99	0.83	3000.00	7.00	3.00	-0.50	0.50	258.00	200.00	17.0
4.79	0.62	1.92	0.80	3000.00	10.00	10.00	-1.00	-1.00	258.00	200.00	17.0
4.80	1.40	1.99	0.83	3000.00	5.00	5.00	0.00	-0.50	258.00	200.00	17.0
4.80	0.89	1.54	1.28	3000.00	10.00	1.00	-1.00	0.00	258.00	200.00	8.5
4.81	1.05	1.95	0.81	3000.00	5.00	7.00	-1.00	1.00	258.00	200.00	17.0

Cost function	Sa [μm]	Ablation rate [mm³·min⁻¹]	Ablation eff. [μm³·μJ⁻¹]	Speed [mm/s]	MHz burst	GHz burst	MHz burst Slope	GHz burst Slope	Pulse Width [fs]	Fre-quency [kHz]	Fluence [J·cm⁻²]
4.81	1.15	1.95	0.81	3000.00	7.00	3.00	0.50	0.50	258.00	200.00	17.0
4.82	0.80	1.52	1.27	3000.00	5.00	1.00	-1.00	0.00	258.00	200.00	8.5
4.82	1.94	2.02	0.84	3000.00	10.00	7.00	0.50	0.00	258.00	200.00	17.0
4.82	1.71	2.00	0.83	3000.00	7.00	7.00	0.00	0.00	258.00	200.00	17.0
4.82	1.24	1.95	0.81	3000.00	5.00	3.00	0.50	-0.50	258.00	200.00	17.0
4.82	1.81	2.01	0.84	3000.00	10.00	7.00	-0.50	0.50	258.00	200.00	17.0
4.82	1.32	1.96	0.82	3000.00	10.00	7.00	0.50	0.50	258.00	200.00	17.0
4.83	0.89	1.52	1.27	3000.00	3.00	1.00	-1.00	0.00	258.00	200.00	8.5
4.83	1.45	1.97	0.82	3000.00	5.00	7.00	0.00	-0.50	258.00	200.00	17.0
4.83	1.32	1.95	0.81	3000.00	5.00	10.00	-0.50	1.00	258.00	200.00	17.0
4.83	1.08	1.93	0.80	3000.00	5.00	10.00	-1.00	-1.00	258.00	200.00	17.0
4.84	0.84	1.51	1.26	3000.00	7.00	5.00	0.00	1.00	258.00	200.00	8.5
4.84	1.10	1.92	0.80	3000.00	7.00	7.00	-1.00	1.00	258.00	200.00	17.0
4.85	0.44	1.85	0.77	3000.00	10.00	10.00	1.00	-1.00	258.00	200.00	17.0
4.85	0.92	1.51	1.26	3000.00	7.00	1.00	-1.00	0.00	258.00	200.00	8.5
4.85	1.32	1.93	0.81	3000.00	7.00	10.00	-1.00	-1.00	258.00	200.00	17.0
4.86	1.14	1.52	1.27	3000.00	10.00	3.00	0.50	-1.00	258.00	200.00	8.5
4.86	1.11	1.51	1.26	3000.00	5.00	3.00	0.00	-1.00	258.00	200.00	8.5
4.86	0.95	1.89	0.79	3000.00	3.00	5.00	0.50	-1.00	258.00	200.00	17.0
4.87	1.09	1.90	0.79	3000.00	5.00	10.00	0.00	1.00	258.00	200.00	17.0
4.87	1.45	1.93	0.81	3000.00	7.00	3.00	0.50	0.00	258.00	200.00	17.0
4.87	0.85	1.49	1.24	3000.00	10.00	1.00	0.50	0.00	258.00	200.00	8.5
4.87	0.78	1.87	0.78	3000.00	5.00	5.00	0.50	1.00	258.00	200.00	17.0
4.87	1.18	1.51	1.26	3000.00	7.00	7.00	0.00	-1.00	258.00	200.00	8.5
4.87	0.96	1.88	0.78	3000.00	5.00	10.00	-1.00	1.00	258.00	200.00	17.0
4.87	0.87	1.87	0.78	3000.00	10.00	7.00	-1.00	1.00	258.00	200.00	17.0
4.87	0.82	1.87	0.78	3000.00	7.00	3.00	-1.00	-1.00	258.00	200.00	17.0
4.88	1.53	1.93	0.80	3000.00	10.00	3.00	0.00	0.00	258.00	200.00	17.0
4.88	0.92	1.87	0.78	3000.00	5.00	3.00	-1.00	-1.00	258.00	200.00	17.0
4.88	1.13	1.50	1.25	3000.00	5.00	7.00	0.00	-1.00	258.00	200.00	8.5
4.88	0.93	1.87	0.78	3000.00	10.00	7.00	-1.00	-1.00	258.00	200.00	17.0
4.88	1.08	1.49	1.25	3000.00	5.00	3.00	-0.50	1.00	258.00	200.00	8.5

Cost function	Sa [μm]	Ablation rate [mm³·min⁻¹]	Ablation eff. [μm³·μJ⁻¹]	Speed [mm/s]	MHz burst	GHz burst	MHz burst Slope	GHz burst Slope	Pulse Width [fs]	Fre-quency [kHz]	Fluence [J·cm⁻²]
4.89	1.25	1.51	1.26	3000.00	10.00	3.00	0.00	0.50	258.00	200.00	8.5
4.89	0.87	1.48	1.23	3000.00	7.00	7.00	0.00	1.00	258.00	200.00	8.5
4.89	1.00	1.87	0.78	3000.00	5.00	7.00	-1.00	-1.00	258.00	200.00	17.0
4.89	1.30	1.51	1.26	3000.00	7.00	3.00	0.00	-1.00	258.00	200.00	8.5
4.89	0.89	1.86	0.77	3000.00	5.00	7.00	0.50	1.00	258.00	200.00	17.0
4.89	1.76	1.54	1.29	3000.00	10.00	3.00	-0.50	1.00	258.00	200.00	8.5
4.89	1.23	1.50	1.25	3000.00	7.00	10.00	0.00	-1.00	258.00	200.00	8.5
4.89	0.94	1.48	1.23	3000.00	7.00	10.00	0.00	1.00	258.00	200.00	8.5
4.90	1.15	1.88	0.78	3000.00	7.00	7.00	-1.00	-1.00	258.00	200.00	17.0
4.90	1.50	1.52	1.27	3000.00	10.00	10.00	0.00	-0.50	258.00	200.00	8.5
4.90	0.97	1.48	1.23	3000.00	10.00	7.00	0.50	1.00	258.00	200.00	8.5
4.90	1.66	1.53	1.28	3000.00	10.00	5.00	-0.50	1.00	258.00	200.00	8.5
4.90	1.15	1.49	1.24	3000.00	10.00	7.00	0.50	-1.00	258.00	200.00	8.5
4.90	1.08	1.48	1.24	3000.00	5.00	5.00	0.00	-1.00	258.00	200.00	8.5
4.90	1.01	1.48	1.23	3000.00	10.00	3.00	0.00	1.00	258.00	200.00	8.5
4.90	1.08	1.87	0.78	3000.00	7.00	10.00	-1.00	1.00	258.00	200.00	17.0
4.91	1.43	1.90	0.79	3000.00	10.00	5.00	0.50	0.50	258.00	200.00	17.0
4.91	1.48	1.90	0.79	3000.00	7.00	3.00	0.00	0.00	258.00	200.00	17.0
4.91	1.01	1.85	0.77	3000.00	5.00	10.00	0.50	1.00	258.00	200.00	17.0
4.91	1.51	1.51	1.26	3000.00	10.00	5.00	0.00	-0.50	258.00	200.00	8.5
4.91	1.13	1.48	1.24	3000.00	5.00	10.00	0.00	-1.00	258.00	200.00	8.5
4.91	0.68	1.45	1.21	3000.00	5.00	3.00	0.00	1.00	258.00	200.00	8.5
4.91	0.96	1.85	0.77	3000.00	3.00	7.00	0.50	-1.00	258.00	200.00	17.0
4.91	0.95	1.85	0.77	3000.00	7.00	5.00	-1.00	-1.00	258.00	200.00	17.0
4.91	1.48	1.90	0.79	3000.00	7.00	5.00	0.50	0.00	258.00	200.00	17.0
4.91	0.93	1.46	1.22	3000.00	7.00	3.00	0.00	1.00	258.00	200.00	8.5
4.91	1.54	1.51	1.26	3000.00	7.00	5.00	-0.50	1.00	258.00	200.00	8.5
4.91	0.96	1.85	0.77	3000.00	5.00	5.00	-1.00	-1.00	258.00	200.00	17.0
4.91	0.83	1.83	0.76	3000.00	3.00	3.00	-0.50	-1.00	258.00	200.00	17.0
4.91	1.40	1.50	1.25	3000.00	10.00	5.00	0.00	0.50	258.00	200.00	8.5
4.91	1.21	1.87	0.78	3000.00	3.00	5.00	0.00	-1.00	258.00	200.00	17.0
4.91	1.25	1.49	1.24	3000.00	5.00	5.00	-0.50	1.00	258.00	200.00	8.5

Cost function	Sa [µm]	Ablation rate [mm³·min⁻¹]	Ablation eff. [µm³·µJ⁻¹]	Speed [mm/s]	MHz burst	GHz burst	MHz burst Slope	GHz burst Slope	Pulse Width [fs]	Fre-quency [kHz]	Fluence [J·cm⁻²]
4.92	1.34	1.49	1.25	3000.00	10.00	10.00	0.00	1.00	258.00	200.00	8.5
4.92	1.27	1.49	1.24	3000.00	7.00	5.00	0.00	-1.00	258.00	200.00	8.5
4.92	1.50	1.51	1.26	3000.00	10.00	3.00	0.00	-0.50	258.00	200.00	8.5
4.92	0.75	1.45	1.21	3000.00	5.00	5.00	0.00	1.00	258.00	200.00	8.5
4.92	1.15	1.48	1.23	3000.00	10.00	5.00	0.50	-1.00	258.00	200.00	8.5
4.92	1.08	1.85	0.77	3000.00	10.00	3.00	-1.00	-1.00	258.00	200.00	17.0
4.92	1.59	1.90	0.79	3000.00	7.00	5.00	0.00	0.00	258.00	200.00	17.0
4.93	0.91	1.45	1.21	3000.00	5.00	1.00	0.00	0.00	258.00	200.00	8.5
4.93	1.48	1.88	0.78	3000.00	10.00	10.00	-0.50	0.50	258.00	200.00	17.0
4.93	1.35	1.87	0.78	3000.00	5.00	5.00	0.50	-0.50	258.00	200.00	17.0
4.93	1.65	1.51	1.26	3000.00	7.00	10.00	-0.50	1.00	258.00	200.00	8.5
4.93	1.50	1.49	1.25	3000.00	10.00	7.00	0.00	-0.50	258.00	200.00	8.5
4.94	0.98	1.45	1.21	3000.00	5.00	1.00	-0.50	0.00	258.00	200.00	8.5
4.94	1.62	1.50	1.25	3000.00	7.00	7.00	-0.50	1.00	258.00	200.00	8.5
4.94	1.22	1.85	0.77	3000.00	5.00	7.00	0.50	-0.50	258.00	200.00	17.0
4.94	0.89	1.44	1.20	3000.00	7.00	3.00	-1.00	1.00	258.00	200.00	8.5
4.94	1.43	1.48	1.24	3000.00	10.00	3.00	0.00	-1.00	258.00	200.00	8.5
4.95	1.53	1.49	1.24	3000.00	7.00	3.00	-0.50	1.00	258.00	200.00	8.5
4.95	0.97	1.82	0.76	3000.00	3.00	7.00	-0.50	-1.00	258.00	200.00	17.0
4.95	1.51	1.87	0.78	3000.00	5.00	10.00	-0.50	-0.50	258.00	200.00	17.0
4.95	1.40	1.48	1.23	3000.00	10.00	7.00	0.00	-1.00	258.00	200.00	8.5
4.95	1.38	1.86	0.77	3000.00	7.00	7.00	-0.50	0.50	258.00	200.00	17.0
4.95	0.97	1.44	1.20	3000.00	10.00	3.00	-1.00	1.00	258.00	200.00	8.5
4.95	1.39	1.85	0.77	3000.00	5.00	3.00	-0.50	-0.50	258.00	200.00	17.0
4.95	1.37	1.47	1.23	3000.00	7.00	1.00	-0.50	0.00	258.00	200.00	8.5
4.96	1.12	1.83	0.76	3000.00	10.00	5.00	-1.00	-1.00	258.00	200.00	17.0
4.96	1.75	1.50	1.25	3000.00	10.00	7.00	-0.50	1.00	258.00	200.00	8.5
4.96	0.88	1.80	0.75	3000.00	3.00	5.00	-0.50	-1.00	258.00	200.00	17.0
4.96	1.18	1.83	0.76	3000.00	3.00	7.00	0.00	-1.00	258.00	200.00	17.0
4.97	1.23	1.45	1.21	3000.00	7.00	3.00	0.50	-1.00	258.00	200.00	8.5
4.97	1.02	1.81	0.75	3000.00	5.00	3.00	0.50	0.50	258.00	200.00	17.0
4.97	1.66	1.87	0.78	3000.00	7.00	10.00	-0.50	0.50	258.00	200.00	17.0

Cost function	Sa [μm]	Ablation rate [mm³·min⁻¹]	Ablation eff. [μm³·μJ⁻¹]	Speed [mm/s]	MHz burst	GHz burst	MHz burst Slope	GHz burst Slope	Pulse Width [fs]	Fre-quency [kHz]	Fluence [J·cm⁻²]
4.97	1.34	1.46	1.22	3000.00	5.00	10.00	-0.50	-1.00	258.00	200.00	8.5
4.97	0.77	1.41	1.18	3000.00	5.00	7.00	0.00	1.00	258.00	200.00	8.5
4.98	1.61	1.86	0.77	3000.00	7.00	10.00	-0.50	0.00	258.00	200.00	17.0
4.98	1.21	1.82	0.76	3000.00	5.00	10.00	0.50	-0.50	258.00	200.00	17.0
4.98	1.23	1.82	0.76	3000.00	10.00	10.00	-1.00	1.00	258.00	200.00	17.0
4.98	1.45	1.84	0.77	3000.00	7.00	5.00	-0.50	0.50	258.00	200.00	17.0
4.98	0.93	1.79	0.74	3000.00	3.00	3.00	-1.00	1.00	258.00	200.00	17.0
4.98	0.91	1.42	1.18	3000.00	10.00	5.00	0.50	1.00	258.00	200.00	8.5
4.98	7.21	2.41	1.00	3000.00	10.00	5.00	0.00	0.50	258.00	200.00	17.0
4.99	1.21	1.81	0.75	3000.00	7.00	5.00	0.00	0.50	258.00	200.00	17.0
4.99	1.75	1.48	1.23	3000.00	7.00	7.00	-0.50	-1.00	258.00	200.00	8.5
4.99	1.38	1.45	1.21	3000.00	10.00	10.00	0.00	-1.00	258.00	200.00	8.5
4.99	1.38	1.45	1.21	3000.00	10.00	5.00	0.00	-1.00	258.00	200.00	8.5
5.00	1.34	1.81	0.76	3000.00	5.00	7.00	-0.50	-0.50	258.00	200.00	17.0
5.01	1.27	1.43	1.19	3000.00	5.00	7.00	-0.50	1.00	258.00	200.00	8.5
5.01	1.20	1.42	1.19	3000.00	7.00	7.00	0.50	-1.00	258.00	200.00	8.5
5.01	1.24	1.42	1.19	3000.00	10.00	7.00	0.00	1.00	258.00	200.00	8.5
5.01	1.57	1.45	1.21	3000.00	10.00	10.00	0.00	0.00	258.00	200.00	8.5
5.02	1.44	1.43	1.20	3000.00	10.00	7.00	0.00	0.50	258.00	200.00	8.5
5.02	2.01	1.48	1.23	3000.00	10.00	10.00	-0.50	-1.00	258.00	200.00	8.5
5.02	1.28	1.78	0.74	3000.00	5.00	5.00	-0.50	-0.50	258.00	200.00	17.0
5.03	1.60	1.44	1.20	3000.00	10.00	1.00	-0.50	0.00	258.00	200.00	8.5
5.03	1.31	1.78	0.74	3000.00	5.00	3.00	0.50	0.00	258.00	200.00	17.0
5.03	1.04	1.39	1.16	3000.00	7.00	1.00	0.00	0.00	258.00	200.00	8.5
5.03	1.57	1.43	1.20	3000.00	10.00	5.00	0.00	0.00	258.00	200.00	8.5
5.03	1.04	1.39	1.16	3000.00	10.00	10.00	0.50	1.00	258.00	200.00	8.5
5.03	0.82	1.37	1.15	3000.00	10.00	3.00	0.50	1.00	258.00	200.00	8.5
5.03	1.25	1.77	0.74	3000.00	10.00	7.00	-0.50	0.00	258.00	200.00	17.0
5.04	1.42	1.79	0.74	3000.00	7.00	7.00	0.00	0.50	258.00	200.00	17.0
5.04	1.49	1.79	0.75	3000.00	10.00	3.00	-0.50	0.00	258.00	200.00	17.0
5.04	1.19	1.40	1.17	3000.00	10.00	5.00	0.00	1.00	258.00	200.00	8.5
5.04	1.81	1.45	1.21	3000.00	7.00	3.00	-0.50	-1.00	258.00	200.00	8.5

Cost function	Sa [µm]	Ablation rate [mm³·min⁻¹]	Ablation eff. [µm³·µJ⁻¹]	Speed [mm/s]	MHz burst	GHz burst	MHz burst Slope	GHz burst Slope	Pulse Width [fs]	Fre-quency [kHz]	Fluence [J·cm⁻²]
5.04	1.58	1.80	0.75	3000.00	7.00	10.00	0.00	0.50	258.00	200.00	17.0
5.05	1.53	1.42	1.18	3000.00	10.00	10.00	0.00	0.50	258.00	200.00	8.5
5.06	0.70	1.70	0.71	3000.00	3.00	3.00	0.00	1.00	258.00	200.00	17.0
5.06	0.82	1.71	0.71	3000.00	3.00	3.00	-0.50	1.00	258.00	200.00	17.0
5.06	1.28	1.39	1.16	3000.00	7.00	5.00	0.50	-1.00	258.00	200.00	8.5
5.06	1.39	1.76	0.73	3000.00	5.00	5.00	0.50	0.00	258.00	200.00	17.0
5.07	1.79	1.43	1.19	3000.00	10.00	10.00	-0.50	1.00	258.00	200.00	8.5
5.07	0.81	1.35	1.13	3000.00	5.00	10.00	0.00	1.00	258.00	200.00	8.5
5.07	1.45	1.76	0.73	3000.00	7.00	10.00	0.50	0.50	258.00	200.00	17.0
5.08	1.98	1.43	1.20	3000.00	10.00	5.00	-0.50	-1.00	258.00	200.00	8.5
5.08	1.19	1.37	1.14	3000.00	10.00	10.00	0.50	-1.00	258.00	200.00	8.5
5.08	0.89	1.34	1.12	3000.00	10.00	1.00	0.00	0.00	258.00	200.00	8.5
5.09	17.70	3.36	1.40	3000.00	5.00	1.00	0.50	0.00	258.00	200.00	17.0
5.10	1.28	1.37	1.14	3000.00	5.00	3.00	0.50	-1.00	258.00	200.00	8.5
5.11	1.58	1.74	0.73	3000.00	7.00	7.00	0.50	0.00	258.00	200.00	17.0
5.11	1.52	1.38	1.15	3000.00	10.00	7.00	0.00	0.00	258.00	200.00	8.5
5.11	1.36	1.72	0.72	3000.00	7.00	7.00	0.50	0.50	258.00	200.00	17.0
5.11	0.63	1.65	0.69	3000.00	10.00	5.00	1.00	0.00	258.00	200.00	17.0
5.11	1.10	1.69	0.70	3000.00	10.00	3.00	1.00	-0.50	258.00	200.00	17.0
5.12	0.90	1.67	0.69	3000.00	3.00	10.00	-0.50	-1.00	258.00	200.00	17.0
5.12	0.91	1.67	0.70	3000.00	5.00	3.00	0.00	0.50	258.00	200.00	17.0
5.12	0.95	1.33	1.11	3000.00	5.00	3.00	-1.00	1.00	258.00	200.00	8.5
5.13	1.47	1.71	0.71	3000.00	7.00	10.00	0.50	0.00	258.00	200.00	17.0
5.14	0.87	1.65	0.69	3000.00	5.00	3.00	-1.00	0.50	258.00	200.00	17.0
5.14	1.37	1.70	0.71	3000.00	7.00	3.00	-0.50	0.00	258.00	200.00	17.0
5.14	1.44	1.70	0.71	3000.00	10.00	5.00	-0.50	0.00	258.00	200.00	17.0
5.14	1.29	1.68	0.70	3000.00	7.00	5.00	0.50	0.50	258.00	200.00	17.0
5.14	14.45	2.99	1.25	3000.00	3.00	1.00	0.00	0.00	258.00	200.00	17.0
5.15	0.66	1.62	0.67	3000.00	3.00	3.00	-1.00	-1.00	258.00	200.00	17.0
5.15	1.33	1.33	1.11	3000.00	7.00	10.00	0.00	-0.50	258.00	200.00	8.5
5.15	0.84	1.63	0.68	3000.00	10.00	3.00	1.00	0.50	258.00	200.00	17.0
5.16	0.89	1.63	0.68	3000.00	7.00	3.00	-1.00	0.50	258.00	200.00	17.0

Cost function	Sa [μm]	Ablation rate [mm³·min⁻¹]	Ablation eff. [μm³·μJ⁻¹]	Speed [mm/s]	MHz burst	GHz burst	MHz burst Slope	GHz burst Slope	Pulse Width [fs]	Fre-quency [kHz]	Fluence [J·cm⁻²]
5.16	0.61	1.27	1.06	3000.00	7.00	3.00	0.50	1.00	258.00	200.00	8.5
5.16	1.02	1.30	1.09	3000.00	5.00	5.00	-1.00	1.00	258.00	200.00	8.5
5.16	1.95	1.37	1.15	3000.00	10.00	3.00	-0.50	-1.00	258.00	200.00	8.5
5.17	3.25	1.48	1.23	3000.00	7.00	1.00	0.50	0.00	258.00	200.00	8.5
5.17	0.91	1.62	0.68	3000.00	3.00	5.00	-1.00	1.00	258.00	200.00	17.0
5.17	0.78	1.61	0.67	3000.00	3.00	10.00	0.50	-1.00	258.00	200.00	17.0
5.17	1.51	1.68	0.70	3000.00	7.00	10.00	1.00	-1.00	258.00	200.00	17.0
5.17	0.74	1.27	1.06	3000.00	7.00	5.00	0.50	1.00	258.00	200.00	8.5
5.17	0.87	1.62	0.67	3000.00	5.00	5.00	1.00	-1.00	258.00	200.00	17.0
5.17	1.35	1.32	1.10	3000.00	7.00	3.00	0.00	-0.50	258.00	200.00	8.5
5.18	1.36	1.32	1.10	3000.00	7.00	5.00	0.00	-0.50	258.00	200.00	8.5
5.18	0.80	1.61	0.67	3000.00	3.00	10.00	-1.00	-1.00	258.00	200.00	17.0
5.18	1.29	1.65	0.69	3000.00	5.00	3.00	0.00	0.00	258.00	200.00	17.0
5.18	1.74	1.69	0.71	3000.00	7.00	7.00	-0.50	0.00	258.00	200.00	17.0
5.18	1.35	1.31	1.10	3000.00	5.00	10.00	-0.50	1.00	258.00	200.00	8.5
5.19	1.10	1.62	0.68	3000.00	5.00	3.00	-0.50	0.50	258.00	200.00	17.0
5.19	0.96	1.28	1.07	3000.00	7.00	3.00	0.00	0.50	258.00	200.00	8.5
5.19	1.03	1.28	1.07	3000.00	10.00	5.00	-1.00	1.00	258.00	200.00	8.5
5.19	3.70	1.49	1.25	3000.00	3.00	1.00	-0.50	0.00	258.00	200.00	8.5
5.19	0.91	1.60	0.67	3000.00	7.00	3.00	1.00	0.50	258.00	200.00	17.0
5.20	0.99	1.61	0.67	3000.00	10.00	3.00	-1.00	0.50	258.00	200.00	17.0
5.20	1.07	1.61	0.67	3000.00	3.00	5.00	0.00	1.00	258.00	200.00	17.0
5.20	1.82	1.34	1.12	3000.00	7.00	5.00	-0.50	-1.00	258.00	200.00	8.5
5.21	1.08	1.28	1.07	3000.00	7.00	5.00	-1.00	1.00	258.00	200.00	8.5
5.21	0.76	1.58	0.66	3000.00	3.00	7.00	-1.00	-1.00	258.00	200.00	17.0
5.21	3.89	1.49	1.25	3000.00	5.00	1.00	0.50	0.00	258.00	200.00	8.5
5.21	0.95	1.59	0.66	3000.00	5.00	10.00	1.00	-1.00	258.00	200.00	17.0
5.22	1.11	1.27	1.06	3000.00	7.00	5.00	0.00	0.50	258.00	200.00	8.5
5.22	0.74	1.56	0.65	3000.00	3.00	5.00	-1.00	-1.00	258.00	200.00	17.0
5.23	1.10	1.27	1.06	3000.00	5.00	7.00	-1.00	1.00	258.00	200.00	8.5
5.23	1.27	1.28	1.07	3000.00	5.00	5.00	0.50	-1.00	258.00	200.00	8.5
5.23	1.05	1.58	0.66	3000.00	5.00	5.00	-1.00	0.50	258.00	200.00	17.0

Cost function	Sa [µm]	Ablation rate [mm³·min⁻¹]	Ablation eff. [µm³·µJ⁻¹]	Speed [mm/s]	MHz burst	GHz burst	MHz burst Slope	GHz burst Slope	Pulse Width [fs]	Fre-quency [kHz]	Fluence [J·cm⁻²]
5.23	1.07	1.58	0.66	3000.00	7.00	5.00	1.00	0.00	258.00	200.00	17.0
5.23	1.33	1.28	1.07	3000.00	5.00	3.00	-0.50	-1.00	258.00	200.00	8.5
5.24	1.11	1.58	0.66	3000.00	3.00	3.00	0.50	-0.50	258.00	200.00	17.0
5.24	1.07	1.58	0.66	3000.00	10.00	5.00	1.00	-0.50	258.00	200.00	17.0
5.24	0.55	1.52	0.64	3000.00	10.00	7.00	1.00	-0.50	258.00	200.00	17.0
5.25	1.72	1.30	1.09	3000.00	7.00	3.00	-0.50	0.50	258.00	200.00	8.5
5.25	0.87	1.55	0.65	3000.00	3.00	3.00	0.50	1.00	258.00	200.00	17.0
5.25	1.32	1.27	1.06	3000.00	7.00	7.00	0.00	-0.50	258.00	200.00	8.5
5.25	1.78	1.30	1.09	3000.00	7.00	10.00	-0.50	-1.00	258.00	200.00	8.5
5.25	0.79	1.22	1.02	3000.00	3.00	3.00	-1.00	1.00	258.00	200.00	8.5
5.25	0.87	1.55	0.65	3000.00	3.00	7.00	-1.00	1.00	258.00	200.00	17.0
5.25	1.52	1.61	0.67	3000.00	7.00	5.00	-0.50	0.00	258.00	200.00	17.0
5.26	1.38	1.27	1.06	3000.00	5.00	5.00	-0.50	-1.00	258.00	200.00	8.5
5.26	1.26	1.58	0.66	3000.00	3.00	3.00	0.00	-0.50	258.00	200.00	17.0
5.26	1.38	1.59	0.66	3000.00	5.00	5.00	0.00	0.00	258.00	200.00	17.0
5.26	1.13	1.57	0.65	3000.00	7.00	3.00	1.00	-0.50	258.00	200.00	17.0
5.26	1.15	1.57	0.65	3000.00	7.00	5.00	-1.00	0.50	258.00	200.00	17.0
5.26	1.21	1.25	1.04	3000.00	5.00	3.00	0.00	-0.50	258.00	200.00	8.5
5.27	1.84	1.30	1.08	3000.00	10.00	3.00	-0.50	0.50	258.00	200.00	8.5
5.27	0.51	1.50	0.62	3000.00	10.00	10.00	1.00	-0.50	258.00	200.00	17.0
5.27	1.03	1.55	0.64	3000.00	10.00	3.00	1.00	1.00	258.00	200.00	17.0
5.27	0.89	1.22	1.02	3000.00	3.00	3.00	-0.50	-1.00	258.00	200.00	8.5
5.28	1.11	1.55	0.65	3000.00	10.00	5.00	-1.00	0.50	258.00	200.00	17.0
5.28	1.38	1.58	0.66	3000.00	5.00	10.00	-0.50	0.00	258.00	200.00	17.0
5.28	0.73	1.20	1.00	3000.00	3.00	3.00	-0.50	1.00	258.00	200.00	8.5
5.29	1.36	1.57	0.65	3000.00	10.00	3.00	1.00	0.00	258.00	200.00	17.0
5.29	1.29	1.56	0.65	3000.00	5.00	7.00	0.00	0.00	258.00	200.00	17.0
5.29	0.73	1.50	0.63	3000.00	10.00	7.00	1.00	0.00	258.00	200.00	17.0
5.29	1.50	1.58	0.66	3000.00	5.00	10.00	0.00	0.00	258.00	200.00	17.0
5.29	0.86	1.51	0.63	3000.00	10.00	10.00	-1.00	-0.50	258.00	200.00	17.0
5.29	1.35	1.56	0.65	3000.00	5.00	10.00	0.00	0.50	258.00	200.00	17.0
5.29	0.85	1.20	1.00	3000.00	3.00	3.00	0.00	-1.00	258.00	200.00	8.5

Cost function	Sa [μm]	Ablation rate [mm³·min⁻¹]	Ablation eff. [μm³·μJ⁻¹]	Speed [mm/s]	MHz burst	GHz burst	MHz burst Slope	GHz burst Slope	Pulse Width [fs]	Fre-quency [kHz]	Fluence [J·cm⁻²]
5.30	0.48	1.17	0.98	3000.00	5.00	3.00	0.50	1.00	258.00	200.00	8.5
5.30	1.10	1.53	0.64	3000.00	5.00	7.00	-1.00	0.50	258.00	200.00	17.0
5.31	1.27	1.54	0.64	3000.00	7.00	10.00	-1.00	-0.50	258.00	200.00	17.0
5.31	1.27	1.54	0.64	3000.00	3.00	10.00	0.00	-0.50	258.00	200.00	17.0
5.31	1.24	1.53	0.64	3000.00	5.00	10.00	-1.00	-0.50	258.00	200.00	17.0
5.31	1.26	1.22	1.02	3000.00	7.00	10.00	0.50	-1.00	258.00	200.00	8.5
5.32	1.21	1.21	1.01	3000.00	7.00	7.00	-1.00	1.00	258.00	200.00	8.5
5.32	1.30	1.53	0.64	3000.00	3.00	5.00	0.00	-0.50	258.00	200.00	17.0
5.32	0.86	1.18	0.99	3000.00	3.00	10.00	0.00	-1.00	258.00	200.00	8.5
5.32	0.97	1.50	0.62	3000.00	3.00	7.00	0.00	1.00	258.00	200.00	17.0
5.32	0.99	1.50	0.62	3000.00	10.00	7.00	-1.00	0.50	258.00	200.00	17.0
5.33	0.81	1.48	0.62	3000.00	10.00	10.00	1.00	1.00	258.00	200.00	17.0
5.33	1.33	1.21	1.01	3000.00	10.00	7.00	0.50	-0.50	258.00	200.00	8.5
5.33	1.24	1.52	0.63	3000.00	3.00	7.00	0.00	-0.50	258.00	200.00	17.0
5.33	1.33	1.53	0.64	3000.00	7.00	7.00	-1.00	0.50	258.00	200.00	17.0
5.33	1.26	1.21	1.01	3000.00	5.00	10.00	-1.00	1.00	258.00	200.00	8.5
5.33	0.81	1.47	0.61	3000.00	3.00	3.00	0.00	0.50	258.00	200.00	17.0
5.34	1.75	1.24	1.04	3000.00	10.00	5.00	-0.50	0.50	258.00	200.00	8.5
5.34	1.39	1.21	1.01	3000.00	5.00	7.00	-0.50	-1.00	258.00	200.00	8.5
5.35	1.37	1.21	1.01	3000.00	10.00	7.00	-1.00	1.00	258.00	200.00	8.5
5.35	0.77	1.46	0.61	3000.00	10.00	10.00	-1.00	0.50	258.00	200.00	17.0
5.35	1.26	1.50	0.63	3000.00	5.00	10.00	-1.00	0.50	258.00	200.00	17.0
5.35	1.27	1.19	1.00	3000.00	5.00	7.00	0.00	-0.50	258.00	200.00	8.5
5.35	1.29	1.19	1.00	3000.00	10.00	3.00	0.50	-0.50	258.00	200.00	8.5
5.36	1.15	1.49	0.62	3000.00	5.00	7.00	0.00	0.50	258.00	200.00	17.0
5.36	5.02	1.49	1.24	3000.00	3.00	1.00	0.00	0.00	258.00	200.00	8.5
5.36	1.17	1.49	0.62	3000.00	3.00	5.00	0.50	-0.50	258.00	200.00	17.0
5.36	0.57	1.13	0.95	3000.00	5.00	5.00	0.50	1.00	258.00	200.00	8.5
5.36	1.07	1.47	0.61	3000.00	7.00	3.00	1.00	0.00	258.00	200.00	17.0
5.36	1.25	1.49	0.62	3000.00	5.00	5.00	-0.50	0.50	258.00	200.00	17.0
5.37	0.70	1.14	0.95	3000.00	3.00	3.00	0.00	1.00	258.00	200.00	8.5
5.37	1.33	1.50	0.62	3000.00	5.00	5.00	0.00	0.50	258.00	200.00	17.0

Cost function	Sa [μm]	Ablation rate [mm³·min⁻¹]	Ablation eff. [μm³·μJ⁻¹]	Speed [mm/s]	MHz burst	GHz burst	MHz burst Slope	GHz burst Slope	Pulse Width [fs]	Fre-quency [kHz]	Fluence [J·cm⁻²]
5.37	1.00	1.46	0.61	3000.00	3.00	10.00	-1.00	1.00	258.00	200.00	17.0
5.37	0.98	1.46	0.61	3000.00	7.00	3.00	-1.00	-0.50	258.00	200.00	17.0
5.37	1.26	1.49	0.62	3000.00	5.00	7.00	0.50	0.00	258.00	200.00	17.0
5.37	1.20	1.48	0.62	3000.00	5.00	7.00	0.50	0.50	258.00	200.00	17.0
5.38	1.03	1.46	0.61	3000.00	3.00	5.00	-0.50	1.00	258.00	200.00	17.0
5.38	0.87	1.15	0.96	3000.00	3.00	5.00	-1.00	1.00	258.00	200.00	8.5
5.38	1.35	1.49	0.62	3000.00	7.00	10.00	-1.00	0.50	258.00	200.00	17.0
5.38	1.29	1.18	0.98	3000.00	7.00	10.00	-1.00	1.00	258.00	200.00	8.5
5.38	0.97	1.45	0.60	3000.00	3.00	3.00	0.50	0.00	258.00	200.00	17.0
5.38	1.90	1.22	1.02	3000.00	7.00	10.00	-0.50	-0.50	258.00	200.00	8.5
5.38	1.09	1.46	0.61	3000.00	3.00	7.00	0.50	-0.50	258.00	200.00	17.0
5.38	1.10	1.46	0.61	3000.00	5.00	7.00	-1.00	-0.50	258.00	200.00	17.0
5.38	0.89	1.44	0.60	3000.00	5.00	7.00	1.00	-1.00	258.00	200.00	17.0
5.38	1.25	1.17	0.98	3000.00	5.00	7.00	0.50	-1.00	258.00	200.00	8.5
5.39	0.73	1.42	0.59	3000.00	3.00	3.00	0.50	0.50	258.00	200.00	17.0
5.39	0.84	1.43	0.60	3000.00	5.00	5.00	1.00	0.00	258.00	200.00	17.0
5.39	1.27	1.47	0.61	3000.00	5.00	5.00	0.50	0.50	258.00	200.00	17.0
5.39	0.64	1.41	0.59	3000.00	5.00	3.00	1.00	0.50	258.00	200.00	17.0
5.39	0.98	1.44	0.60	3000.00	3.00	3.00	-0.50	-0.50	258.00	200.00	17.0
5.39	2.05	1.23	1.03	3000.00	10.00	5.00	-0.50	-0.50	258.00	200.00	8.5
5.39	0.87	1.13	0.95	3000.00	7.00	10.00	0.50	1.00	258.00	200.00	8.5
5.39	0.77	1.42	0.59	3000.00	5.00	3.00	1.00	0.00	258.00	200.00	17.0
5.40	2.12	1.23	1.03	3000.00	7.00	3.00	-0.50	-0.50	258.00	200.00	8.5
5.40	1.17	1.45	0.61	3000.00	10.00	5.00	1.00	1.00	258.00	200.00	17.0
5.40	0.77	1.12	0.94	3000.00	3.00	5.00	-0.50	1.00	258.00	200.00	8.5
5.41	1.24	1.45	0.61	3000.00	5.00	3.00	-1.00	-0.50	258.00	200.00	17.0
5.41	1.12	1.44	0.60	3000.00	10.00	7.00	-1.00	-0.50	258.00	200.00	17.0
5.41	0.94	1.42	0.59	3000.00	3.00	10.00	0.00	1.00	258.00	200.00	17.0
5.41	1.13	1.44	0.60	3000.00	3.00	3.00	0.00	0.00	258.00	200.00	17.0
5.41	1.09	1.44	0.60	3000.00	7.00	5.00	1.00	-0.50	258.00	200.00	17.0
5.41	1.54	1.18	0.98	3000.00	10.00	3.00	0.00	0.00	258.00	200.00	8.5
5.41	1.19	1.45	0.60	3000.00	5.00	10.00	-0.50	0.50	258.00	200.00	17.0

Cost function	Sa [μm]	Ablation rate [mm³·min⁻¹]	Ablation eff. [μm³·μJ⁻¹]	Speed [mm/s]	MHz burst	GHz burst	MHz burst Slope	GHz burst Slope	Pulse Width [fs]	Fre-quency [kHz]	Fluence [J·cm⁻²]
5.41	1.24	1.15	0.96	3000.00	7.00	10.00	0.00	0.50	258.00	200.00	8.5
5.41	1.27	1.45	0.61	3000.00	7.00	7.00	-1.00	-0.50	258.00	200.00	17.0
5.41	1.54	1.48	0.62	3000.00	7.00	7.00	1.00	1.00	258.00	200.00	17.0
5.41	1.24	1.45	0.60	3000.00	10.00	3.00	-1.00	-0.50	258.00	200.00	17.0
5.41	1.35	1.16	0.97	3000.00	7.00	10.00	0.00	0.00	258.00	200.00	8.5
5.42	1.96	1.21	1.01	3000.00	7.00	7.00	-0.50	-0.50	258.00	200.00	8.5
5.42	0.89	1.41	0.59	3000.00	7.00	3.00	1.00	1.00	258.00	200.00	17.0
5.42	1.20	1.44	0.60	3000.00	5.00	3.00	-0.50	0.00	258.00	200.00	17.0
5.42	2.07	1.21	1.01	3000.00	10.00	7.00	-0.50	-1.00	258.00	200.00	8.5
5.42	2.07	1.21	1.01	3000.00	10.00	10.00	-0.50	-0.50	258.00	200.00	8.5
5.42	1.34	1.45	0.60	3000.00	5.00	7.00	-0.50	0.50	258.00	200.00	17.0
5.42	1.42	1.16	0.97	3000.00	5.00	3.00	-0.50	-0.50	258.00	200.00	8.5
5.43	1.37	1.15	0.96	3000.00	7.00	5.00	0.50	-0.50	258.00	200.00	8.5
5.43	1.01	1.41	0.59	3000.00	3.00	7.00	-0.50	1.00	258.00	200.00	17.0
5.43	1.35	1.15	0.96	3000.00	10.00	5.00	0.50	-0.50	258.00	200.00	8.5
5.43	1.35	1.15	0.96	3000.00	7.00	3.00	0.50	-0.50	258.00	200.00	8.5
5.43	1.25	1.43	0.60	3000.00	5.00	7.00	-0.50	0.00	258.00	200.00	17.0
5.44	1.13	1.42	0.59	3000.00	5.00	5.00	-1.00	-0.50	258.00	200.00	17.0
5.44	1.27	1.43	0.59	3000.00	7.00	5.00	1.00	1.00	258.00	200.00	17.0
5.44	1.36	1.44	0.60	3000.00	5.00	10.00	0.50	0.00	258.00	200.00	17.0
5.45	1.29	1.43	0.59	3000.00	3.00	7.00	0.50	1.00	258.00	200.00	17.0
5.45	1.13	1.41	0.59	3000.00	3.00	10.00	0.50	-0.50	258.00	200.00	17.0
5.45	0.85	1.09	0.91	3000.00	5.00	3.00	0.00	0.50	258.00	200.00	8.5
5.46	1.21	1.41	0.59	3000.00	10.00	5.00	-1.00	-0.50	258.00	200.00	17.0
5.46	1.24	1.41	0.59	3000.00	7.00	5.00	-1.00	-0.50	258.00	200.00	17.0
5.46	1.66	1.15	0.96	3000.00	7.00	7.00	-0.50	0.50	258.00	200.00	8.5
5.46	1.29	1.12	0.94	3000.00	5.00	10.00	0.00	-0.50	258.00	200.00	8.5
5.46	1.14	1.40	0.58	3000.00	3.00	10.00	-0.50	-0.50	258.00	200.00	17.0
5.46	1.08	1.10	0.92	3000.00	7.00	7.00	0.00	0.50	258.00	200.00	8.5
5.46	2.60	0.46	1.93	3000.00	1.00	1.00	0.00	0.00	258.00	200.00	1.7
5.47	1.26	1.41	0.59	3000.00	5.00	5.00	-0.50	0.00	258.00	200.00	17.0
5.47	0.93	1.37	0.57	3000.00	3.00	10.00	-0.50	1.00	258.00	200.00	17.0

Cost function	Sa [μm]	Ablation rate [mm³·min⁻¹]	Ablation eff. [μm³·μJ⁻¹]	Speed [mm/s]	MHz burst	GHz burst	MHz burst Slope	GHz burst Slope	Pulse Width [fs]	Fre-quency [kHz]	Fluence [J·cm⁻²]
5.47	0.91	1.09	0.91	3000.00	3.00	5.00	0.00	-1.00	258.00	200.00	8.5
5.47	0.94	1.37	0.57	3000.00	3.00	10.00	0.50	1.00	258.00	200.00	17.0
5.48	0.81	1.07	0.90	3000.00	7.00	7.00	0.50	1.00	258.00	200.00	8.5
5.48	0.95	1.37	0.57	3000.00	5.00	3.00	1.00	-0.50	258.00	200.00	17.0
5.48	1.33	1.40	0.58	3000.00	5.00	10.00	0.50	0.50	258.00	200.00	17.0
5.48	1.09	1.38	0.57	3000.00	3.00	5.00	-0.50	-0.50	258.00	200.00	17.0
5.49	1.96	1.16	0.97	3000.00	7.00	5.00	-0.50	-0.50	258.00	200.00	8.5
5.49	1.09	1.37	0.57	3000.00	3.00	7.00	-0.50	-0.50	258.00	200.00	17.0
5.49	1.32	1.39	0.58	3000.00	7.00	7.00	1.00	0.00	258.00	200.00	17.0
5.49	0.72	1.06	0.88	3000.00	3.00	5.00	0.00	1.00	258.00	200.00	8.5
5.49	1.20	1.38	0.57	3000.00	7.00	7.00	1.00	-0.50	258.00	200.00	17.0
5.49	0.73	1.33	0.55	3000.00	10.00	10.00	1.00	0.00	258.00	200.00	17.0
5.50	1.33	1.39	0.58	3000.00	3.00	5.00	0.00	0.00	258.00	200.00	17.0
5.50	1.82	1.43	0.60	3000.00	7.00	10.00	1.00	1.00	258.00	200.00	17.0
5.50	0.79	1.33	0.55	3000.00	5.00	3.00	1.00	1.00	258.00	200.00	17.0
5.50	1.26	1.09	0.91	3000.00	5.00	5.00	0.00	-0.50	258.00	200.00	8.5
5.50	0.75	1.32	0.55	3000.00	10.00	5.00	1.00	0.50	258.00	200.00	17.0
5.51	0.92	1.06	0.89	3000.00	3.00	7.00	-0.50	-1.00	258.00	200.00	8.5
5.51	1.23	1.37	0.57	3000.00	5.00	7.00	1.00	1.00	258.00	200.00	17.0
5.51	2.21	1.46	0.61	3000.00	10.00	7.00	1.00	1.00	258.00	200.00	17.0
5.52	1.27	1.08	0.90	3000.00	10.00	10.00	-1.00	1.00	258.00	200.00	8.5
5.52	15.21	2.75	1.14	3000.00	3.00	1.00	0.50	0.00	258.00	200.00	17.0
5.52	0.60	1.03	0.86	3000.00	3.00	7.00	0.00	1.00	258.00	200.00	8.5
5.52	2.04	1.14	0.95	3000.00	10.00	3.00	-0.50	-0.50	258.00	200.00	8.5
5.52	1.67	1.11	0.93	3000.00	7.00	5.00	-0.50	0.50	258.00	200.00	8.5
5.52	0.89	1.32	0.55	3000.00	5.00	10.00	1.00	1.00	258.00	200.00	17.0
5.53	1.39	1.09	0.91	3000.00	7.00	5.00	0.00	0.00	258.00	200.00	8.5
5.53	0.71	1.03	0.86	3000.00	3.00	7.00	-0.50	1.00	258.00	200.00	8.5
5.53	1.09	1.34	0.56	3000.00	7.00	10.00	1.00	-0.50	258.00	200.00	17.0
5.53	0.82	1.04	0.87	3000.00	3.00	7.00	0.00	-1.00	258.00	200.00	8.5
5.54	1.48	1.09	0.91	3000.00	5.00	10.00	-0.50	-0.50	258.00	200.00	8.5
5.54	1.09	1.33	0.55	3000.00	3.00	5.00	0.50	0.00	258.00	200.00	17.0

Cost function	Sa [μm]	Ablation rate [mm³·min⁻¹]	Ablation eff. [μm³·μJ⁻¹]	Speed [mm/s]	MHz burst	GHz burst	MHz burst Slope	GHz burst Slope	Pulse Width [fs]	Fre-quency [kHz]	Fluence [J·cm⁻²]
5.54	7.24	1.54	1.28	3000.00	1.00	1.00	0.00	0.00	258.00	200.00	8.5
5.55	1.26	1.06	0.89	3000.00	5.00	10.00	0.50	-1.00	258.00	200.00	8.5
5.55	0.74	1.28	0.54	3000.00	3.00	3.00	-1.00	0.50	258.00	200.00	17.0
5.55	1.15	1.05	0.88	3000.00	10.00	3.00	-1.00	0.50	258.00	200.00	8.5
5.55	0.87	1.03	0.86	3000.00	3.00	7.00	-1.00	1.00	258.00	200.00	8.5
5.55	1.10	1.04	0.87	3000.00	5.00	3.00	-1.00	-1.00	258.00	200.00	8.5
5.55	1.33	1.06	0.89	3000.00	5.00	10.00	-1.00	-1.00	258.00	200.00	8.5
5.55	1.33	1.06	0.89	3000.00	10.00	10.00	0.50	-0.50	258.00	200.00	8.5
5.56	1.75	1.09	0.91	3000.00	10.00	7.00	-0.50	0.50	258.00	200.00	8.5
5.56	0.60	0.38	1.58	3000.00	3.00	5.00	-1.00	1.00	258.00	200.00	1.7
5.56	1.61	1.08	0.90	3000.00	5.00	10.00	-0.50	0.00	258.00	200.00	8.5
5.56	0.72	1.01	0.84	3000.00	3.00	10.00	-0.50	1.00	258.00	200.00	8.5
5.57	2.04	1.11	0.93	3000.00	10.00	7.00	-0.50	-0.50	258.00	200.00	8.5
5.57	1.15	1.04	0.87	3000.00	10.00	7.00	0.50	0.50	258.00	200.00	8.5
5.57	1.01	1.03	0.86	3000.00	10.00	3.00	0.50	0.50	258.00	200.00	8.5
5.57	1.26	1.04	0.87	3000.00	5.00	3.00	0.50	-0.50	258.00	200.00	8.5
5.57	0.84	1.27	0.53	3000.00	3.00	3.00	1.00	0.50	258.00	200.00	17.0
5.57	1.35	1.05	0.88	3000.00	5.00	3.00	-0.50	0.50	258.00	200.00	8.5
5.58	0.91	1.28	0.53	3000.00	7.00	5.00	1.00	0.50	258.00	200.00	17.0
5.58	1.19	1.30	0.54	3000.00	5.00	3.00	-1.00	0.00	258.00	200.00	17.0
5.58	0.96	1.01	0.85	3000.00	3.00	5.00	-0.50	-1.00	258.00	200.00	8.5
5.59	0.81	1.26	0.52	3000.00	3.00	3.00	-1.00	-0.50	258.00	200.00	17.0
5.59	1.13	1.29	0.54	3000.00	7.00	3.00	-1.00	0.00	258.00	200.00	17.0
5.59	0.65	0.98	0.82	3000.00	5.00	7.00	0.50	1.00	258.00	200.00	8.5
5.59	0.48	0.37	1.53	3000.00	3.00	3.00	-0.50	1.00	258.00	200.00	1.7
5.59	1.82	1.07	0.90	3000.00	7.00	10.00	-0.50	0.00	258.00	200.00	8.5
5.60	0.93	1.00	0.84	3000.00	3.00	10.00	-0.50	-1.00	258.00	200.00	8.5
5.60	0.52	0.37	1.53	3000.00	3.00	3.00	0.00	1.00	258.00	200.00	1.7
5.60	0.98	1.26	0.53	3000.00	3.00	10.00	-1.00	-0.50	258.00	200.00	17.0
5.60	1.15	1.28	0.53	3000.00	10.00	3.00	-1.00	0.00	258.00	200.00	17.0
5.60	1.06	1.01	0.84	3000.00	10.00	3.00	-1.00	-1.00	258.00	200.00	8.5
5.61	0.89	1.25	0.52	3000.00	5.00	5.00	1.00	-0.50	258.00	200.00	17.0

Cost function	Sa [μm]	Ablation rate [mm³·min⁻¹]	Ablation eff. [μm³·μJ⁻¹]	Speed [mm/s]	MHz burst	GHz burst	MHz burst Slope	GHz burst Slope	Pulse Width [fs]	Fre-quency [kHz]	Fluence [J·cm⁻²]
5.61	1.47	1.04	0.87	3000.00	5.00	5.00	-0.50	-0.50	258.00	200.00	8.5
5.61	0.42	0.36	1.50	3000.00	10.00	10.00	1.00	0.00	258.00	200.00	1.7
5.61	17.66	2.91	1.21	3000.00	1.00	1.00	0.00	0.00	258.00	200.00	17.0
5.62	1.33	1.02	0.85	3000.00	7.00	7.00	0.50	-0.50	258.00	200.00	8.5
5.62	1.30	1.01	0.85	3000.00	7.00	10.00	-1.00	-1.00	258.00	200.00	8.5
5.63	0.57	0.95	0.80	3000.00	5.00	10.00	0.50	1.00	258.00	200.00	8.5
5.63	1.07	0.99	0.83	3000.00	10.00	5.00	0.50	0.50	258.00	200.00	8.5
5.63	1.75	1.04	0.87	3000.00	7.00	10.00	-0.50	0.50	258.00	200.00	8.5
5.63	0.41	0.35	1.48	3000.00	3.00	3.00	-0.50	0.50	258.00	200.00	1.7
5.63	0.98	1.24	0.52	3000.00	3.00	3.00	-0.50	0.00	258.00	200.00	17.0
5.63	1.25	1.26	0.53	3000.00	5.00	10.00	-1.00	0.00	258.00	200.00	17.0
5.63	0.60	1.20	0.50	3000.00	3.00	3.00	1.00	-1.00	258.00	200.00	17.0
5.63	0.65	0.95	0.80	3000.00	3.00	10.00	0.00	1.00	258.00	200.00	8.5
5.64	1.42	1.01	0.85	3000.00	7.00	3.00	0.00	0.00	258.00	200.00	8.5
5.64	0.76	1.21	0.50	3000.00	3.00	3.00	-0.50	0.50	258.00	200.00	17.0
5.64	0.76	0.96	0.80	3000.00	7.00	3.00	0.50	0.50	258.00	200.00	8.5
5.64	1.03	0.98	0.82	3000.00	7.00	3.00	-1.00	-1.00	258.00	200.00	8.5
5.64	0.93	1.22	0.51	3000.00	5.00	7.00	1.00	0.00	258.00	200.00	17.0
5.65	1.20	0.99	0.83	3000.00	10.00	7.00	-1.00	-1.00	258.00	200.00	8.5
5.65	1.28	1.00	0.83	3000.00	10.00	10.00	-1.00	-1.00	258.00	200.00	8.5
5.65	1.82	1.04	0.87	3000.00	10.00	10.00	-0.50	0.50	258.00	200.00	8.5
5.65	1.37	1.00	0.84	3000.00	7.00	10.00	0.50	-0.50	258.00	200.00	8.5
5.65	1.19	0.98	0.82	3000.00	10.00	5.00	-1.00	-1.00	258.00	200.00	8.5
5.65	0.59	0.94	0.78	3000.00	5.00	3.00	0.50	0.50	258.00	200.00	8.5
5.66	1.51	1.01	0.84	3000.00	5.00	7.00	-0.50	-0.50	258.00	200.00	8.5
5.66	0.86	1.20	0.50	3000.00	3.00	5.00	-1.00	-0.50	258.00	200.00	17.0
5.66	1.00	0.97	0.81	3000.00	5.00	3.00	0.50	0.00	258.00	200.00	8.5
5.66	1.24	0.98	0.82	3000.00	5.00	7.00	-1.00	-1.00	258.00	200.00	8.5
5.66	1.79	1.03	0.86	3000.00	7.00	3.00	-0.50	0.00	258.00	200.00	8.5
5.66	0.78	1.19	0.50	3000.00	5.00	5.00	1.00	0.50	258.00	200.00	17.0
5.66	1.01	1.21	0.51	3000.00	10.00	10.00	-1.00	0.00	258.00	200.00	17.0
5.66	0.90	1.20	0.50	3000.00	3.00	7.00	-1.00	-0.50	258.00	200.00	17.0

Cost function	Sa [μm]	Ablation rate [$mm^3 \cdot min^{-1}$]	Ablation eff. [$\mu m^3 \cdot \mu J^{-1}$]	Speed [mm/s]	MHz burst	GHz burst	MHz burst Slope	GHz burst Slope	Pulse Width [fs]	Frequency [kHz]	Fluence [$J \cdot cm^{-2}$]
5.67	1.37	0.99	0.83	3000.00	7.00	7.00	0.00	0.00	258.00	200.00	8.5
5.67	0.88	1.20	0.50	3000.00	5.00	7.00	1.00	-0.50	258.00	200.00	17.0
5.67	1.09	0.97	0.81	3000.00	5.00	3.00	-1.00	0.50	258.00	200.00	8.5
5.67	0.89	1.19	0.50	3000.00	3.00	7.00	0.50	0.50	258.00	200.00	17.0
5.67	0.90	1.19	0.50	3000.00	3.00	5.00	0.50	0.50	258.00	200.00	17.0
5.68	0.95	1.19	0.50	3000.00	7.00	10.00	1.00	0.00	258.00	200.00	17.0
5.68	0.80	0.94	0.78	3000.00	3.00	3.00	0.50	-1.00	258.00	200.00	8.5
5.68	1.23	1.22	0.51	3000.00	3.00	7.00	0.00	0.50	258.00	200.00	17.0
5.68	12.70	2.36	0.98	3000.00	7.00	1.00	1.00	0.00	258.00	200.00	17.0
5.68	1.19	0.97	0.81	3000.00	10.00	3.00	0.50	0.00	258.00	200.00	8.5
5.69	1.08	0.95	0.80	3000.00	7.00	5.00	-1.00	0.50	258.00	200.00	8.5
5.69	1.08	0.95	0.80	3000.00	7.00	3.00	0.50	0.00	258.00	200.00	8.5
5.69	1.25	0.97	0.81	3000.00	10.00	5.00	0.50	0.00	258.00	200.00	8.5
5.69	1.34	1.22	0.51	3000.00	7.00	10.00	-1.00	0.00	258.00	200.00	17.0
5.69	1.30	0.37	1.53	3000.00	3.00	1.00	1.00	0.00	258.00	200.00	1.7
5.69	1.03	1.19	0.49	3000.00	3.00	5.00	-1.00	0.50	258.00	200.00	17.0
5.70	0.94	1.18	0.49	3000.00	5.00	10.00	1.00	-0.50	258.00	200.00	17.0
5.70	1.32	1.22	0.51	3000.00	3.00	10.00	0.00	0.50	258.00	200.00	17.0
5.70	1.03	0.94	0.79	3000.00	7.00	5.00	0.50	0.50	258.00	200.00	8.5
5.70	1.10	1.19	0.50	3000.00	3.00	10.00	0.50	0.50	258.00	200.00	17.0
5.70	1.13	1.20	0.50	3000.00	3.00	7.00	-0.50	0.50	258.00	200.00	17.0
5.70	0.82	0.92	0.77	3000.00	5.00	5.00	0.00	0.50	258.00	200.00	8.5
5.70	1.00	0.35	1.48	3000.00	5.00	3.00	-1.00	1.00	258.00	200.00	1.7
5.70	1.22	0.95	0.80	3000.00	5.00	5.00	-1.00	-1.00	258.00	200.00	8.5
5.70	1.10	1.19	0.50	3000.00	3.00	5.00	0.00	0.50	258.00	200.00	17.0
5.70	0.85	0.92	0.77	3000.00	3.00	5.00	0.50	-1.00	258.00	200.00	8.5
5.70	1.16	0.95	0.79	3000.00	7.00	5.00	0.50	0.00	258.00	200.00	8.5
5.70	1.17	0.95	0.79	3000.00	7.00	5.00	-1.00	-1.00	258.00	200.00	8.5
5.70	1.04	0.35	1.48	3000.00	7.00	1.00	1.00	0.00	258.00	200.00	1.7
5.71	0.95	0.93	0.78	3000.00	3.00	10.00	-1.00	1.00	258.00	200.00	8.5
5.71	1.04	0.94	0.78	3000.00	3.00	3.00	0.00	-0.50	258.00	200.00	8.5
5.71	1.13	0.94	0.79	3000.00	5.00	7.00	-1.00	0.50	258.00	200.00	8.5

Cost function	Sa [μm]	Ablation rate [mm³·min⁻¹]	Ablation eff. [μm³·μJ⁻¹]	Speed [mm/s]	MHz burst	GHz burst	MHz burst Slope	GHz burst Slope	Pulse Width [fs]	Fre-quency [kHz]	Fluence [J·cm⁻²]
5.71	1.30	0.95	0.80	3000.00	5.00	5.00	0.50	-0.50	258.00	200.00	8.5
5.71	0.89	0.35	1.45	3000.00	3.00	3.00	0.50	1.00	258.00	200.00	1.7
5.71	1.25	0.95	0.79	3000.00	5.00	7.00	0.00	0.00	258.00	200.00	8.5
5.72	1.27	0.95	0.79	3000.00	10.00	7.00	0.50	0.00	258.00	200.00	8.5
5.72	0.94	0.35	1.45	3000.00	7.00	3.00	-1.00	1.00	258.00	200.00	1.7
5.72	0.54	0.34	1.40	3000.00	3.00	5.00	-0.50	1.00	258.00	200.00	1.7
5.72	0.57	0.34	1.40	3000.00	3.00	7.00	-0.50	1.00	258.00	200.00	1.7
5.72	1.18	0.94	0.78	3000.00	10.00	10.00	0.50	0.50	258.00	200.00	8.5
5.72	1.87	0.99	0.83	3000.00	10.00	5.00	-0.50	0.00	258.00	200.00	8.5
5.72	1.06	1.17	0.49	3000.00	3.00	5.00	-0.50	0.50	258.00	200.00	17.0
5.73	1.18	1.18	0.49	3000.00	5.00	5.00	-1.00	0.00	258.00	200.00	17.0
5.73	0.67	1.12	0.47	3000.00	3.00	5.00	1.00	-1.00	258.00	200.00	17.0
5.73	0.45	0.33	1.38	3000.00	10.00	5.00	-1.00	-1.00	258.00	200.00	1.7
5.73	1.39	0.95	0.79	3000.00	5.00	5.00	-0.50	0.50	258.00	200.00	8.5
5.73	0.86	0.91	0.76	3000.00	3.00	7.00	-1.00	-1.00	258.00	200.00	8.5
5.73	1.24	0.94	0.78	3000.00	7.00	7.00	-1.00	-1.00	258.00	200.00	8.5
5.74	1.07	0.92	0.77	3000.00	7.00	3.00	-1.00	0.50	258.00	200.00	8.5
5.74	0.67	1.12	0.47	3000.00	3.00	7.00	1.00	-1.00	258.00	200.00	17.0
5.74	0.63	1.11	0.46	3000.00	3.00	10.00	1.00	-1.00	258.00	200.00	17.0
5.74	1.03	0.91	0.76	3000.00	10.00	5.00	-1.00	0.50	258.00	200.00	8.5
5.74	1.50	0.95	0.79	3000.00	5.00	10.00	-0.50	0.50	258.00	200.00	8.5
5.74	1.27	0.93	0.78	3000.00	5.00	10.00	-1.00	0.50	258.00	200.00	8.5
5.74	0.59	1.10	0.46	3000.00	3.00	3.00	1.00	0.00	258.00	200.00	17.0
5.74	1.21	1.16	0.49	3000.00	5.00	7.00	-1.00	0.00	258.00	200.00	17.0
5.74	1.16	1.16	0.48	3000.00	7.00	5.00	-1.00	0.00	258.00	200.00	17.0
5.75	1.31	1.17	0.49	3000.00	3.00	5.00	-0.50	0.00	258.00	200.00	17.0
5.75	1.43	1.18	0.49	3000.00	3.00	10.00	-1.00	0.50	258.00	200.00	17.0
5.75	2.67	0.39	1.63	3000.00	3.00	1.00	-0.50	0.00	258.00	200.00	1.7
5.76	1.18	1.15	0.48	3000.00	3.00	7.00	-1.00	0.50	258.00	200.00	17.0
5.76	1.08	1.13	0.47	3000.00	10.00	10.00	1.00	0.50	258.00	200.00	17.0
5.77	1.84	0.96	0.80	3000.00	10.00	3.00	-0.50	0.00	258.00	200.00	8.5
5.77	0.60	0.32	1.35	3000.00	3.00	3.00	1.00	1.00	258.00	200.00	1.7

Cost function	Sa [μm]	Ablation rate [mm³·min⁻¹]	Ablation eff. [μm³·μJ⁻¹]	Speed [mm/s]	MHz burst	GHz burst	MHz burst Slope	GHz burst Slope	Pulse Width [fs]	Fre-quency [kHz]	Fluence [J·cm⁻²]
5.77	0.99	1.12	0.47	3000.00	7.00	7.00	1.00	0.50	258.00	200.00	17.0
5.77	4.78	1.49	0.62	3000.00	3.00	5.00	0.50	1.00	258.00	200.00	17.0
5.78	0.84	1.10	0.46	3000.00	3.00	3.00	-1.00	0.00	258.00	200.00	17.0
5.78	1.36	0.91	0.76	3000.00	10.00	10.00	0.50	0.00	258.00	200.00	8.5
5.78	1.38	1.15	0.48	3000.00	10.00	5.00	-1.00	0.00	258.00	200.00	17.0
5.78	1.09	1.12	0.47	3000.00	10.00	7.00	-1.00	0.00	258.00	200.00	17.0
5.78	0.51	0.32	1.33	3000.00	3.00	7.00	-0.50	0.50	258.00	200.00	1.7
5.79	0.87	0.87	0.73	3000.00	3.00	3.00	-1.00	-1.00	258.00	200.00	8.5
5.79	1.03	0.88	0.74	3000.00	5.00	5.00	-1.00	0.50	258.00	200.00	8.5
5.79	1.03	0.88	0.74	3000.00	7.00	7.00	0.50	0.50	258.00	200.00	8.5
5.79	1.36	1.14	0.48	3000.00	7.00	7.00	-1.00	0.00	258.00	200.00	17.0
5.79	0.53	0.32	1.33	3000.00	3.00	10.00	0.00	1.00	258.00	200.00	1.7
5.79	1.89	0.95	0.79	3000.00	10.00	10.00	-0.50	0.00	258.00	200.00	8.5
5.79	0.36	0.31	1.30	3000.00	3.00	3.00	0.00	0.50	258.00	200.00	1.7
5.79	1.84	0.94	0.79	3000.00	7.00	7.00	-0.50	0.00	258.00	200.00	8.5
5.80	1.03	0.88	0.73	3000.00	5.00	10.00	0.00	0.50	258.00	200.00	8.5
5.80	1.20	0.89	0.74	3000.00	5.00	3.00	0.00	0.00	258.00	200.00	8.5
5.80	1.27	0.89	0.75	3000.00	5.00	10.00	0.00	0.00	258.00	200.00	8.5
5.80	1.02	1.10	0.46	3000.00	7.00	10.00	1.00	0.50	258.00	200.00	17.0
5.80	1.41	0.90	0.75	3000.00	5.00	7.00	-0.50	0.50	258.00	200.00	8.5
5.80	1.34	0.89	0.75	3000.00	5.00	10.00	0.50	-0.50	258.00	200.00	8.5
5.81	0.91	1.08	0.45	3000.00	5.00	7.00	1.00	0.50	258.00	200.00	17.0
5.81	1.12	0.87	0.73	3000.00	7.00	7.00	-1.00	0.50	258.00	200.00	8.5
5.82	0.91	0.85	0.71	3000.00	3.00	7.00	0.50	-1.00	258.00	200.00	8.5
5.82	1.08	0.86	0.72	3000.00	10.00	7.00	-1.00	0.50	258.00	200.00	8.5
5.82	1.71	0.91	0.76	3000.00	7.00	10.00	-1.00	-0.50	258.00	200.00	8.5
5.82	1.50	1.13	0.47	3000.00	3.00	10.00	-0.50	0.50	258.00	200.00	17.0
5.82	1.35	1.11	0.46	3000.00	3.00	10.00	-0.50	0.00	258.00	200.00	17.0
5.82	0.89	0.85	0.71	3000.00	5.00	7.00	0.00	0.50	258.00	200.00	8.5
5.82	1.73	0.91	0.76	3000.00	5.00	10.00	-1.00	-0.50	258.00	200.00	8.5
5.83	0.87	0.84	0.70	3000.00	3.00	10.00	-1.00	-1.00	258.00	200.00	8.5
5.83	0.88	1.06	0.44	3000.00	5.00	10.00	1.00	0.50	258.00	200.00	17.0

Cost function	Sa [μm]	Ablation rate [mm³·min⁻¹]	Ablation eff. [μm³·μJ⁻¹]	Speed [mm/s]	MHz burst	GHz burst	MHz burst Slope	GHz burst Slope	Pulse Width [fs]	Fre-quency [kHz]	Fluence [J·cm⁻²]
5.83	1.28	0.87	0.73	3000.00	7.00	10.00	-1.00	0.50	258.00	200.00	8.5
5.83	0.73	0.31	1.30	3000.00	3.00	10.00	-0.50	1.00	258.00	200.00	1.7
5.83	1.07	1.07	0.45	3000.00	3.00	7.00	0.50	0.00	258.00	200.00	17.0
5.84	0.77	0.83	0.69	3000.00	5.00	5.00	0.50	0.50	258.00	200.00	8.5
5.84	1.09	0.85	0.71	3000.00	3.00	5.00	0.00	-0.50	258.00	200.00	8.5
5.84	0.77	0.31	1.30	3000.00	3.00	7.00	1.00	1.00	258.00	200.00	1.7
5.84	2.80	0.37	1.55	3000.00	3.00	1.00	-1.00	0.00	258.00	200.00	1.7
5.84	1.28	0.86	0.72	3000.00	10.00	10.00	-1.00	0.50	258.00	200.00	8.5
5.85	0.64	0.81	0.68	3000.00	3.00	3.00	0.00	0.50	258.00	200.00	8.5
5.85	0.46	0.30	1.25	3000.00	5.00	3.00	-1.00	0.50	258.00	200.00	1.7
5.85	1.32	1.08	0.45	3000.00	3.00	7.00	0.00	0.00	258.00	200.00	17.0
5.85	1.06	0.84	0.70	3000.00	3.00	3.00	-0.50	-0.50	258.00	200.00	8.5
5.86	1.02	0.83	0.70	3000.00	3.00	3.00	-1.00	0.50	258.00	200.00	8.5
5.86	9.31	1.49	1.24	3000.00	3.00	1.00	0.50	0.00	258.00	200.00	8.5
5.86	0.73	1.02	0.42	3000.00	3.00	5.00	1.00	0.00	258.00	200.00	17.0
5.86	0.97	0.83	0.69	3000.00	3.00	5.00	-1.00	-1.00	258.00	200.00	8.5
5.86	1.27	0.85	0.71	3000.00	5.00	7.00	0.50	-0.50	258.00	200.00	8.5
5.86	1.30	0.85	0.71	3000.00	10.00	3.00	-1.00	-0.50	258.00	200.00	8.5
5.86	0.58	1.00	0.42	3000.00	3.00	3.00	1.00	-0.50	258.00	200.00	17.0
5.86	2.80	0.37	1.53	3000.00	3.00	1.00	0.00	0.00	258.00	200.00	1.7
5.86	0.80	0.31	1.28	3000.00	7.00	5.00	-1.00	1.00	258.00	200.00	1.7
5.87	0.80	0.81	0.68	3000.00	3.00	3.00	-0.50	0.50	258.00	200.00	8.5
5.87	1.80	0.89	0.74	3000.00	7.00	5.00	-0.50	0.00	258.00	200.00	8.5
5.87	1.32	1.07	0.44	3000.00	3.00	10.00	0.00	0.00	258.00	200.00	17.0
5.87	1.17	0.83	0.70	3000.00	3.00	10.00	-0.50	-0.50	258.00	200.00	8.5
5.87	1.10	0.83	0.69	3000.00	3.00	7.00	0.00	-0.50	258.00	200.00	8.5
5.88	1.13	0.83	0.69	3000.00	5.00	5.00	0.50	0.00	258.00	200.00	8.5
5.88	0.51	0.29	1.23	3000.00	3.00	5.00	0.50	1.00	258.00	200.00	1.7
5.88	1.15	0.83	0.69	3000.00	3.00	10.00	0.00	-0.50	258.00	200.00	8.5
5.88	9.20	1.46	1.22	3000.00	3.00	1.00	1.00	0.00	258.00	200.00	8.5
5.88	1.11	0.82	0.69	3000.00	3.00	5.00	-0.50	-0.50	258.00	200.00	8.5
5.89	0.99	0.81	0.68	3000.00	3.00	3.00	0.50	-0.50	258.00	200.00	8.5

Cost function	Sa [μm]	Ablation rate [mm³·min⁻¹]	Ablation eff. [μm³·μJ⁻¹]	Speed [mm/s]	MHz burst	GHz burst	MHz burst Slope	GHz burst Slope	Pulse Width [fs]	Fre-quency [kHz]	Fluence [J·cm⁻²]
5.89	1.83	0.88	0.73	3000.00	10.00	7.00	-0.50	0.00	258.00	200.00	8.5
5.89	1.25	1.04	0.44	3000.00	3.00	7.00	-0.50	0.00	258.00	200.00	17.0
5.89	0.99	1.02	0.42	3000.00	3.00	7.00	1.00	0.50	258.00	200.00	17.0
5.89	0.45	0.29	1.20	3000.00	3.00	7.00	0.00	1.00	258.00	200.00	1.7
5.90	1.06	1.02	0.43	3000.00	5.00	10.00	1.00	0.00	258.00	200.00	17.0
5.90	3.08	0.37	1.53	3000.00	3.00	1.00	0.50	0.00	258.00	200.00	1.7
5.90	1.25	0.82	0.69	3000.00	5.00	5.00	0.00	0.00	258.00	200.00	8.5
5.90	9.99	1.90	0.79	3000.00	7.00	5.00	1.00	-1.00	258.00	200.00	17.0
5.90	1.04	1.01	0.42	3000.00	3.00	5.00	-1.00	0.00	258.00	200.00	17.0
5.91	0.34	0.28	1.18	3000.00	5.00	3.00	0.00	0.00	258.00	200.00	1.7
5.91	1.52	0.84	0.70	3000.00	5.00	7.00	-0.50	0.00	258.00	200.00	8.5
5.91	1.19	1.02	0.43	3000.00	3.00	10.00	-1.00	0.00	258.00	200.00	17.0
5.91	0.57	0.29	1.20	3000.00	5.00	5.00	0.50	1.00	258.00	200.00	1.7
5.91	0.58	0.76	0.64	3000.00	3.00	10.00	0.50	1.00	258.00	200.00	8.5
5.91	0.61	0.76	0.64	3000.00	3.00	3.00	0.50	0.50	258.00	200.00	8.5
5.92	1.32	1.03	0.43	3000.00	3.00	5.00	1.00	0.50	258.00	200.00	17.0
5.92	1.03	0.79	0.66	3000.00	5.00	10.00	0.50	0.50	258.00	200.00	8.5
5.92	1.20	1.01	0.42	3000.00	3.00	10.00	0.50	0.00	258.00	200.00	17.0
5.92	0.48	0.28	1.18	3000.00	10.00	5.00	-1.00	0.50	258.00	200.00	1.7
5.92	1.13	0.80	0.67	3000.00	7.00	10.00	0.50	0.50	258.00	200.00	8.5
5.92	0.32	0.28	1.15	3000.00	10.00	3.00	-1.00	-1.00	258.00	200.00	1.7
5.92	0.80	0.97	0.40	3000.00	3.00	10.00	1.00	0.50	258.00	200.00	17.0
5.93	0.72	0.29	1.20	3000.00	3.00	7.00	-1.00	1.00	258.00	200.00	1.7
5.93	0.73	0.29	1.20	3000.00	7.00	10.00	1.00	-1.00	258.00	200.00	1.7
5.94	0.94	0.77	0.65	3000.00	3.00	7.00	-1.00	0.50	258.00	200.00	8.5
5.94	1.20	1.00	0.42	3000.00	3.00	7.00	-1.00	0.00	258.00	200.00	17.0
5.94	0.80	0.29	1.20	3000.00	10.00	3.00	-1.00	0.00	258.00	200.00	1.7
5.94	0.88	0.77	0.64	3000.00	10.00	3.00	1.00	-1.00	258.00	200.00	8.5
5.94	0.58	0.74	0.62	3000.00	3.00	3.00	0.50	0.00	258.00	200.00	8.5
5.94	1.13	0.79	0.66	3000.00	3.00	7.00	-0.50	-0.50	258.00	200.00	8.5
5.94	2.86	0.35	1.45	3000.00	7.00	1.00	-1.00	0.00	258.00	200.00	1.7
5.94	1.05	0.29	1.23	3000.00	10.00	3.00	-1.00	1.00	258.00	200.00	1.7

Cost function	Sa [μm]	Ablation rate [$mm^3 \cdot min^{-1}$]	Ablation eff. [$\mu m^3 \cdot \mu J^{-1}$]	Speed [mm/s]	MHz burst	GHz burst	MHz burst Slope	GHz burst Slope	Pulse Width [fs]	Fre-quency [kHz]	Fluence [$J \cdot cm^{-2}$]
5.94	0.45	0.28	1.15	3000.00	5.00	3.00	0.50	1.00	258.00	200.00	1.7
5.94	0.86	0.29	1.20	3000.00	7.00	5.00	0.00	0.50	258.00	200.00	1.7
5.95	0.96	0.77	0.64	3000.00	3.00	5.00	-1.00	0.50	258.00	200.00	8.5
5.95	2.74	0.34	1.43	3000.00	1.00	3.00	0.00	1.00	258.00	200.00	1.7
5.95	0.73	0.94	0.39	3000.00	3.00	5.00	1.00	-0.50	258.00	200.00	17.0
5.95	0.55	0.28	1.15	3000.00	7.00	5.00	1.00	-1.00	258.00	200.00	1.7
5.96	0.82	0.75	0.63	3000.00	3.00	3.00	0.00	0.00	258.00	200.00	8.5
5.97	1.58	0.80	0.67	3000.00	10.00	5.00	-1.00	-0.50	258.00	200.00	8.5
5.97	0.79	0.93	0.39	3000.00	3.00	7.00	1.00	-0.50	258.00	200.00	17.0
5.97	0.87	0.74	0.62	3000.00	5.00	7.00	0.50	0.50	258.00	200.00	8.5
5.97	1.72	0.81	0.68	3000.00	10.00	10.00	-1.00	-0.50	258.00	200.00	8.5
5.98	0.86	0.93	0.39	3000.00	3.00	10.00	1.00	-0.50	258.00	200.00	17.0
5.98	1.32	0.77	0.65	3000.00	5.00	3.00	-1.00	-0.50	258.00	200.00	8.5
5.98	0.94	0.74	0.62	3000.00	3.00	10.00	-1.00	0.50	258.00	200.00	8.5
5.98	1.55	0.79	0.66	3000.00	7.00	5.00	-1.00	-0.50	258.00	200.00	8.5
5.99	1.60	0.79	0.66	3000.00	5.00	5.00	-1.00	-0.50	258.00	200.00	8.5
5.99	1.25	0.76	0.64	3000.00	10.00	3.00	-1.00	0.00	258.00	200.00	8.5
5.99	0.49	0.88	0.37	3000.00	1.00	3.00	0.00	-1.00	258.00	200.00	17.0
5.99	0.50	0.26	1.10	3000.00	3.00	3.00	0.50	0.50	258.00	200.00	1.7
6.00	0.72	0.90	0.38	3000.00	3.00	7.00	1.00	0.00	258.00	200.00	17.0
6.00	1.26	0.76	0.63	3000.00	7.00	10.00	0.50	0.00	258.00	200.00	8.5
6.00	0.75	0.27	1.13	3000.00	3.00	10.00	-1.00	1.00	258.00	200.00	1.7
6.00	1.68	0.79	0.66	3000.00	7.00	7.00	-1.00	-0.50	258.00	200.00	8.5
6.01	1.54	0.77	0.65	3000.00	5.00	10.00	-1.00	0.00	258.00	200.00	8.5
6.01	1.01	0.28	1.15	3000.00	3.00	3.00	-1.00	1.00	258.00	200.00	1.7
6.01	1.99	0.80	0.67	3000.00	5.00	7.00	-1.00	-0.50	258.00	200.00	8.5
6.02	1.55	0.77	0.64	3000.00	5.00	3.00	-0.50	0.00	258.00	200.00	8.5
6.02	1.50	0.76	0.64	3000.00	7.00	10.00	-1.00	0.00	258.00	200.00	8.5
6.02	1.50	0.76	0.64	3000.00	5.00	5.00	-0.50	0.00	258.00	200.00	8.5
6.02	1.21	0.74	0.62	3000.00	3.00	7.00	-1.00	-0.50	258.00	200.00	8.5
6.02	0.33	0.25	1.05	3000.00	3.00	7.00	1.00	-1.00	258.00	200.00	1.7
6.03	0.76	0.26	1.10	3000.00	10.00	5.00	-1.00	1.00	258.00	200.00	1.7

Cost function	Sa [μm]	Ablation rate [mm³·min⁻¹]	Ablation eff. [μm³·μJ⁻¹]	Speed [mm/s]	MHz burst	GHz burst	MHz burst Slope	GHz burst Slope	Pulse Width [fs]	Fre-quency [kHz]	Fluence [J·cm⁻²]
6.03	0.76	0.26	1.10	3000.00	10.00	7.00	1.00	1.00	258.00	200.00	1.7
6.03	0.79	0.26	1.10	3000.00	3.00	7.00	-1.00	-1.00	258.00	200.00	1.7
6.04	0.44	0.25	1.05	3000.00	3.00	5.00	1.00	1.00	258.00	200.00	1.7
6.04	0.31	0.25	1.03	3000.00	7.00	7.00	1.00	-1.00	258.00	200.00	1.7
6.04	1.13	0.27	1.13	3000.00	5.00	7.00	0.50	1.00	258.00	200.00	1.7
6.05	0.53	0.25	1.05	3000.00	7.00	3.00	-1.00	0.50	258.00	200.00	1.7
6.05	0.54	0.25	1.05	3000.00	3.00	5.00	-1.00	0.50	258.00	200.00	1.7
6.05	0.55	0.25	1.05	3000.00	5.00	5.00	0.00	-1.00	258.00	200.00	1.7
6.05	1.23	0.72	0.60	3000.00	7.00	7.00	0.50	0.00	258.00	200.00	8.5
6.06	0.61	0.67	0.56	3000.00	10.00	3.00	1.00	0.00	258.00	200.00	8.5
6.06	1.24	0.71	0.60	3000.00	3.00	5.00	-1.00	-0.50	258.00	200.00	8.5
6.06	0.56	0.66	0.55	3000.00	7.00	3.00	1.00	0.00	258.00	200.00	8.5
6.06	1.10	0.70	0.59	3000.00	5.00	7.00	0.50	0.00	258.00	200.00	8.5
6.06	3.08	0.32	1.35	3000.00	5.00	3.00	-0.50	1.00	258.00	200.00	1.7
6.06	0.74	0.67	0.56	3000.00	3.00	7.00	0.00	0.50	258.00	200.00	8.5
6.06	1.05	0.70	0.58	3000.00	3.00	5.00	0.50	-0.50	258.00	200.00	8.5
6.06	11.28	1.89	0.79	3000.00	7.00	3.00	1.00	-1.00	258.00	200.00	17.0
6.06	0.69	0.25	1.05	3000.00	5.00	5.00	-1.00	-1.00	258.00	200.00	1.7
6.07	0.92	0.26	1.08	3000.00	5.00	10.00	-1.00	1.00	258.00	200.00	1.7
6.07	1.01	0.69	0.58	3000.00	3.00	3.00	-1.00	-0.50	258.00	200.00	8.5
6.07	0.52	0.25	1.03	3000.00	3.00	7.00	-1.00	0.50	258.00	200.00	1.7
6.07	0.79	0.67	0.56	3000.00	10.00	7.00	1.00	-1.00	258.00	200.00	8.5
6.07	0.82	0.67	0.56	3000.00	10.00	10.00	1.00	-1.00	258.00	200.00	8.5
6.07	0.61	0.65	0.55	3000.00	3.00	5.00	0.00	0.50	258.00	200.00	8.5
6.08	0.69	0.66	0.55	3000.00	3.00	5.00	0.50	0.50	258.00	200.00	8.5
6.08	0.93	0.68	0.57	3000.00	3.00	10.00	-0.50	0.50	258.00	200.00	8.5
6.08	0.60	0.25	1.03	3000.00	3.00	10.00	1.00	1.00	258.00	200.00	1.7
6.08	0.82	0.25	1.05	3000.00	10.00	5.00	-1.00	-0.50	258.00	200.00	1.7
6.08	2.83	0.31	1.30	3000.00	10.00	1.00	-1.00	0.00	258.00	200.00	1.7
6.08	1.02	0.26	1.08	3000.00	10.00	1.00	1.00	0.00	258.00	200.00	1.7
6.08	1.26	0.70	0.59	3000.00	7.00	3.00	-1.00	-0.50	258.00	200.00	8.5
6.08	0.63	0.25	1.03	3000.00	5.00	7.00	-1.00	1.00	258.00	200.00	1.7

Cost function	Sa [μm]	Ablation rate [mm³·min⁻¹]	Ablation eff. [μm³·μJ⁻¹]	Speed [mm/s]	MHz burst	GHz burst	MHz burst Slope	GHz burst Slope	Pulse Width [fs]	Fre-quency [kHz]	Fluence [J·cm⁻²]
6.08	0.59	0.65	0.54	3000.00	10.00	7.00	1.00	1.00	258.00	200.00	8.5
6.08	3.06	0.32	1.33	3000.00	5.00	5.00	0.00	1.00	258.00	200.00	1.7
6.08	1.22	0.70	0.58	3000.00	5.00	10.00	0.50	0.00	258.00	200.00	8.5
6.08	1.16	0.69	0.58	3000.00	3.00	10.00	-1.00	-0.50	258.00	200.00	8.5
6.09	0.47	0.24	1.00	3000.00	7.00	7.00	1.00	1.00	258.00	200.00	1.7
6.09	0.79	0.66	0.55	3000.00	10.00	5.00	1.00	-1.00	258.00	200.00	8.5
6.09	0.48	0.24	1.00	3000.00	3.00	5.00	0.00	-1.00	258.00	200.00	1.7
6.09	0.57	0.64	0.54	3000.00	10.00	10.00	1.00	1.00	258.00	200.00	8.5
6.09	0.84	0.83	0.35	3000.00	3.00	10.00	1.00	0.00	258.00	200.00	17.0
6.09	13.56	2.10	0.88	3000.00	10.00	1.00	1.00	0.00	258.00	200.00	17.0
6.09	0.47	0.80	0.33	3000.00	1.00	7.00	0.00	-1.00	258.00	200.00	17.0
6.09	4.65	1.21	0.50	3000.00	10.00	7.00	1.00	0.50	258.00	200.00	17.0
6.09	1.95	0.28	1.18	3000.00	7.00	3.00	-0.50	0.50	258.00	200.00	1.7
6.10	1.58	0.71	0.60	3000.00	10.00	7.00	-1.00	-0.50	258.00	200.00	8.5
6.10	1.60	0.27	1.13	3000.00	5.00	3.00	-0.50	0.50	258.00	200.00	1.7
6.10	0.91	0.66	0.55	3000.00	3.00	5.00	-0.50	0.50	258.00	200.00	8.5
6.10	0.42	0.23	0.98	3000.00	5.00	5.00	1.00	1.00	258.00	200.00	1.7
6.10	0.43	0.23	0.98	3000.00	3.00	5.00	1.00	0.50	258.00	200.00	1.7
6.10	0.43	0.23	0.98	3000.00	5.00	10.00	0.00	-1.00	258.00	200.00	1.7
6.10	0.71	0.64	0.54	3000.00	3.00	7.00	0.50	0.50	258.00	200.00	8.5
6.11	0.90	0.65	0.55	3000.00	3.00	10.00	0.00	0.50	258.00	200.00	8.5
6.11	3.50	0.32	1.35	3000.00	7.00	1.00	0.50	0.00	258.00	200.00	1.7
6.12	0.74	0.64	0.53	3000.00	5.00	3.00	1.00	0.00	258.00	200.00	8.5
6.12	0.74	0.24	1.00	3000.00	3.00	3.00	-1.00	0.50	258.00	200.00	1.7
6.12	1.37	0.68	0.57	3000.00	3.00	3.00	1.00	1.00	258.00	200.00	8.5
6.12	1.01	0.65	0.55	3000.00	3.00	3.00	-0.50	0.00	258.00	200.00	8.5
6.12	1.32	0.68	0.57	3000.00	7.00	3.00	-1.00	0.00	258.00	200.00	8.5
6.12	1.02	0.65	0.55	3000.00	3.00	7.00	0.50	-0.50	258.00	200.00	8.5
6.12	2.60	0.29	1.23	3000.00	5.00	5.00	-0.50	1.00	258.00	200.00	1.7
6.12	0.45	0.77	0.32	3000.00	1.00	5.00	0.00	-1.00	258.00	200.00	17.0
6.13	0.41	0.23	0.95	3000.00	3.00	5.00	0.00	1.00	258.00	200.00	1.7
6.13	0.91	0.64	0.54	3000.00	7.00	7.00	1.00	1.00	258.00	200.00	8.5

Cost function	Sa [μm]	Ablation rate [mm³·min⁻¹]	Ablation eff. [μm³·μJ⁻¹]	Speed [mm/s]	MHz burst	GHz burst	MHz burst Slope	GHz burst Slope	Pulse Width [fs]	Fre-quency [kHz]	Fluence [J·cm⁻²]
6.13	1.29	0.67	0.56	3000.00	3.00	7.00	-1.00	0.00	258.00	200.00	8.5
6.13	1.08	0.65	0.55	3000.00	3.00	10.00	0.50	-0.50	258.00	200.00	8.5
6.13	0.45	0.23	0.95	3000.00	3.00	10.00	0.50	1.00	258.00	200.00	1.7
6.13	0.55	0.77	0.32	3000.00	1.00	3.00	0.00	0.00	258.00	200.00	17.0
6.13	0.72	0.62	0.52	3000.00	7.00	5.00	1.00	1.00	258.00	200.00	8.5
6.13	1.33	0.67	0.56	3000.00	3.00	10.00	-0.50	0.00	258.00	200.00	8.5
6.13	0.47	0.23	0.95	3000.00	5.00	7.00	1.00	1.00	258.00	200.00	1.7
6.13	0.82	0.63	0.53	3000.00	3.00	10.00	0.50	0.50	258.00	200.00	8.5
6.14	0.50	0.23	0.95	3000.00	5.00	3.00	1.00	1.00	258.00	200.00	1.7
6.14	0.34	0.22	0.93	3000.00	3.00	3.00	-0.50	0.00	258.00	200.00	1.7
6.14	0.72	0.62	0.52	3000.00	3.00	5.00	1.00	1.00	258.00	200.00	8.5
6.15	0.58	0.23	0.95	3000.00	3.00	3.00	0.50	-1.00	258.00	200.00	1.7
6.15	0.69	0.61	0.51	3000.00	5.00	5.00	1.00	1.00	258.00	200.00	8.5
6.15	1.24	0.65	0.55	3000.00	5.00	3.00	-1.00	0.00	258.00	200.00	8.5
6.15	0.41	0.22	0.93	3000.00	7.00	3.00	-1.00	-1.00	258.00	200.00	1.7
6.15	2.83	0.29	1.23	3000.00	5.00	1.00	-0.50	0.00	258.00	200.00	1.7
6.15	0.96	0.63	0.53	3000.00	3.00	10.00	0.50	-1.00	258.00	200.00	8.5
6.15	0.77	0.61	0.51	3000.00	10.00	5.00	1.00	0.00	258.00	200.00	8.5
6.16	2.89	0.29	1.23	3000.00	7.00	1.00	-0.50	0.00	258.00	200.00	1.7
6.16	2.69	0.29	1.20	3000.00	10.00	7.00	-0.50	1.00	258.00	200.00	1.7
6.16	1.34	0.65	0.55	3000.00	5.00	5.00	-1.00	0.00	258.00	200.00	8.5
6.17	0.55	0.22	0.93	3000.00	3.00	10.00	0.50	-1.00	258.00	200.00	1.7
6.17	0.55	0.22	0.93	3000.00	3.00	5.00	0.50	0.50	258.00	200.00	1.7
6.17	0.56	0.59	0.49	3000.00	10.00	3.00	1.00	0.50	258.00	200.00	8.5
6.17	1.19	0.24	1.00	3000.00	5.00	1.00	1.00	0.00	258.00	200.00	1.7
6.17	0.97	0.62	0.52	3000.00	3.00	5.00	0.00	0.00	258.00	200.00	8.5
6.17	1.12	0.63	0.53	3000.00	3.00	5.00	-0.50	0.00	258.00	200.00	8.5
6.17	2.60	0.28	1.18	3000.00	5.00	1.00	0.00	0.00	258.00	200.00	1.7
6.17	11.04	1.78	0.74	3000.00	7.00	7.00	1.00	-1.00	258.00	200.00	17.0
6.18	0.94	0.61	0.51	3000.00	3.00	3.00	-1.00	0.00	258.00	200.00	8.5
6.18	0.44	0.22	0.90	3000.00	5.00	3.00	0.50	-1.00	258.00	200.00	1.7
6.18	0.44	0.22	0.90	3000.00	5.00	5.00	0.50	0.50	258.00	200.00	1.7

Cost function	Sa [μm]	Ablation rate [mm³·min⁻¹]	Ablation eff. [μm³·μJ⁻¹]	Speed [mm/s]	MHz burst	GHz burst	MHz burst Slope	GHz burst Slope	Pulse Width [fs]	Fre-quency [kHz]	Fluence [J·cm⁻²]
6.18	0.85	0.23	0.95	3000.00	3.00	5.00	-0.50	-1.00	258.00	200.00	1.7
6.18	0.58	0.58	0.49	3000.00	5.00	3.00	1.00	0.50	258.00	200.00	8.5
6.18	0.47	0.22	0.90	3000.00	3.00	3.00	1.00	-1.00	258.00	200.00	1.7
6.18	1.28	0.24	1.00	3000.00	3.00	3.00	0.00	-1.00	258.00	200.00	1.7
6.18	0.48	0.22	0.90	3000.00	3.00	5.00	0.00	0.50	258.00	200.00	1.7
6.18	1.44	0.65	0.54	3000.00	5.00	7.00	-1.00	0.00	258.00	200.00	8.5
6.18	0.61	0.58	0.49	3000.00	7.00	3.00	1.00	0.50	258.00	200.00	8.5
6.18	0.49	0.22	0.90	3000.00	10.00	3.00	-1.00	0.50	258.00	200.00	1.7
6.18	0.70	0.22	0.93	3000.00	7.00	3.00	-0.50	0.00	258.00	200.00	1.7
6.18	0.47	0.72	0.30	3000.00	1.00	3.00	0.00	-0.50	258.00	200.00	17.0
6.19	0.32	0.21	0.88	3000.00	3.00	3.00	-1.00	-1.00	258.00	200.00	1.7
6.19	0.93	0.23	0.95	3000.00	5.00	10.00	-0.50	0.50	258.00	200.00	1.7
6.19	0.54	0.22	0.90	3000.00	3.00	5.00	-0.50	0.50	258.00	200.00	1.7
6.19	0.47	0.71	0.30	3000.00	1.00	10.00	0.00	-1.00	258.00	200.00	17.0
6.19	0.36	0.21	0.88	3000.00	10.00	3.00	1.00	1.00	258.00	200.00	1.7
6.19	0.92	0.60	0.50	3000.00	3.00	7.00	-0.50	0.50	258.00	200.00	8.5
6.19	2.78	0.28	1.18	3000.00	10.00	1.00	-0.50	0.00	258.00	200.00	1.7
6.19	0.59	0.22	0.90	3000.00	5.00	3.00	-0.50	0.00	258.00	200.00	1.7
6.19	0.79	0.22	0.93	3000.00	5.00	5.00	-1.00	1.00	258.00	200.00	1.7
6.20	0.41	0.21	0.88	3000.00	7.00	5.00	1.00	1.00	258.00	200.00	1.7
6.20	0.67	0.58	0.48	3000.00	10.00	5.00	1.00	1.00	258.00	200.00	8.5
6.20	0.43	0.21	0.88	3000.00	3.00	5.00	-1.00	-0.50	258.00	200.00	1.7
6.20	0.78	0.58	0.49	3000.00	3.00	5.00	0.50	0.00	258.00	200.00	8.5
6.20	1.39	0.63	0.53	3000.00	3.00	10.00	-1.00	0.00	258.00	200.00	8.5
6.20	0.80	0.58	0.49	3000.00	7.00	5.00	1.00	-1.00	258.00	200.00	8.5
6.20	1.26	0.62	0.52	3000.00	3.00	10.00	0.00	0.00	258.00	200.00	8.5
6.21	0.70	0.22	0.90	3000.00	7.00	10.00	1.00	1.00	258.00	200.00	1.7
6.21	1.08	0.60	0.50	3000.00	3.00	5.00	-1.00	0.00	258.00	200.00	8.5
6.21	0.33	0.20	0.85	3000.00	5.00	7.00	-1.00	-1.00	258.00	200.00	1.7
6.21	2.77	0.28	1.15	3000.00	5.00	3.00	0.00	1.00	258.00	200.00	1.7
6.21	0.56	0.21	0.88	3000.00	3.00	3.00	-0.50	-1.00	258.00	200.00	1.7
6.21	1.58	0.64	0.53	3000.00	10.00	10.00	-1.00	0.00	258.00	200.00	8.5

Cost function	Sa [μm]	Ablation rate [mm³·min⁻¹]	Ablation eff. [μm³·μJ⁻¹]	Speed [mm/s]	MHz burst	GHz burst	MHz burst Slope	GHz burst Slope	Pulse Width [fs]	Fre-quency [kHz]	Fluence [J·cm⁻²]
6.21	0.37	0.20	0.85	3000.00	3.00	7.00	0.50	1.00	258.00	200.00	1.7
6.22	0.38	0.20	0.85	3000.00	3.00	5.00	1.00	-0.50	258.00	200.00	1.7
6.22	2.80	0.28	1.15	3000.00	5.00	1.00	-1.00	0.00	258.00	200.00	1.7
6.22	0.39	0.20	0.85	3000.00	3.00	5.00	-1.00	-1.00	258.00	200.00	1.7
6.22	0.71	0.56	0.47	3000.00	5.00	5.00	1.00	0.50	258.00	200.00	8.5
6.22	0.35	0.20	0.83	3000.00	10.00	7.00	1.00	-1.00	258.00	200.00	1.7
6.22	1.21	0.60	0.50	3000.00	10.00	3.00	1.00	1.00	258.00	200.00	8.5
6.23	0.61	0.55	0.46	3000.00	3.00	3.00	1.00	0.50	258.00	200.00	8.5
6.23	1.16	0.59	0.50	3000.00	3.00	7.00	-0.50	0.00	258.00	200.00	8.5
6.23	0.48	0.20	0.85	3000.00	3.00	5.00	0.00	-0.50	258.00	200.00	1.7
6.23	1.25	0.60	0.50	3000.00	10.00	3.00	1.00	-0.50	258.00	200.00	8.5
6.23	1.03	0.58	0.49	3000.00	5.00	5.00	1.00	-1.00	258.00	200.00	8.5
6.23	0.52	0.20	0.85	3000.00	7.00	3.00	1.00	-1.00	258.00	200.00	1.7
6.23	0.61	0.55	0.46	3000.00	7.00	10.00	1.00	1.00	258.00	200.00	8.5
6.24	7.91	1.12	0.94	3000.00	10.00	1.00	1.00	0.00	258.00	200.00	8.5
6.24	0.85	0.56	0.47	3000.00	7.00	3.00	1.00	-1.00	258.00	200.00	8.5
6.24	0.55	0.20	0.85	3000.00	5.00	7.00	-1.00	0.50	258.00	200.00	1.7
6.24	13.51	1.97	0.82	3000.00	5.00	1.00	1.00	0.00	258.00	200.00	17.0
6.24	0.56	0.20	0.85	3000.00	1.00	7.00	0.00	-1.00	258.00	200.00	1.7
6.24	0.37	0.20	0.83	3000.00	5.00	5.00	0.50	-0.50	258.00	200.00	1.7
6.24	0.57	0.20	0.85	3000.00	3.00	5.00	-1.00	0.00	258.00	200.00	1.7
6.24	0.65	0.55	0.46	3000.00	7.00	5.00	1.00	0.00	258.00	200.00	8.5
6.24	0.39	0.20	0.83	3000.00	5.00	3.00	0.50	-0.50	258.00	200.00	1.7
6.24	0.40	0.20	0.83	3000.00	5.00	3.00	-1.00	-0.50	258.00	200.00	1.7
6.24	1.06	0.58	0.48	3000.00	5.00	3.00	1.00	-1.00	258.00	200.00	8.5
6.25	0.72	0.55	0.46	3000.00	5.00	10.00	1.00	1.00	258.00	200.00	8.5
6.25	0.64	0.54	0.45	3000.00	10.00	10.00	1.00	0.50	258.00	200.00	8.5
6.25	0.45	0.20	0.83	3000.00	7.00	7.00	0.00	0.50	258.00	200.00	1.7
6.25	0.45	0.20	0.83	3000.00	7.00	5.00	-1.00	0.50	258.00	200.00	1.7
6.25	0.59	0.53	0.45	3000.00	7.00	7.00	1.00	0.50	258.00	200.00	8.5
6.25	0.88	0.21	0.88	3000.00	5.00	3.00	-1.00	0.00	258.00	200.00	1.7
6.25	0.33	0.19	0.80	3000.00	7.00	7.00	1.00	-0.50	258.00	200.00	1.7

Cost function	Sa [μm]	Ablation rate [mm³·min⁻¹]	Ablation eff. [μm³·μJ⁻¹]	Speed [mm/s]	MHz burst	GHz burst	MHz burst Slope	GHz burst Slope	Pulse Width [fs]	Fre-quency [kHz]	Fluence [J·cm⁻²]
6.25	0.69	0.20	0.85	3000.00	7.00	5.00	-0.50	0.00	258.00	200.00	1.7
6.25	1.44	0.60	0.50	3000.00	7.00	7.00	-1.00	0.00	258.00	200.00	8.5
6.25	0.90	0.21	0.88	3000.00	3.00	10.00	0.00	-1.00	258.00	200.00	1.7
6.25	0.50	0.20	0.83	3000.00	5.00	7.00	-0.50	-1.00	258.00	200.00	1.7
6.25	3.12	0.28	1.15	3000.00	7.00	1.00	0.00	0.00	258.00	200.00	1.7
6.25	0.30	0.19	0.80	3000.00	5.00	3.00	-1.00	-1.00	258.00	200.00	1.7
6.25	0.56	0.53	0.44	3000.00	3.00	3.00	1.00	0.00	258.00	200.00	8.5
6.25	0.51	0.20	0.83	3000.00	7.00	3.00	-0.50	-0.50	258.00	200.00	1.7
6.26	0.53	0.66	0.28	3000.00	1.00	7.00	0.00	-0.50	258.00	200.00	17.0
6.26	1.17	0.58	0.48	3000.00	3.00	7.00	0.00	0.00	258.00	200.00	8.5
6.26	0.72	0.54	0.45	3000.00	7.00	5.00	1.00	0.50	258.00	200.00	8.5
6.26	1.33	0.59	0.49	3000.00	7.00	5.00	-1.00	0.00	258.00	200.00	8.5
6.27	0.82	0.54	0.45	3000.00	5.00	7.00	1.00	-1.00	258.00	200.00	8.5
6.27	0.60	0.52	0.44	3000.00	10.00	7.00	1.00	0.50	258.00	200.00	8.5
6.27	0.64	0.20	0.83	3000.00	10.00	5.00	-1.00	0.00	258.00	200.00	1.7
6.27	0.84	0.54	0.45	3000.00	7.00	7.00	1.00	-1.00	258.00	200.00	8.5
6.27	0.64	0.52	0.44	3000.00	3.00	10.00	1.00	1.00	258.00	200.00	8.5
6.27	1.32	0.58	0.48	3000.00	10.00	5.00	-1.00	0.00	258.00	200.00	8.5
6.27	0.67	0.20	0.83	3000.00	3.00	3.00	0.00	-0.50	258.00	200.00	1.7
6.27	0.79	0.53	0.45	3000.00	5.00	7.00	1.00	1.00	258.00	200.00	8.5
6.28	0.69	0.20	0.83	3000.00	5.00	7.00	0.50	0.50	258.00	200.00	1.7
6.28	0.49	0.19	0.80	3000.00	7.00	5.00	-0.50	-1.00	258.00	200.00	1.7
6.28	1.66	0.60	0.50	3000.00	7.00	3.00	1.00	1.00	258.00	200.00	8.5
6.28	0.90	0.54	0.45	3000.00	10.00	7.00	1.00	0.00	258.00	200.00	8.5
6.28	0.75	0.53	0.44	3000.00	1.00	7.00	0.00	0.50	258.00	200.00	8.5
6.28	0.71	0.20	0.83	3000.00	5.00	3.00	1.00	-0.50	258.00	200.00	1.7
6.28	0.56	0.65	0.27	3000.00	1.00	10.00	0.00	-0.50	258.00	200.00	17.0
6.28	0.51	0.19	0.80	3000.00	1.00	3.00	0.00	-1.00	258.00	200.00	1.7
6.28	0.32	0.19	0.78	3000.00	3.00	5.00	1.00	-1.00	258.00	200.00	1.7
6.28	0.32	0.19	0.78	3000.00	5.00	3.00	0.50	0.50	258.00	200.00	1.7
6.28	0.33	0.19	0.78	3000.00	5.00	3.00	1.00	-1.00	258.00	200.00	1.7
6.28	0.55	0.19	0.80	3000.00	1.00	10.00	0.00	1.00	258.00	200.00	1.7

Cost function	Sa [µm]	Ablation rate [mm³·min⁻¹]	Ablation eff. [µm³·µJ⁻¹]	Speed [mm/s]	MHz burst	GHz burst	MHz burst Slope	GHz burst Slope	Pulse Width [fs]	Fre-quency [kHz]	Fluence [J·cm⁻²]
6.28	0.55	0.19	0.80	3000.00	1.00	5.00	0.00	1.00	258.00	200.00	1.7
6.28	0.55	0.19	0.80	3000.00	3.00	5.00	0.50	-0.50	258.00	200.00	1.7
6.28	0.36	0.19	0.78	3000.00	3.00	10.00	-1.00	-1.00	258.00	200.00	1.7
6.29	0.37	0.19	0.78	3000.00	5.00	5.00	-1.00	-0.50	258.00	200.00	1.7
6.29	0.60	0.19	0.80	3000.00	3.00	10.00	-1.00	0.50	258.00	200.00	1.7
6.29	0.56	0.50	0.42	3000.00	10.00	5.00	1.00	0.50	258.00	200.00	8.5
6.29	3.92	0.77	0.64	3000.00	3.00	7.00	0.50	1.00	258.00	200.00	8.5
6.30	0.46	0.19	0.78	3000.00	10.00	7.00	1.00	0.50	258.00	200.00	1.7
6.30	0.68	0.19	0.80	3000.00	10.00	3.00	1.00	-0.50	258.00	200.00	1.7
6.30	0.73	0.64	0.27	3000.00	1.00	5.00	0.00	0.00	258.00	200.00	17.0
6.30	0.79	0.52	0.43	3000.00	7.00	10.00	1.00	-1.00	258.00	200.00	8.5
6.30	0.49	0.19	0.78	3000.00	3.00	3.00	0.50	0.00	258.00	200.00	1.7
6.30	1.40	0.56	0.47	3000.00	10.00	7.00	-1.00	0.00	258.00	200.00	8.5
6.30	0.66	0.50	0.42	3000.00	5.00	10.00	1.00	0.50	258.00	200.00	8.5
6.30	0.52	0.19	0.78	3000.00	7.00	3.00	-0.50	-1.00	258.00	200.00	1.7
6.30	0.52	0.19	0.78	3000.00	1.00	7.00	0.00	1.00	258.00	200.00	1.7
6.30	0.67	0.50	0.42	3000.00	5.00	5.00	1.00	0.00	258.00	200.00	8.5
6.30	1.13	0.54	0.45	3000.00	7.00	7.00	1.00	-0.50	258.00	200.00	8.5
6.30	0.53	0.19	0.78	3000.00	3.00	3.00	-1.00	0.00	258.00	200.00	1.7
6.31	0.76	0.64	0.27	3000.00	1.00	10.00	0.00	0.00	258.00	200.00	17.0
6.31	0.55	0.19	0.78	3000.00	3.00	3.00	0.50	-0.50	258.00	200.00	1.7
6.31	0.63	0.50	0.42	3000.00	3.00	7.00	1.00	1.00	258.00	200.00	8.5
6.31	1.17	0.54	0.45	3000.00	3.00	10.00	0.50	0.00	258.00	200.00	8.5
6.31	0.62	0.19	0.78	3000.00	3.00	10.00	0.50	0.50	258.00	200.00	1.7
6.32	0.45	0.18	0.75	3000.00	1.00	10.00	0.00	0.50	258.00	200.00	1.7
6.32	0.82	0.50	0.42	3000.00	1.00	10.00	0.00	-1.00	258.00	200.00	8.5
6.32	0.48	0.18	0.75	3000.00	1.00	7.00	0.00	0.50	258.00	200.00	1.7
6.32	0.50	0.18	0.75	3000.00	7.00	7.00	0.50	-0.50	258.00	200.00	1.7
6.33	1.24	0.53	0.45	3000.00	10.00	7.00	1.00	-0.50	258.00	200.00	8.5
6.33	0.87	0.50	0.42	3000.00	3.00	10.00	1.00	0.50	258.00	200.00	8.5
6.33	1.13	0.52	0.44	3000.00	10.00	10.00	1.00	-0.50	258.00	200.00	8.5
6.33	0.36	0.17	0.73	3000.00	3.00	10.00	-0.50	-0.50	258.00	200.00	1.7

Cost function	Sa [μm]	Ablation rate [mm³·min⁻¹]	Ablation eff. [μm³·μJ⁻¹]	Speed [mm/s]	MHz burst	GHz burst	MHz burst Slope	GHz burst Slope	Pulse Width [fs]	Fre-quency [kHz]	Fluence [J·cm⁻²]
6.33	0.36	0.17	0.73	3000.00	3.00	5.00	0.50	-1.00	258.00	200.00	1.7
6.33	2.77	0.25	1.03	3000.00	7.00	5.00	-0.50	1.00	258.00	200.00	1.7
6.33	0.57	0.18	0.75	3000.00	5.00	7.00	0.00	-1.00	258.00	200.00	1.7
6.33	1.15	0.52	0.44	3000.00	10.00	5.00	1.00	-0.50	258.00	200.00	8.5
6.34	0.39	0.17	0.73	3000.00	3.00	3.00	0.00	0.00	258.00	200.00	1.7
6.34	0.95	0.50	0.42	3000.00	7.00	7.00	1.00	0.00	258.00	200.00	8.5
6.34	1.03	0.51	0.43	3000.00	3.00	7.00	1.00	-1.00	258.00	200.00	8.5
6.34	0.61	0.18	0.75	3000.00	7.00	7.00	-1.00	1.00	258.00	200.00	1.7
6.34	0.64	0.18	0.75	3000.00	5.00	5.00	-0.50	-1.00	258.00	200.00	1.7
6.34	0.67	0.60	0.25	3000.00	1.00	7.00	0.00	0.00	258.00	200.00	17.0
6.35	0.48	0.17	0.73	3000.00	7.00	3.00	-1.00	0.00	258.00	200.00	1.7
6.35	0.48	0.17	0.73	3000.00	7.00	3.00	0.50	0.00	258.00	200.00	1.7
6.35	0.35	0.17	0.70	3000.00	5.00	5.00	1.00	-1.00	258.00	200.00	1.7
6.35	0.50	0.17	0.73	3000.00	5.00	5.00	1.00	0.00	258.00	200.00	1.7
6.35	0.89	0.49	0.41	3000.00	5.00	7.00	1.00	0.50	258.00	200.00	8.5
6.35	2.71	0.24	1.00	3000.00	3.00	10.00	-0.50	0.50	258.00	200.00	1.7
6.35	0.50	0.17	0.73	3000.00	3.00	3.00	-0.50	-0.50	258.00	200.00	1.7
6.35	0.91	0.19	0.78	3000.00	7.00	10.00	1.00	0.50	258.00	200.00	1.7
6.35	0.71	0.18	0.75	3000.00	10.00	5.00	-0.50	-0.50	258.00	200.00	1.7
6.35	0.51	0.17	0.73	3000.00	1.00	10.00	0.00	-0.50	258.00	200.00	1.7
6.35	0.54	0.46	0.39	3000.00	3.00	5.00	1.00	0.50	258.00	200.00	8.5
6.35	0.75	0.18	0.75	3000.00	7.00	10.00	-1.00	1.00	258.00	200.00	1.7
6.35	0.35	0.17	0.70	3000.00	3.00	7.00	0.00	-1.00	258.00	200.00	1.7
6.36	0.57	0.17	0.73	3000.00	10.00	10.00	0.50	-1.00	258.00	200.00	1.7
6.36	0.67	0.47	0.39	3000.00	3.00	7.00	1.00	0.50	258.00	200.00	8.5
6.36	0.40	0.17	0.70	3000.00	3.00	10.00	1.00	-0.50	258.00	200.00	1.7
6.36	3.43	0.26	1.08	3000.00	5.00	3.00	0.00	0.50	258.00	200.00	1.7
6.36	1.01	0.49	0.41	3000.00	1.00	10.00	0.00	0.50	258.00	200.00	8.5
6.36	0.71	0.47	0.39	3000.00	7.00	10.00	1.00	0.50	258.00	200.00	8.5
6.36	1.18	0.50	0.42	3000.00	7.00	3.00	1.00	-0.50	258.00	200.00	8.5
6.37	0.46	0.17	0.70	3000.00	3.00	7.00	0.00	-0.50	258.00	200.00	1.7
6.37	0.67	0.17	0.73	3000.00	10.00	3.00	-0.50	-1.00	258.00	200.00	1.7

Cost function	Sa [μm]	Ablation rate [mm³·min⁻¹]	Ablation eff. [μm³·μJ⁻¹]	Speed [mm/s]	MHz burst	GHz burst	MHz burst Slope	GHz burst Slope	Pulse Width [fs]	Fre-quency [kHz]	Fluence [J·cm⁻²]
6.37	0.99	0.49	0.41	3000.00	3.00	7.00	0.50	0.00	258.00	200.00	8.5
6.37	0.71	0.17	0.73	3000.00	5.00	5.00	1.00	0.50	258.00	200.00	1.7
6.37	1.43	0.52	0.43	3000.00	3.00	3.00	1.00	-1.00	258.00	200.00	8.5
6.37	0.90	0.47	0.40	3000.00	10.00	10.00	1.00	0.00	258.00	200.00	8.5
6.38	0.54	0.17	0.70	3000.00	10.00	3.00	1.00	0.50	258.00	200.00	1.7
6.38	0.55	0.17	0.70	3000.00	3.00	5.00	1.00	0.00	258.00	200.00	1.7
6.38	0.92	0.47	0.40	3000.00	5.00	10.00	1.00	-1.00	258.00	200.00	8.5
6.38	0.36	0.16	0.68	3000.00	5.00	3.00	0.00	-1.00	258.00	200.00	1.7
6.38	0.61	0.17	0.70	3000.00	3.00	10.00	1.00	-1.00	258.00	200.00	1.7
6.38	0.41	0.16	0.68	3000.00	5.00	5.00	-1.00	0.00	258.00	200.00	1.7
6.38	0.41	0.16	0.68	3000.00	7.00	5.00	0.50	-1.00	258.00	200.00	1.7
6.38	0.41	0.16	0.68	3000.00	5.00	10.00	1.00	1.00	258.00	200.00	1.7
6.38	0.41	0.16	0.68	3000.00	1.00	3.00	0.00	0.50	258.00	200.00	1.7
6.39	0.42	0.16	0.68	3000.00	3.00	10.00	0.50	0.00	258.00	200.00	1.7
6.39	1.07	0.48	0.40	3000.00	3.00	10.00	1.00	-1.00	258.00	200.00	8.5
6.39	0.45	0.16	0.68	3000.00	5.00	7.00	-1.00	0.00	258.00	200.00	1.7
6.39	0.45	0.16	0.68	3000.00	7.00	7.00	0.50	-1.00	258.00	200.00	1.7
6.39	0.47	0.16	0.68	3000.00	3.00	7.00	1.00	0.00	258.00	200.00	1.7
6.39	0.68	0.17	0.70	3000.00	5.00	7.00	0.50	-1.00	258.00	200.00	1.7
6.39	1.14	0.48	0.40	3000.00	7.00	5.00	1.00	-0.50	258.00	200.00	8.5
6.40	2.52	0.22	0.93	3000.00	5.00	10.00	-0.50	1.00	258.00	200.00	1.7
6.40	0.33	0.16	0.65	3000.00	3.00	10.00	0.00	-0.50	258.00	200.00	1.7
6.40	0.73	0.17	0.70	3000.00	3.00	5.00	-0.50	0.00	258.00	200.00	1.7
6.40	0.74	0.17	0.70	3000.00	3.00	7.00	0.50	-1.00	258.00	200.00	1.7
6.40	1.04	0.47	0.39	3000.00	5.00	5.00	1.00	-0.50	258.00	200.00	8.5
6.40	0.35	0.16	0.65	3000.00	1.00	5.00	0.00	-1.00	258.00	200.00	1.7
6.40	0.35	0.16	0.65	3000.00	7.00	10.00	-1.00	-1.00	258.00	200.00	1.7
6.40	0.36	0.16	0.65	3000.00	10.00	3.00	-1.00	-0.50	258.00	200.00	1.7
6.40	0.77	0.17	0.70	3000.00	3.00	7.00	-0.50	-1.00	258.00	200.00	1.7
6.40	0.78	0.17	0.70	3000.00	5.00	10.00	-1.00	1.00	258.00	200.00	1.7
6.41	0.59	0.16	0.68	3000.00	10.00	5.00	0.50	-0.50	258.00	200.00	1.7
6.41	0.60	0.16	0.68	3000.00	10.00	3.00	-0.50	0.00	258.00	200.00	1.7

Cost function	Sa [μm]	Ablation rate [mm³·min⁻¹]	Ablation eff. [μm³·μJ⁻¹]	Speed [mm/s]	MHz burst	GHz burst	MHz burst Slope	GHz burst Slope	Pulse Width [fs]	Fre-quency [kHz]	Fluence [J·cm⁻²]
6.41	0.82	0.17	0.70	3000.00	3.00	10.00	-0.50	-1.00	258.00	200.00	1.7
6.41	0.67	0.43	0.36	3000.00	1.00	7.00	0.00	-1.00	258.00	200.00	8.5
6.42	12.41	1.71	0.71	3000.00	5.00	3.00	1.00	-1.00	258.00	200.00	17.0
6.42	0.98	0.45	0.38	3000.00	5.00	3.00	1.00	-0.50	258.00	200.00	8.5
6.42	0.31	0.15	0.63	3000.00	1.00	10.00	0.00	-1.00	258.00	200.00	1.7
6.42	0.32	0.15	0.63	3000.00	3.00	7.00	-0.50	-0.50	258.00	200.00	1.7
6.42	0.53	0.16	0.65	3000.00	5.00	10.00	0.50	-1.00	258.00	200.00	1.7
6.42	0.53	0.16	0.65	3000.00	3.00	3.00	1.00	0.50	258.00	200.00	1.7
6.42	0.33	0.15	0.63	3000.00	5.00	10.00	0.00	0.50	258.00	200.00	1.7
6.42	1.95	0.20	0.83	3000.00	7.00	5.00	-0.50	0.50	258.00	200.00	1.7
6.42	0.55	0.16	0.65	3000.00	3.00	5.00	-0.50	-0.50	258.00	200.00	1.7
6.42	0.86	0.44	0.37	3000.00	3.00	3.00	1.00	-0.50	258.00	200.00	8.5
6.42	0.55	0.16	0.65	3000.00	5.00	10.00	-1.00	-0.50	258.00	200.00	1.7
6.42	0.36	0.15	0.63	3000.00	7.00	7.00	-1.00	-1.00	258.00	200.00	1.7
6.43	0.36	0.15	0.63	3000.00	1.00	5.00	0.00	-0.50	258.00	200.00	1.7
6.43	0.37	0.15	0.63	3000.00	7.00	10.00	1.00	-0.50	258.00	200.00	1.7
6.43	0.59	0.16	0.65	3000.00	10.00	7.00	-1.00	1.00	258.00	200.00	1.7
6.43	7.54	0.96	0.80	3000.00	3.00	3.00	0.50	1.00	258.00	200.00	8.5
6.43	1.24	0.46	0.39	3000.00	7.00	10.00	1.00	-0.50	258.00	200.00	8.5
6.43	8.65	1.32	0.55	3000.00	5.00	5.00	1.00	1.00	258.00	200.00	17.0
6.43	0.64	0.16	0.65	3000.00	10.00	5.00	0.00	0.50	258.00	200.00	1.7
6.44	2.25	0.20	0.85	3000.00	10.00	5.00	-0.50	0.50	258.00	200.00	1.7
6.44	0.65	0.16	0.65	3000.00	3.00	10.00	1.00	0.00	258.00	200.00	1.7
6.44	0.46	0.15	0.63	3000.00	5.00	7.00	0.50	0.00	258.00	200.00	1.7
6.44	0.47	0.15	0.63	3000.00	5.00	7.00	-1.00	-0.50	258.00	200.00	1.7
6.44	9.95	1.15	0.96	3000.00	5.00	1.00	1.00	0.00	258.00	200.00	8.5
6.44	0.91	0.16	0.68	3000.00	3.00	7.00	0.50	-0.50	258.00	200.00	1.7
6.44	1.10	0.44	0.37	3000.00	3.00	5.00	1.00	-1.00	258.00	200.00	8.5
6.44	0.52	0.15	0.63	3000.00	7.00	5.00	-1.00	-1.00	258.00	200.00	1.7
6.44	0.52	0.15	0.63	3000.00	5.00	3.00	1.00	0.00	258.00	200.00	1.7
6.45	1.28	0.46	0.38	3000.00	5.00	10.00	1.00	-0.50	258.00	200.00	8.5
6.45	0.35	0.14	0.60	3000.00	7.00	7.00	0.50	0.00	258.00	200.00	1.7

Cost function	Sa [μm]	Ablation rate [mm³·min⁻¹]	Ablation eff. [μm³·μJ⁻¹]	Speed [mm/s]	MHz burst	GHz burst	MHz burst Slope	GHz burst Slope	Pulse Width [fs]	Fre-quency [kHz]	Fluence [J·cm⁻²]
6.45	0.77	0.16	0.65	3000.00	1.00	7.00	0.00	-0.50	258.00	200.00	1.7
6.45	0.59	0.15	0.63	3000.00	1.00	10.00	0.00	0.00	258.00	200.00	1.7
6.45	6.13	0.83	0.70	3000.00	3.00	5.00	0.50	1.00	258.00	200.00	8.5
6.45	0.40	0.14	0.60	3000.00	10.00	5.00	1.00	-0.50	258.00	200.00	1.7
6.46	1.13	0.44	0.37	3000.00	1.00	3.00	0.00	-1.00	258.00	200.00	8.5
6.46	0.42	0.14	0.60	3000.00	5.00	7.00	1.00	-1.00	258.00	200.00	1.7
6.46	0.43	0.14	0.60	3000.00	5.00	3.00	-0.50	-1.00	258.00	200.00	1.7
6.46	0.64	0.15	0.63	3000.00	10.00	7.00	-0.50	-1.00	258.00	200.00	1.7
6.46	0.45	0.14	0.60	3000.00	3.00	7.00	1.00	0.50	258.00	200.00	1.7
6.46	0.95	0.42	0.35	3000.00	3.00	5.00	1.00	-0.50	258.00	200.00	8.5
6.46	0.97	0.42	0.35	3000.00	3.00	10.00	1.00	-0.50	258.00	200.00	8.5
6.46	0.48	0.14	0.60	3000.00	1.00	5.00	0.00	0.50	258.00	200.00	1.7
6.47	0.51	0.14	0.60	3000.00	5.00	5.00	-1.00	0.50	258.00	200.00	1.7
6.47	8.98	1.05	0.88	3000.00	7.00	1.00	1.00	0.00	258.00	200.00	8.5
6.47	0.31	0.14	0.58	3000.00	3.00	3.00	1.00	0.00	258.00	200.00	1.7
6.47	0.33	0.14	0.58	3000.00	5.00	5.00	0.50	-1.00	258.00	200.00	1.7
6.47	0.36	0.14	0.58	3000.00	7.00	7.00	1.00	0.50	258.00	200.00	1.7
6.47	0.56	0.14	0.60	3000.00	7.00	5.00	-1.00	0.00	258.00	200.00	1.7
6.47	1.56	0.17	0.73	3000.00	5.00	7.00	-0.50	0.50	258.00	200.00	1.7
6.47	0.97	0.16	0.65	3000.00	7.00	3.00	0.50	1.00	258.00	200.00	1.7
6.48	0.41	0.14	0.58	3000.00	1.00	3.00	0.00	-0.50	258.00	200.00	1.7
6.48	0.43	0.14	0.58	3000.00	5.00	3.00	-0.50	-0.50	258.00	200.00	1.7
6.48	3.26	0.22	0.93	3000.00	7.00	3.00	0.00	0.50	258.00	200.00	1.7
6.48	0.46	0.14	0.58	3000.00	1.00	5.00	0.00	0.00	258.00	200.00	1.7
6.48	0.46	0.14	0.58	3000.00	3.00	7.00	0.50	0.00	258.00	200.00	1.7
6.49	3.28	0.22	0.93	3000.00	3.00	7.00	0.00	0.50	258.00	200.00	1.7
6.49	0.87	0.15	0.63	3000.00	7.00	10.00	-1.00	0.50	258.00	200.00	1.7
6.49	0.47	0.14	0.58	3000.00	10.00	10.00	0.50	-0.50	258.00	200.00	1.7
6.49	0.87	0.40	0.33	3000.00	3.00	5.00	1.00	0.00	258.00	200.00	8.5
6.49	2.50	0.20	0.83	3000.00	5.00	7.00	-0.50	1.00	258.00	200.00	1.7
6.49	0.69	0.14	0.60	3000.00	5.00	10.00	-0.50	-1.00	258.00	200.00	1.7
6.49	0.70	0.14	0.60	3000.00	10.00	10.00	-1.00	1.00	258.00	200.00	1.7

Cost function	Sa [µm]	Ablation rate [mm³·min⁻¹]	Ablation eff. [µm³·µJ⁻¹]	Speed [mm/s]	MHz burst	GHz burst	MHz burst Slope	GHz burst Slope	Pulse Width [fs]	Fre-quency [kHz]	Fluence [J·cm⁻²]
6.49	0.92	0.15	0.63	3000.00	1.00	3.00	0.00	0.00	258.00	200.00	1.7
6.49	0.52	0.14	0.58	3000.00	7.00	3.00	-1.00	-0.50	258.00	200.00	1.7
6.49	0.95	0.15	0.63	3000.00	5.00	5.00	0.50	0.00	258.00	200.00	1.7
6.50	2.64	0.67	0.28	3000.00	1.00	5.00	0.00	-0.50	258.00	200.00	17.0
6.50	0.96	0.40	0.33	3000.00	3.00	7.00	1.00	-0.50	258.00	200.00	8.5
6.50	0.57	0.14	0.58	3000.00	5.00	7.00	1.00	0.50	258.00	200.00	1.7
6.50	0.76	0.38	0.32	3000.00	1.00	10.00	0.00	0.00	258.00	200.00	8.5
6.50	0.40	0.13	0.55	3000.00	10.00	10.00	1.00	-0.50	258.00	200.00	1.7
6.50	3.62	0.23	0.95	3000.00	5.00	1.00	0.50	0.00	258.00	200.00	1.7
6.50	0.41	0.13	0.55	3000.00	10.00	10.00	-1.00	-1.00	258.00	200.00	1.7
6.50	0.62	0.14	0.58	3000.00	3.00	7.00	0.00	0.00	258.00	200.00	1.7
6.51	0.46	0.13	0.55	3000.00	10.00	10.00	1.00	-1.00	258.00	200.00	1.7
6.51	0.76	0.37	0.31	3000.00	1.00	10.00	0.00	-0.50	258.00	200.00	8.5
6.51	0.68	0.14	0.58	3000.00	5.00	10.00	-0.50	0.00	258.00	200.00	1.7
6.51	0.72	0.14	0.58	3000.00	10.00	10.00	-1.00	0.50	258.00	200.00	1.7
6.51	0.72	0.14	0.58	3000.00	5.00	10.00	-1.00	0.50	258.00	200.00	1.7
6.52	1.13	0.15	0.63	3000.00	7.00	10.00	0.50	0.50	258.00	200.00	1.7
6.52	3.16	0.21	0.88	3000.00	10.00	1.00	0.00	0.00	258.00	200.00	1.7
6.52	0.76	0.14	0.58	3000.00	3.00	7.00	-1.00	0.00	258.00	200.00	1.7
6.52	0.37	0.13	0.53	3000.00	5.00	7.00	0.50	-0.50	258.00	200.00	1.7
6.52	0.38	0.13	0.53	3000.00	10.00	3.00	1.00	0.00	258.00	200.00	1.7
6.53	1.62	0.16	0.68	3000.00	5.00	5.00	-0.50	0.50	258.00	200.00	1.7
6.53	1.13	0.39	0.33	3000.00	1.00	5.00	0.00	-1.00	258.00	200.00	8.5
6.53	0.84	0.37	0.31	3000.00	1.00	7.00	0.00	-0.50	258.00	200.00	8.5
6.53	0.45	0.13	0.53	3000.00	5.00	3.00	0.50	0.00	258.00	200.00	1.7
6.53	0.47	0.13	0.53	3000.00	7.00	5.00	0.00	-1.00	258.00	200.00	1.7
6.53	0.33	0.12	0.50	3000.00	5.00	7.00	1.00	-0.50	258.00	200.00	1.7
6.53	0.35	0.12	0.50	3000.00	7.00	7.00	1.00	0.00	258.00	200.00	1.7
6.53	0.47	0.13	0.53	3000.00	5.00	5.00	1.00	-0.50	258.00	200.00	1.7
6.53	0.48	0.13	0.53	3000.00	7.00	3.00	0.00	-1.00	258.00	200.00	1.7
6.54	0.86	0.36	0.30	3000.00	3.00	10.00	1.00	0.00	258.00	200.00	8.5
6.54	2.36	0.18	0.75	3000.00	10.00	7.00	-0.50	0.50	258.00	200.00	1.7

Cost function	Sa [μm]	Ablation rate [mm³·min⁻¹]	Ablation eff. [μm³·μJ⁻¹]	Speed [mm/s]	MHz burst	GHz burst	MHz burst Slope	GHz burst Slope	Pulse Width [fs]	Fre-quency [kHz]	Fluence [J·cm⁻²]
6.54	0.75	0.13	0.55	3000.00	7.00	3.00	0.50	-1.00	258.00	200.00	1.7
6.54	1.12	0.38	0.32	3000.00	5.00	7.00	1.00	-0.50	258.00	200.00	8.5
6.54	0.82	0.35	0.30	3000.00	1.00	5.00	0.00	0.00	258.00	200.00	8.5
6.55	0.38	0.12	0.50	3000.00	5.00	3.00	1.00	0.50	258.00	200.00	1.7
6.55	0.39	0.12	0.50	3000.00	3.00	5.00	0.00	0.00	258.00	200.00	1.7
6.55	1.20	0.14	0.60	3000.00	3.00	5.00	0.50	0.00	258.00	200.00	1.7
6.55	0.41	0.12	0.50	3000.00	10.00	7.00	1.00	-0.50	258.00	200.00	1.7
6.55	0.83	0.13	0.55	3000.00	10.00	7.00	0.00	-1.00	258.00	200.00	1.7
6.55	0.45	0.12	0.50	3000.00	10.00	5.00	0.50	-1.00	258.00	200.00	1.7
6.56	0.35	0.11	0.48	3000.00	5.00	7.00	1.00	0.00	258.00	200.00	1.7
6.56	3.48	0.21	0.88	3000.00	3.00	10.00	0.00	0.50	258.00	200.00	1.7
6.56	0.86	0.35	0.29	3000.00	7.00	10.00	1.00	0.00	258.00	200.00	8.5
6.56	0.81	0.34	0.29	3000.00	1.00	5.00	0.00	-0.50	258.00	200.00	8.5
6.56	0.94	0.13	0.55	3000.00	3.00	3.00	1.00	-0.50	258.00	200.00	1.7
6.57	1.25	0.37	0.31	3000.00	1.00	7.00	0.00	0.00	258.00	200.00	8.5
6.57	0.38	0.11	0.48	3000.00	7.00	10.00	0.00	0.50	258.00	200.00	1.7
6.57	0.62	0.12	0.50	3000.00	1.00	7.00	0.00	0.00	258.00	200.00	1.7
6.57	0.63	0.12	0.50	3000.00	5.00	7.00	-0.50	-0.50	258.00	200.00	1.7
6.58	0.67	0.12	0.50	3000.00	5.00	5.00	0.00	0.00	258.00	200.00	1.7
6.58	0.98	0.34	0.29	3000.00	5.00	10.00	1.00	0.00	258.00	200.00	8.5
6.59	0.33	0.11	0.45	3000.00	7.00	3.00	1.00	0.00	258.00	200.00	1.7
6.59	0.33	0.11	0.45	3000.00	3.00	10.00	0.50	-0.50	258.00	200.00	1.7
6.59	3.15	0.19	0.80	3000.00	7.00	7.00	0.00	1.00	258.00	200.00	1.7
6.59	0.35	0.11	0.45	3000.00	3.00	7.00	1.00	-0.50	258.00	200.00	1.7
6.59	0.59	0.11	0.48	3000.00	5.00	5.00	0.00	-0.50	258.00	200.00	1.7
6.60	0.90	0.32	0.27	3000.00	1.00	3.00	0.00	0.00	258.00	200.00	8.5
6.60	0.62	0.11	0.48	3000.00	7.00	3.00	1.00	1.00	258.00	200.00	1.7
6.60	2.46	0.17	0.70	3000.00	5.00	7.00	0.00	0.50	258.00	200.00	1.7
6.60	0.67	0.11	0.48	3000.00	5.00	10.00	-1.00	0.00	258.00	200.00	1.7
6.60	2.08	0.16	0.65	3000.00	10.00	3.00	-0.50	0.50	258.00	200.00	1.7
6.60	3.10	0.19	0.78	3000.00	7.00	3.00	-0.50	1.00	258.00	200.00	1.7
6.61	3.12	0.19	0.78	3000.00	7.00	10.00	0.50	1.00	258.00	200.00	1.7

Cost function	Sa [μm]	Ablation rate [mm³·min⁻¹]	Ablation eff. [μm³·μJ⁻¹]	Speed [mm/s]	MHz burst	GHz burst	MHz burst Slope	GHz burst Slope	Pulse Width [fs]	Frequency [kHz]	Fluence [J·cm⁻²]
6.61	0.76	0.31	0.26	3000.00	1.00	3.00	0.00	-0.50	258.00	200.00	8.5
6.61	0.71	0.11	0.48	3000.00	7.00	7.00	0.00	-1.00	258.00	200.00	1.7
6.61	0.52	0.11	0.45	3000.00	3.00	7.00	0.50	0.50	258.00	200.00	1.7
6.61	0.93	0.12	0.50	3000.00	5.00	7.00	-0.50	0.00	258.00	200.00	1.7
6.61	0.33	0.10	0.43	3000.00	10.00	5.00	1.00	1.00	258.00	200.00	1.7
6.61	0.79	0.31	0.26	3000.00	5.00	7.00	1.00	0.00	258.00	200.00	8.5
6.61	0.34	0.10	0.43	3000.00	7.00	5.00	0.50	0.00	258.00	200.00	1.7
6.61	0.95	0.12	0.50	3000.00	10.00	10.00	1.00	1.00	258.00	200.00	1.7
6.61	0.57	0.11	0.45	3000.00	10.00	7.00	-1.00	0.50	258.00	200.00	1.7
6.62	0.85	0.31	0.26	3000.00	3.00	7.00	1.00	0.00	258.00	200.00	8.5
6.62	0.40	0.10	0.43	3000.00	10.00	7.00	-1.00	-0.50	258.00	200.00	1.7
6.62	0.81	0.11	0.48	3000.00	5.00	5.00	-0.50	0.00	258.00	200.00	1.7
6.62	1.01	0.12	0.50	3000.00	10.00	10.00	0.00	-1.00	258.00	200.00	1.7
6.62	3.24	0.19	0.78	3000.00	10.00	1.00	0.50	0.00	258.00	200.00	1.7
6.62	0.44	0.10	0.43	3000.00	10.00	7.00	0.50	0.00	258.00	200.00	1.7
6.63	0.47	0.10	0.43	3000.00	10.00	7.00	-1.00	-1.00	258.00	200.00	1.7
6.63	0.31	0.10	0.40	3000.00	5.00	10.00	1.00	-1.00	258.00	200.00	1.7
6.63	0.51	0.10	0.43	3000.00	10.00	10.00	-1.00	-0.50	258.00	200.00	1.7
6.63	3.13	0.18	0.75	3000.00	7.00	3.00	0.00	1.00	258.00	200.00	1.7
6.63	0.33	0.10	0.40	3000.00	7.00	7.00	-1.00	0.00	258.00	200.00	1.7
6.64	0.58	0.10	0.43	3000.00	10.00	10.00	1.00	0.50	258.00	200.00	1.7
6.64	0.98	0.11	0.48	3000.00	10.00	7.00	0.50	-0.50	258.00	200.00	1.7
6.64	3.21	0.18	0.75	3000.00	7.00	5.00	0.00	1.00	258.00	200.00	1.7
6.64	0.40	0.10	0.40	3000.00	3.00	7.00	-1.00	-0.50	258.00	200.00	1.7
6.65	0.45	0.10	0.40	3000.00	7.00	5.00	1.00	0.00	258.00	200.00	1.7
6.65	0.45	0.10	0.40	3000.00	3.00	3.00	-1.00	-0.50	258.00	200.00	1.7
6.65	0.68	0.10	0.43	3000.00	5.00	5.00	-0.50	-0.50	258.00	200.00	1.7
6.66	0.71	0.10	0.43	3000.00	7.00	10.00	-1.00	-0.50	258.00	200.00	1.7
6.66	0.53	0.10	0.40	3000.00	3.00	10.00	1.00	0.50	258.00	200.00	1.7
6.66	0.34	0.09	0.38	3000.00	7.00	3.00	1.00	-0.50	258.00	200.00	1.7
6.66	0.35	0.09	0.38	3000.00	10.00	5.00	1.00	-1.00	258.00	200.00	1.7
6.67	0.61	0.10	0.40	3000.00	10.00	5.00	-0.50	-1.00	258.00	200.00	1.7

Cost function	Sa [μm]	Ablation rate [mm³·min⁻¹]	Ablation eff. [μm³·μJ⁻¹]	Speed [mm/s]	MHz burst	GHz burst	MHz burst Slope	GHz burst Slope	Pulse Width [fs]	Fre-quency [kHz]	Fluence [J·cm⁻²]
6.67	0.42	0.09	0.38	3000.00	7.00	10.00	0.00	-1.00	258.00	200.00	1.7
6.67	0.45	0.09	0.38	3000.00	5.00	3.00	0.00	-0.50	258.00	200.00	1.7
6.67	0.65	0.10	0.40	3000.00	7.00	3.00	0.00	0.00	258.00	200.00	1.7
6.67	0.47	0.09	0.38	3000.00	10.00	7.00	1.00	0.00	258.00	200.00	1.7
6.67	0.48	0.09	0.38	3000.00	3.00	10.00	-1.00	0.00	258.00	200.00	1.7
6.68	0.71	0.10	0.40	3000.00	10.00	5.00	-0.50	0.00	258.00	200.00	1.7
6.68	2.94	0.16	0.68	3000.00	10.00	5.00	-0.50	1.00	258.00	200.00	1.7
6.68	0.76	0.10	0.40	3000.00	3.00	10.00	-1.00	-0.50	258.00	200.00	1.7
6.69	0.62	0.09	0.38	3000.00	10.00	5.00	0.00	-0.50	258.00	200.00	1.7
6.69	1.24	0.11	0.45	3000.00	10.00	3.00	0.00	0.50	258.00	200.00	1.7
6.70	0.89	0.10	0.40	3000.00	10.00	3.00	1.00	-1.00	258.00	200.00	1.7
6.70	0.50	0.08	0.35	3000.00	7.00	7.00	-1.00	0.50	258.00	200.00	1.7
6.70	0.33	0.08	0.33	3000.00	7.00	10.00	0.50	-1.00	258.00	200.00	1.7
6.71	0.54	0.08	0.35	3000.00	7.00	10.00	1.00	0.00	258.00	200.00	1.7
6.71	0.55	0.08	0.35	3000.00	7.00	7.00	-1.00	-0.50	258.00	200.00	1.7
6.71	0.78	0.09	0.38	3000.00	10.00	7.00	0.50	-1.00	258.00	200.00	1.7
6.72	0.83	0.09	0.38	3000.00	10.00	7.00	0.00	-0.50	258.00	200.00	1.7
6.72	4.05	0.19	0.78	3000.00	7.00	5.00	0.50	1.00	258.00	200.00	1.7
6.72	0.66	0.08	0.35	3000.00	3.00	10.00	0.00	0.00	258.00	200.00	1.7
6.72	1.49	0.11	0.45	3000.00	7.00	10.00	-0.50	0.50	258.00	200.00	1.7
6.73	0.51	0.08	0.33	3000.00	10.00	10.00	0.00	0.00	258.00	200.00	1.7
6.73	0.72	0.08	0.35	3000.00	7.00	5.00	1.00	-0.50	258.00	200.00	1.7
6.73	4.68	0.53	0.45	3000.00	1.00	5.00	0.00	0.50	258.00	200.00	8.5
6.73	0.96	0.09	0.38	3000.00	7.00	7.00	0.00	0.00	258.00	200.00	1.7
6.73	0.38	0.07	0.30	3000.00	10.00	5.00	1.00	0.00	258.00	200.00	1.7
6.73	3.60	0.17	0.70	3000.00	7.00	7.00	0.50	1.00	258.00	200.00	1.7
6.73	0.59	0.08	0.33	3000.00	7.00	5.00	1.00	0.50	258.00	200.00	1.7
6.74	0.80	0.08	0.35	3000.00	7.00	10.00	-0.50	0.00	258.00	200.00	1.7
6.74	0.60	0.08	0.33	3000.00	10.00	10.00	0.50	0.50	258.00	200.00	1.7
6.74	0.41	0.07	0.30	3000.00	5.00	10.00	1.00	-0.50	258.00	200.00	1.7
6.74	7.04	0.71	0.60	3000.00	1.00	5.00	0.00	1.00	258.00	200.00	8.5
6.74	0.67	0.08	0.33	3000.00	7.00	5.00	-0.50	-0.50	258.00	200.00	1.7

Cost function	Sa [μm]	Ablation rate [mm³·min⁻¹]	Ablation eff. [μm³·μJ⁻¹]	Speed [mm/s]	MHz burst	GHz burst	MHz burst Slope	GHz burst Slope	Pulse Width [fs]	Fre-quency [kHz]	Fluence [J·cm⁻²]
6.75	3.30	0.16	0.65	3000.00	5.00	7.00	0.00	1.00	258.00	200.00	1.7
6.75	0.70	0.08	0.33	3000.00	7.00	10.00	-1.00	0.00	258.00	200.00	1.7
6.75	0.50	0.07	0.30	3000.00	7.00	5.00	-1.00	-0.50	258.00	200.00	1.7
6.75	0.32	0.07	0.28	3000.00	5.00	10.00	1.00	0.00	258.00	200.00	1.7
6.75	3.14	0.15	0.63	3000.00	10.00	7.00	0.00	1.00	258.00	200.00	1.7
6.75	3.35	0.16	0.65	3000.00	5.00	10.00	0.00	1.00	258.00	200.00	1.7
6.75	17.17	1.89	0.79	3000.00	3.00	1.00	1.00	0.00	258.00	200.00	17.0
6.76	0.57	0.07	0.30	3000.00	5.00	10.00	0.50	0.00	258.00	200.00	1.7
6.76	0.37	0.07	0.28	3000.00	7.00	7.00	-0.50	-1.00	258.00	200.00	1.7
6.76	0.59	0.07	0.30	3000.00	10.00	3.00	0.00	0.00	258.00	200.00	1.7
6.76	0.61	0.07	0.30	3000.00	10.00	10.00	0.00	0.50	258.00	200.00	1.7
6.77	3.07	0.14	0.60	3000.00	10.00	3.00	-0.50	1.00	258.00	200.00	1.7
6.77	0.50	0.07	0.28	3000.00	10.00	3.00	0.50	-1.00	258.00	200.00	1.7
6.79	6.92	0.67	0.56	3000.00	1.00	7.00	0.00	1.00	258.00	200.00	8.5
6.79	0.64	0.07	0.28	3000.00	7.00	10.00	0.50	-0.50	258.00	200.00	1.7
6.79	3.65	0.16	0.65	3000.00	5.00	5.00	0.00	0.50	258.00	200.00	1.7
6.79	9.91	1.14	0.48	3000.00	3.00	10.00	1.00	1.00	258.00	200.00	17.0
6.79	0.45	0.06	0.25	3000.00	7.00	5.00	0.50	-0.50	258.00	200.00	1.7
6.79	0.46	0.06	0.25	3000.00	10.00	3.00	0.00	-0.50	258.00	200.00	1.7
6.79	0.47	0.06	0.25	3000.00	7.00	5.00	0.00	0.00	258.00	200.00	1.7
6.79	0.69	0.07	0.28	3000.00	7.00	3.00	0.50	-0.50	258.00	200.00	1.7
6.80	0.51	0.06	0.25	3000.00	10.00	7.00	0.00	0.50	258.00	200.00	1.7
6.80	0.54	0.06	0.25	3000.00	7.00	7.00	0.00	-0.50	258.00	200.00	1.7
6.80	0.78	0.07	0.28	3000.00	10.00	7.00	-1.00	0.00	258.00	200.00	1.7
6.81	2.02	0.10	0.43	3000.00	3.00	7.00	-0.50	0.00	258.00	200.00	1.7
6.81	0.42	0.05	0.23	3000.00	10.00	3.00	0.50	-0.50	258.00	200.00	1.7
6.81	0.45	0.05	0.23	3000.00	5.00	10.00	0.50	-0.50	258.00	200.00	1.7
6.81	0.67	0.06	0.25	3000.00	7.00	7.00	-0.50	-0.50	258.00	200.00	1.7
6.82	0.68	0.06	0.25	3000.00	7.00	10.00	0.50	0.00	258.00	200.00	1.7
6.82	0.69	0.06	0.25	3000.00	10.00	7.00	-0.50	-0.50	258.00	200.00	1.7
6.82	3.72	0.15	0.63	3000.00	5.00	10.00	0.50	1.00	258.00	200.00	1.7
6.82	1.92	0.10	0.40	3000.00	5.00	10.00	0.50	0.50	258.00	200.00	1.7

Cost function	Sa [μm]	Ablation rate [mm³·min⁻¹]	Ablation eff. [μm³·μJ⁻¹]	Speed [mm/s]	MHz burst	GHz burst	MHz burst Slope	GHz burst Slope	Pulse Width [fs]	Fre-quency [kHz]	Fluence [J·cm⁻²]
6.83	3.20	0.13	0.55	3000.00	10.00	7.00	0.50	1.00	258.00	200.00	1.7
6.83	0.42	0.05	0.20	3000.00	10.00	5.00	0.00	0.00	258.00	200.00	1.7
6.84	0.47	0.05	0.20	3000.00	7.00	3.00	0.50	0.50	258.00	200.00	1.7
6.84	3.08	0.13	0.53	3000.00	10.00	5.00	0.00	1.00	258.00	200.00	1.7
6.84	0.72	0.05	0.23	3000.00	5.00	10.00	0.00	0.00	258.00	200.00	1.7
6.85	0.77	0.05	0.23	3000.00	10.00	3.00	-0.50	-0.50	258.00	200.00	1.7
6.85	7.06	0.81	0.34	3000.00	1.00	5.00	0.00	0.50	258.00	200.00	17.0
6.85	7.25	0.65	0.55	3000.00	1.00	3.00	0.00	1.00	258.00	200.00	8.5
6.86	2.46	0.10	0.43	3000.00	7.00	7.00	-0.50	0.50	258.00	200.00	1.7
6.86	3.48	0.13	0.55	3000.00	7.00	7.00	0.50	0.50	258.00	200.00	1.7
6.87	0.76	0.05	0.20	3000.00	7.00	5.00	0.00	-0.50	258.00	200.00	1.7
6.88	0.42	0.04	0.15	3000.00	10.00	5.00	0.50	0.00	258.00	200.00	1.7
6.88	0.43	0.04	0.15	3000.00	10.00	7.00	0.00	0.00	258.00	200.00	1.7
6.88	1.43	0.07	0.28	3000.00	10.00	10.00	0.50	1.00	258.00	200.00	1.7
6.89	0.67	0.04	0.18	3000.00	10.00	7.00	-0.50	0.00	258.00	200.00	1.7
6.89	2.72	0.10	0.43	3000.00	7.00	10.00	-0.50	1.00	258.00	200.00	1.7
6.90	0.77	0.04	0.18	3000.00	7.00	10.00	-0.50	-1.00	258.00	200.00	1.7
6.90	0.38	0.03	0.13	3000.00	7.00	3.00	1.00	0.50	258.00	200.00	1.7
6.91	0.84	0.04	0.18	3000.00	10.00	5.00	0.00	-1.00	258.00	200.00	1.7
6.91	0.71	0.04	0.15	3000.00	7.00	10.00	0.00	0.00	258.00	200.00	1.7
6.92	0.53	0.03	0.13	3000.00	10.00	10.00	-1.00	0.00	258.00	200.00	1.7
6.92	0.60	0.03	0.13	3000.00	10.00	10.00	0.00	-0.50	258.00	200.00	1.7
6.93	7.00	0.58	0.49	3000.00	1.00	10.00	0.00	1.00	258.00	200.00	8.5
6.94	10.58	1.08	0.45	3000.00	3.00	7.00	1.00	1.00	258.00	200.00	17.0
6.94	0.76	0.03	0.13	3000.00	5.00	10.00	-0.50	-0.50	258.00	200.00	1.7
6.94	0.77	0.03	0.13	3000.00	5.00	10.00	0.00	-0.50	258.00	200.00	1.7
6.96	0.73	0.02	0.10	3000.00	5.00	10.00	1.00	0.50	258.00	200.00	1.7
6.96	1.54	0.05	0.20	3000.00	10.00	7.00	0.50	0.50	258.00	200.00	1.7
6.97	0.95	0.03	0.13	3000.00	10.00	5.00	1.00	0.50	258.00	200.00	1.7
6.98	1.44	0.04	0.18	3000.00	10.00	10.00	-0.50	-0.50	258.00	200.00	1.7
6.98	0.50	0.01	0.05	3000.00	10.00	10.00	-0.50	-1.00	258.00	200.00	1.7
6.99	0.53	0.01	0.05	3000.00	7.00	10.00	0.00	-0.50	258.00	200.00	1.7

Cost function	Sa [μm]	Ablation rate [$mm^3 \cdot min^{-1}$]	Ablation eff. [$\mu m^3 \cdot \mu J^{-1}$]	Speed [mm/s]	MHz burst	GHz burst	MHz burst Slope	GHz burst Slope	Pulse Width [fs]	Fre-quency [kHz]	Fluence [$J \cdot cm^{-2}$]
7.00	1.01	0.02	0.10	3000.00	10.00	3.00	0.00	-1.00	258.00	200.00	1.7
7.00	3.03	0.08	0.35	3000.00	7.00	5.00	0.50	0.50	258.00	200.00	1.7
7.00	0.63	0.01	0.05	3000.00	10.00	10.00	0.50	0.00	258.00	200.00	1.7
7.01	3.35	0.09	0.38	3000.00	10.00	5.00	0.50	1.00	258.00	200.00	1.7
7.01	0.57	0.01	0.03	3000.00	7.00	3.00	0.00	-0.50	258.00	200.00	1.7
7.02	0.78	0.01	0.05	3000.00	5.00	7.00	0.00	0.00	258.00	200.00	1.7
7.02	0.61	0.01	0.03	3000.00	7.00	10.00	-0.50	-0.50	258.00	200.00	1.7
7.03	7.80	0.73	0.30	3000.00	1.00	10.00	0.00	0.50	258.00	200.00	17.0
7.04	0.97	0.01	0.05	3000.00	7.00	7.00	-0.50	0.00	258.00	200.00	1.7
7.04	0.59	0.00	0.00	3000.00	10.00	10.00	-0.50	0.00	258.00	200.00	1.7
7.04	0.61	0.00	0.00	3000.00	5.00	7.00	0.00	-0.50	258.00	200.00	1.7
7.05	3.47	0.08	0.35	3000.00	10.00	3.00	0.50	1.00	258.00	200.00	1.7
7.06	3.16	0.07	0.30	3000.00	10.00	3.00	0.50	0.50	258.00	200.00	1.7
7.06	3.18	0.07	0.30	3000.00	10.00	5.00	0.50	0.50	258.00	200.00	1.7
7.06	1.58	0.02	0.10	3000.00	10.00	10.00	-0.50	0.50	258.00	200.00	1.7
7.06	2.59	0.05	0.23	3000.00	10.00	10.00	-0.50	1.00	258.00	200.00	1.7
7.07	2.85	0.06	0.25	3000.00	7.00	7.00	-0.50	1.00	258.00	200.00	1.7
7.07	0.85	0.00	0.00	3000.00	10.00	3.00	0.50	0.00	258.00	200.00	1.7
7.12	3.05	0.05	0.23	3000.00	7.00	10.00	0.00	1.00	258.00	200.00	1.7
7.12	3.08	0.05	0.23	3000.00	10.00	3.00	0.00	1.00	258.00	200.00	1.7
7.12	11.82	1.05	0.44	3000.00	3.00	5.00	1.00	1.00	258.00	200.00	17.0
7.16	8.27	0.53	0.44	3000.00	1.00	3.00	0.00	0.50	258.00	200.00	8.5
7.17	13.56	1.18	0.49	3000.00	1.00	3.00	0.00	1.00	258.00	200.00	17.0
7.24	9.93	0.76	0.32	3000.00	1.00	7.00	0.00	0.50	258.00	200.00	17.0
7.25	10.25	0.62	0.52	3000.00	5.00	3.00	1.00	1.00	258.00	200.00	8.5
7.25	3.20	0.02	0.10	3000.00	10.00	10.00	0.00	1.00	258.00	200.00	1.7
7.30	3.37	0.02	0.08	3000.00	3.00	10.00	-0.50	0.00	258.00	200.00	1.7
7.30	13.87	1.10	0.46	3000.00	3.00	3.00	1.00	1.00	258.00	200.00	17.0
7.43	14.40	1.04	0.43	3000.00	1.00	5.00	0.00	1.00	258.00	200.00	17.0
7.47	13.32	0.90	0.37	3000.00	1.00	10.00	0.00	1.00	258.00	200.00	17.0
7.50	13.81	0.93	0.39	3000.00	1.00	7.00	0.00	1.00	258.00	200.00	17.0
